# Triple-Columned and Multiple-Layered 3D Polymers: Design, Synthesis, Aggregation-Induced Emission (AIE), and Computational Study

**DOI:** 10.34133/2021/3565791

**Published:** 2021-02-08

**Authors:** Guanzhao Wu, Yangxue Liu, Zhen Yang, Liulei Ma, Yao Tang, Xianliang Zhao, Hossein Rouh, Qixuan Zheng, Peng Zhou, Jia-Yin Wang, Farhan Siddique, Sai Zhang, Shengzhou Jin, Daniel Unruh, Adelia J. A. Aquino, Hans Lischka, Kristin M. Hutchins, Guigen Li

**Affiliations:** ^1^Department of Chemistry and Biochemistry, Texas Tech University, Lubbock, Texas 79409-1061, USA; ^2^Institute of Chemistry and Biomedical Sciences, School of Chemistry and Chemical Engineering, Nanjing University, Nanjing 210093, China; ^3^School of Pharmaceutical Sciences and Technology, Tianjin University, Tianjin 300072, China; ^4^Department of Mechanical Engineering, Texas Tech University, Lubbock, TX 79409, USA

## Abstract

Conjugated polymers and oligomers have great potentials in various fields, especially in materials and biological sciences because of their intriguing electronic and optoelectronic properties. In recent years, the through-space conjugation system has emerged as a new assembled pattern of multidimensional polymers. Here, a novel series of structurally condensed multicolumn/multilayer 3D polymers and oligomers have been designed and synthesized through one-pot Suzuki polycondensation (SPC). The intramolecularly stacked arrangement of polymers can be supported by either X-ray structural analysis or computational analysis. In all cases, polymers were obtained with modest to good yields, as determined by GPC and ^1^H-NMR. MALDI-TOF analysis has proven the speculation of the step-growth process of this polymerization. The computational study of ab initio and DFT calculations based on trimer and pentamer models gives details of the structures and the electronic transition. Experimental results of optical and AIE research confirmed by calculation indicates that the present work would facilitate the research and applications in materials.

## 1. Introduction

Molecular design has been playing a key role in polymer and material sciences in searching for desired chemical, physical, and biological properties [1–8]. This is particularly applicable to the research on conductive polymers, which has been among the most active topics in the past two decades [9–14]. Properties of conductive polymers are mainly attributed to the conjugation through monomeric connections by C-C double or triple bonds for delocalizing *π*-electrons throughout their backbones [15–17]. Besides traditional bonding pathways, the through-space conjugation has emerged as an alternative fashion for energy and charge transfers in polymers [11, 18, 19]. For example, the latest through-space electronic transfer was developed for organic light emitting devices by using XPT and XtBuCT as dopants which enabled electroluminescence external quantum efficiencies as high as 10% [20]. A similar through-space transfer through singlet fission (SF) involved absorption of photons by two electronically interacting chromophores to produce a singlet exciton state followed by rapid formation of two triplet excitons [21]. In the meanwhile, charge-transfer pathways hybridizing *σ*, *π*-, and through-space interactions have been proven to be feasible by carefully designing monomeric structures for poly- or copolymerizations [4, 6, 22]. These conjugated polymers displayed a series of electronic and optoelectronic properties, including aggregation-induced emission (AIE)/aggregation-enhanced emission (AEE), conduction, thermally activated delayed fluorescence, optical nonlinearity, bipolar charge transport, and multichannel and photocatalysis [18]. In biological systems, energetics of the H^+^/2e^−^ transferring was found to exist between planar nicotinamide adenine dinucleotide (NADH) and protein-bound flavin (FMN) cofactors [23].

**Figure 1:**
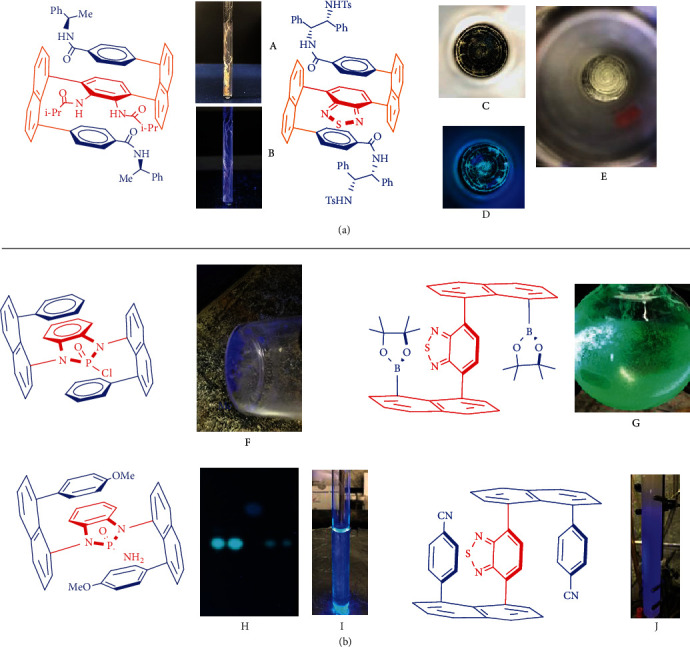
(a) Multilayer 3D chiral sandwich-shaped molecules and macro chirality phenomenon: images under natural light with black background (A and C: compound was dried under air evaporation; E: compound was dried under rotavapor); image under UV light (365 nm) (B and D). (b) Multilayer 3D chiral sandwich-shaped molecules and fluorescence images under UV light (365 nm) (F–J).

Very recently, our labs have reported the work on multilayer 3D chirality of *C*_2_- and/or pseudo *C*_2_-symmetry as new chirality consisting of three layers: top, middle, and bottom layers [24–27]. It shows that the top and bottom layers restrict each other from free axial rotation, i.e., if either top or bottom layer is removed, this 3D chirality would not exist. The resulting multilayer 3D chiral products displayed macro chirality phenomena that can be directly observed with the naked eyes without the aid of microscopic devices (Figure 1(a)). For example, when CDCl_3_-solution containing a 3D compound was slowly evaporated at room temperature for several weeks, right-handed and spirotextile-shaped solids were formed inside NMR tubes (Figure 1(a), A and B). Nearly all 3D products displayed strong fluorescence in both solid and solution forms, on TLC plates and in silica gel columns (Figure 1(b)).

We also found that changing functional groups in multilayer 3D chiral molecules resulted in the change of their luminescence colors from gold to deep blue under UV irradiation. These molecules presented strong aggregation-induced emission (AIE) [28–32], in which the higher the fraction of water, the stronger the luminescence. Upon irradiation under polarized light, some chiral 3D molecules showed abnormally high optical rotation; this has been very rare in known chiral compounds, indicating their potentials for the development of optical materials and devices in the future.

Our next ambition is to render multilayer chiral 3D polymers by using the above monomeric motifs. In fact, these chiral polymers have not been documented in literature yet. Obviously, our initial strategy should be started from the design and synthesis of their counterparts, including achiral or racemic multilayer polymers. The literature search reveals that multiple-layered polymers have been established and displayed various attractive properties. For example, Nakano reported poly-(dibenzofulvene)s with *π*-stacked conformers displaying higher hole drift mobility than the through-bond conjugated poly(phenylenevinylene) [10]. Morisaki and Chujo synthesized [2.2]paracyclophane-layered polymers by attaching fluorescence quenchers at the ends of polymer chains, and they found that these properties are attributed to photoexcited energy transporting from the layered [2.2]paracyclophane to terminal structural units [15a, 21]. Jagtap and Collard reported 2D multilayered *π*-stacked conjugated polymers by using U-turn pseudogeminal[2.2]paracyclophane scaffolds, and they found that these polymers showed broad fluorescence emission and large Stokes shift as comparing to model unstacked linear analogs [33–35]. However, the above multilayer polymers are anchored with either longer bridges or expended bridge heads (columns). Their structural flexibility makes it extremely difficult to achieve repeated patterns of multicolumn/multilayer polymers and their chiral counterparts in future research. In fact, shortened and condensed multilayer polymers have not been developed, which is probably due to the fact that an efficient catalytic system for their synthesis has not been found. Furthermore, there has not been work on aggregation-induced emission (AIE) of multilayered polymers in the literature thus far, although AIE of other planar/stereopolymers has been documented by Li's team who discovered AIE photophysical phenomenon in 2001 [11].

**Figure 2:**
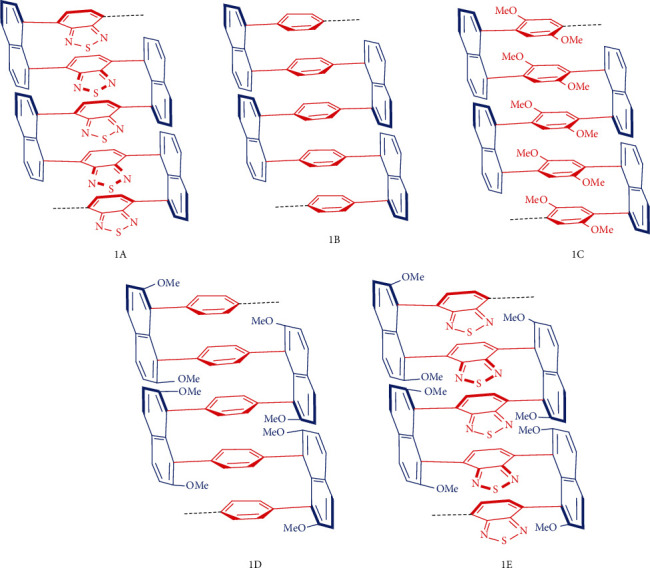
Multilayer 3D polymers in which three columns of parallel layers exist. In the case of 1C, diastereochemistry of bridges has not been determined.

**Figure 3:**
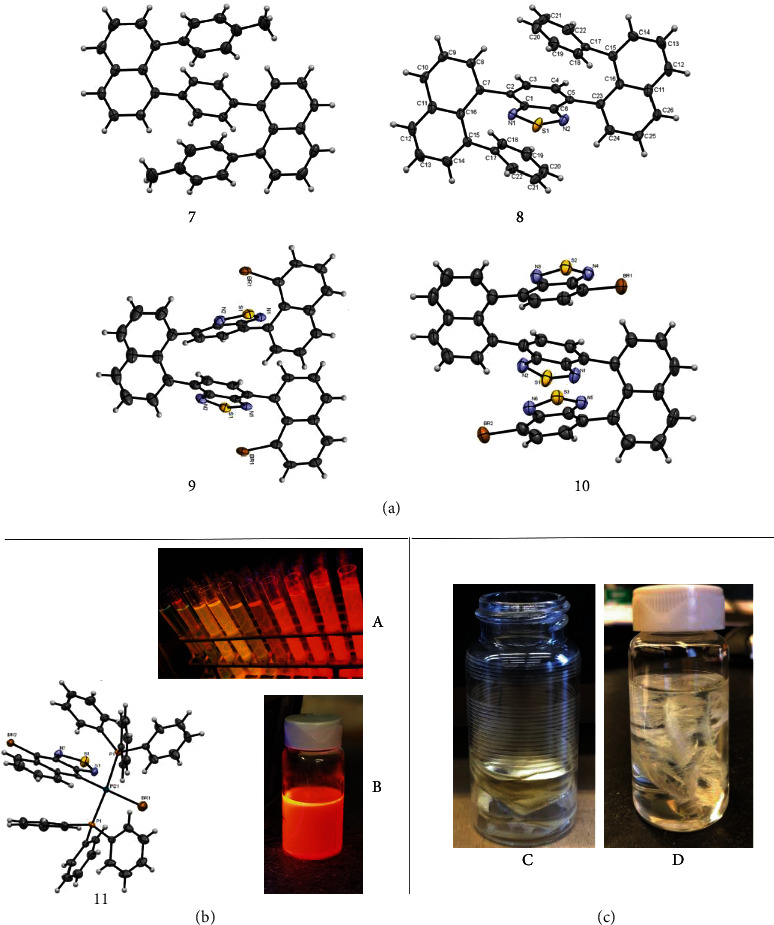
(a) X-ray structures of three-layer building blocks. (b) Crystal structure of a Pd-ligand complex anchored with 4,9-dibromonaphtho[2,3-c][1,2,5]thiadiazole wing and its fluorescence images under UV light (365 nm) in clear solution (A) and condensed muddy mixture (B). (c) Special shapes and pattern of multilayer molecules (C and D).

In this report, we would like to disclose our preliminary results on the design, synthesis, AIE properties, and computational studies of structurally condensed multicolumn and multilayer polymers and their oligomers (Figure 2). New Suzuki-Miyaura catalytic systems were found to be crucial for the synthesis of condensed multilayer 3D polymers in which only one aromatic plane exists between each pair of naphthalene-holding skeletons (Figure 2, 1A–1E). The intramolecularly stacked arrangement of new polymers can be supported by X-ray structural analysis of individual monomers; this would be the first X-ray structures reported on condensed monomers (Figure 3(a)). The unusual intermolecular packing and shaping of these planar compounds are also presented for the first time (Figure 3(c)). It is anticipated that shortened/condensed stereochemical arrangements of planar units within resulting multicolumn/multilayer polymers would make through-space transfers and intermolecular face-to-face packing more efficient. Therefore, the present work would benefit the research and applications of materials with regard to more challenging and desired chemical, electrical, magnetic, and optical properties; aggregation-induced emission (AIE); and other properties in the future.

## 2. Results

### 2.1. Retrosynthetic Analysis (RSA)

**Figure 4:**
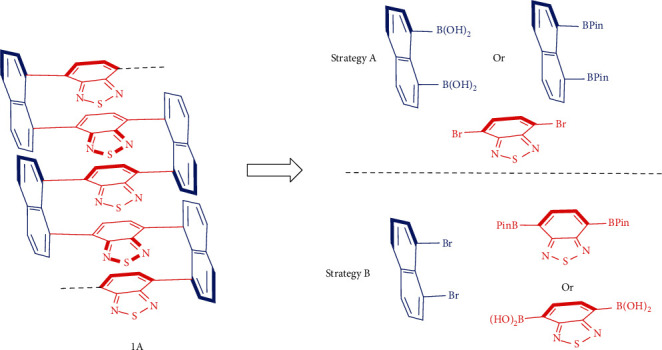
Retrosynthetic analysis (RSA) for multilayer 3D polymers.

Corey's retrosynthetic analysis (RSA) [36] revealed that there are several synthetic strategies to assemble the present multilayer 3D polymers. The assembly takes advantage of the Suzuki-Miyaura C-C coupling under modified catalytic conditions (Figure 4) [37]. The thiadiazole scaffold occupies a special status in polymer science [38], and it also played an important role for the discovery of three-layer 3D chirality [24]. We thus select its copolymer 1A (Figure 4) as a representative for retrosynthetic analysis and polymer synthesis. As described in Figure 4, the first feasible route is to involve synthons of naphthalene-1,8-diyldiboronic acid or 1,8-bis(4,4,5,5-tetramethyl-1,3,2-dioxaborolan-2-yl)naphthalene for coupling with 4,7-dibromobenzo[c][1,2,5]thiadiazole (strategy A). Unfortunately, we have not been able to prepare these two boron-based column synthons. Several other attempts also failed in this synthesis. We then turned our attention to the synthons of 1,8-dibromonaphthalene, 4,7-bis(4,4,5,5-tetramethyl-1,3,2-dioxaborolan-2-yl)benzo[c][1,2,5]thiadiazole, and benzo[c][1,2,5]thiadiazole-4,7-diyldiboronic acid (Figure 4, strategy B). The straightforward one-step conversion of 4,7-bis(4,4,5,5-tetramethyl-1,3,2-dioxaborolan-2-yl)benzo[c][1,2,5]thiadiazole into its boronic acid derivative serves as an extra bonus for this favorable route for the present copolymerization.

### 2.2. Synthesis and Structural Analysis

**Scheme 1:**
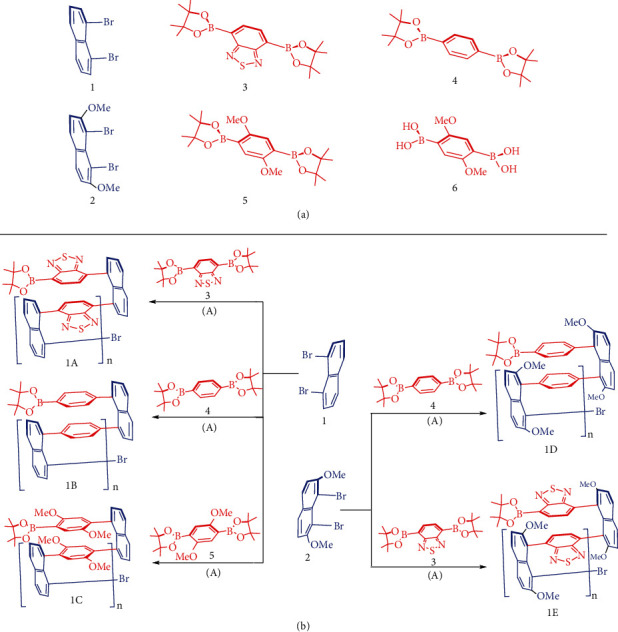
(a) Monomeric building blocks 1-6 for multilayer 3D chiral polymers. (b) Catalytic coupling assembly of multilayer 3D polymers 1A-1E; condition for (A): Pd(PPh_3_)_4_, THF/H_2_O, K_2_CO_3_, 85°C, over 48 hours.

The synthesis of monomers was started from the preparation of 1,8-dibromonaphthalene 1, 1,8-dibromo-2,7-dimethoxynaphthalene 2, 4,7-bis(4,4,5,5-tetramethyl-1,3,2-dioxaborolan-2-yl)benzo[c][1,2,5]thiadiazole 3, 1,4-bis(4,4,5,5-tetramethyl-1,3,2-dioxaborolan-2-yl)benzene 4, 2,2′-(2,5-dimethoxy-1,4-phenylene)bis(4,4,5,5-tetramethyl-1,3,2-dioxaborolane) 5, and (2,5-dimethoxy-1,4-phenylene)diboronic acid 6 (Scheme 1(a)). 1,8-Dibromonaphthalene 1 was synthesized *via* oxidative cyclization by reacting naphthalene-1,8-diamine with sodium nitrite in aqueous media containing acetic acid to give 1H-naphtho[1,8-de][1,2,3]triazine [39, 40]. Ring-opening of the resulting triazine was performed by continuously treating with sodium nitrite and then with a mixture of CuBr in 47% HBr solution to give a yield of 25%.

The thiadiazole bridge building block 3 was obtained through the catalytic carbon-boron coupling of 4,7-dibromo-2,1,3-benzothiadiazole with bis(pinacolato)diboron in cosolvents of CH_2_Cl_2_ and 1,4-dioxane in the presence of (1,1-bis(diphenylphosphino)ferrocene) dichloropalladium (II) as the catalyst and K_2_CO_3_ as an additive [41]. (2,5-Dimethoxy-1,4-phenylene)diboronic acid 6 was obtained via a three-step synthesis [42]. Dibromination of 1,4-dimethoxybenzene gave 1,4-dibromo-2,5-dimethoxybenzene which was converted into the Grignard reagent of magnesium dibromide. The treatment of the resulting Grignard with trimethyl borate was followed by hydrolysis with aqueous H_2_SO_4_ to give 6 in an overall yield of 31%. Unfortunately, the use of (2,5-dimethoxy-1,4-phenylene)diboronic acid 6 for this present polymerization did not give satisfactory results. 2,2′-(2,5-Dimethoxy-1,4-phenylene)bis(4,4,5,5-tetramethyl-1,3,2-dioxaborolane) 5 was then employed as a replacement. The synthesis of the latter was started from dibromination of 1,4-dimethoxybenzene followed by treating with 2-isopropoxy-4,4,5,5-tetramethyl-1,3,2-dioxaborolane to give an overall yield of 25% [43].

Previous aromatic ring-layered polymers can be synthesized under modified Suzuki-Miyaura catalytic coupling requiring relatively short polymerization periods (48 h) [10, 44]. This was attributed to the fact that the corresponding monomers have either longer bridges, such as more than one aromatic ring and an aromatic ring anchored with carbon-carbon triple bond, or larger columns, such as 2,7-ditert-butyl-4,5-diiodo-9,9-dimethylxanthene and 1,4-diiodo-2,5-bis(nonyloxy)benzene. In our cases, five to seven days are spent to generate multilayer 3D polymers and to cap their ends for proton NMR analysis.

For the synthesis of polymers, we choose polymer 1C in Scheme 1 as a representative for description. Multiple Suzuki-Miyaura C-C couplings were conducted by slightly modifying the classical Suzuki-Miyaura coupling system. A ratio of 0.87 : 1 of 1,8-dibromonaphthalene to 2,2′-(2,5-dimethoxy-1,4-phenylene)bis(4,4,5,5-tetramethyl-1,3,2-dioxaborolane) was mixed in THF/H_2_O (5 : 1, *v*/*v*) in the presence of Pd(PPh_3_)_4_ (5% mol) as catalyst and potassium carbonate (3.0 equiv) as an additive. The reaction was stirred at 85°C for over 48 h with monomeric starting materials being consumed. However, this condition resulted in the mixture being contaminated with some oligomers showing broad peaks at chemical shift around 1.0 in the ^1^H-NMR spectrum. We then changed the ratios of 1,8-dibromonaphthalene to 2,2′-(2,5-dimethoxy-1,4-phenylene)bis(4,4,5,5-tetramethyl-1,3,2-dioxaborolane) to 1 : 1 and 1.1 : 1. and conducted the reaction over 48 hours. The quality of polymeric products in the latter case was substantially improved for the case of the 1.1 : 1 ratio and gave a chemical yield of 38.9%. The crude products were washed with methanol several times and contained nearly no small oligomers as revealed by MeO- signal in ^1^H-NMR analysis with DMSO-*d*_6_ as solvent.

**Figure 5:**
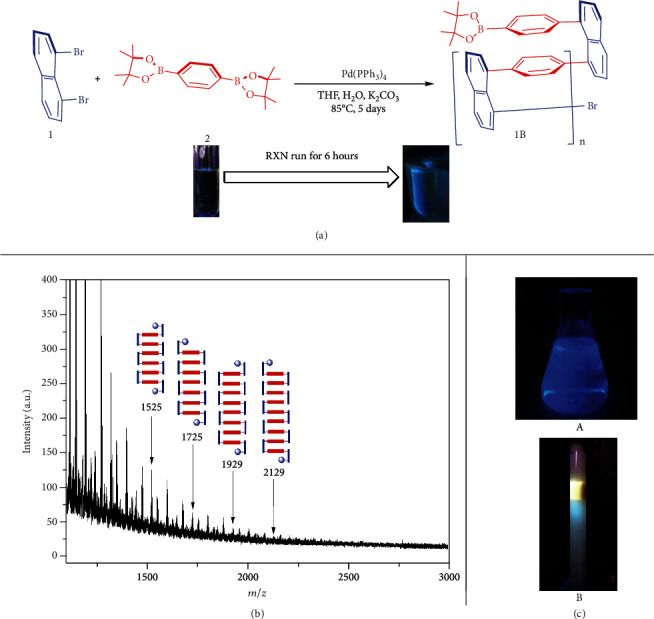
(a) Color changing during copolymerization for 1B. (b) MALDI-TOF mass spectrum of the chloroform-soluble part of polymer. (c) Fluorescence images of oligomers under UV light (365 nm) in chloroform (A) and silica gel column (B).

For this set of catalytic polymerizations, the reaction mixture is colorless at the beginning as shown in Figure 5(a). A green color appeared after the reaction was performed for about 6 hours while being heated/stirred at 85°C when irradiated under UV. This color became darker as the reaction continues occurring, which indicates the formation of polymers/oligomers. At the same time, more solids were accumulated during the polymerization progress. The MALDI-TOF mass spectrum revealed that a series of multilayer oligomers, such as six to nine layers of oligomers, are formed (Figure 5(b)). Very small amounts of these oligomers remained at the end of reaction and showed dark blue in the mother liquor solution (Figure 5(c), A).

It is extremely difficult to separate individual oligomers *via* column chromatography in which these compounds also display a light blue color, and a small amount of polymer stuck on top of the silica gel showed a gold yellow color (Figure 5(c), B). We attempted recrystallization of any of these relatively higher oligomers, but failed. However, we successfully obtained several X-ray structures of similar three-layer compounds (Figure 3(a)), which can directly confirm the multiple-layered higher oligomers and polymers.

The compound 7 (Figure 3(a)) was obtained by reacting 1,4-bis(4,4,5,5-tetramethyl-1,3,2-dioxaborolan-2-yl)benzene (1.0 equiv) and 1,8-dibromonaphthalene (2.2 equiv) in the presence of Pd(PPh_3_)_4_ followed by another coupling with *p*-tolylboronic acid. Compound 8 (Figure 3(a)) was synthesized through a similar route to that of 7. Compound 9 was generated by reacting 4,7-bis(4,4,5,5-tetramethyl-1,3,2-dioxaborolan-2-yl)benzo[c][1,2,5]thiadiazole (1.0 equiv) and 1,8-dibromonaphthalene (1.1 equiv) in the presence of Pd(PPh_3_)_4_. Compound 10 was obtained through a six-step synthesis involving two key couplings: (a) the coupling of 4,7-bis(8-bromonaphthalen-1-yl)benzo[c][1,2,5]thiadiazole with (BPin)_2_ to afford 4,7-bis(8-(4,4,5,5-tetramethyl-1,3,2-dioxaborolan-2-yl)naphthalen-1-yl)benzo[c][1,2,5]thiadiazole, and (b) the coupling of the above benzo[c][1,2,5]thiadiazole with 4,7-dibromobenzo[c][1,2,5]thiadiazole. To the best of our knowledge, these are among the first X-ray structures of condensed multilayer oligomers.

As shown in X-ray structural analysis for 10, both the decked structure and the multilayer diastereochemistry is confirmed. For the latter, the sulfur heads of thiadiazole are arranged in antidirections alternatively. This indicates that multilayer polymers resulting from 3 have diastereochemistry of anti-anti or head-tail arrangements, i.e., thiophene rings of bridges are alternatively oriented. There are no other diastereoisomers formed during coupling reaction indicating that the layer diastereoselectivity is over 95% (essentially, only one diastereoisomer was generated). It is the first time to propose a concept of multilayer or layered diastereoselectivity and stereochemistry in this work.

We have been trying to isolate the key intermediates during the formation of the above oligomers, oligomer-type of compounds, and polymers, but we failed. However, during the similar synthesis of another three-layer oligomer using 4,9-dibromonaphtho[2,3-c][1,2,5]thiadiazole, we successfully obtained crystals of a Pd-ligand complex anchored with 4,9-dibromonaphtho[2,3-c][1,2,5]thiadiazole wing 11 (Figure 3(b)). The X-ray structural analysis revealed that the layers of two phenyl rings of phosphine ligands and the thiadiazole ring are arranged nearly in a parallel manner. This complex turned out to be very stable at room temperature in the air. It can be purified *via* column chromatography conveniently. As shown in Figure 3(b), both its clear solution (Figure 3(b), A) and condensed muddy mixture (Figure 3(b), B) display a shining brown color under UV irradiation.

Interestingly, the trimeric compound 10 in Figure 3(a) formed a pattern of regularly arranged loops on the surface of a vial when its solution was slowly evaporated at room temperature within 1-2 weeks (Figure 3(c), C). This phenomenon indicates that decked packing fashion occurs not only in intramolecular but also in an intermolecular manner. The planar structural arrangement would be the major reason forming these loops. In the meanwhile, another interesting phenomenon came out with a similar precursor, 4-bromo-7-(8-(7-(8-(pin)-naphthalen-1-yl)benzo[c][1,2,5]thiadiazol-4-yl)naphthalen-1-yl)benzo[c][1,2,5]thiadiazole, which formed feather types of leaves even in solution (Figure 3(c), D). To the best of our knowledge, these are the first chemical leaves which can be directly seen by naked eyes.

**Figure 6:**
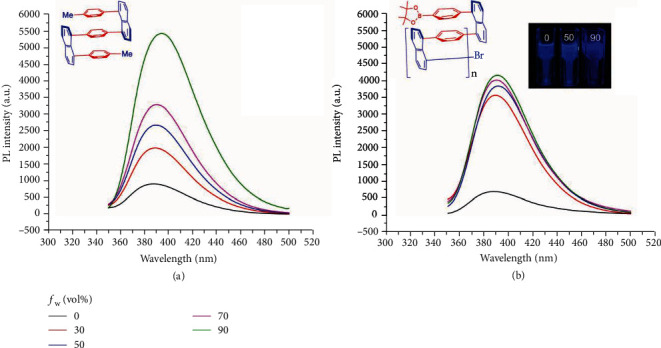
Photoluminescence (PL) spectra of 7 (a) and 1B (b) in THF/water mixtures with different water fractions (*f*_w_); *c*_12_ = 1 *μ*M, *c*_1B_ = 4 *μ*g/mL; excitation wavelength (*λ*_ex_): 308 nm. Inset: fluorescence photographs of 1B in the THF/water system.

To demonstrate the activities of aggregation-induced emission (AIE)/aggregation-enhanced emission (AEE) quantitatively, the photoluminescence study of a monomer-type of trimer-partial 7 and the closely relevant polymer 1B was conducted (Figure 6). Dilute solution of 7 in THF shows relatively low fluorescence intensity at ~390 nm but exhibits gradual emission enhancement with increasing water fraction in the THF/water mixture. The X-ray structure offers a clue to the explanation that the rotation of phenyl rings mostly dissipates the excited-state energy in a low radiative manner. By restricting the intramolecular rotations (RIR) through aggregation formation by increasing the fraction of less soluble solvent (water in this case), the luminescence becomes much more intense.

The PL spectra of the multilayer polymer with the same skeleton 1B manifest AIE/AEE property as well. When the water fraction was increased to 30%, the solution of the columnar-shaped polymer was nearly saturated. The intermolecular aggregation largely suppresses the rotational motions of phenyl rings so that the exciton energy cannot be depleted by the radiation-less decay, thus making the molecule emissive jump almost 7-fold. However, the intramolecular arrangement becomes more regular and compacted, which leads to the through-space *π*-*π* stacking interaction. This energy consumption offsets partial AIE/AEE effect, which is revealed by slowly increasing PL intensity from *f*_w_ = 30% to 90%. This result coincides with the qualitative experiment shown in the inserted photo (Figure 6(b)). Based on the collected data, the fluorescence lifetimes *τ*_f_ for both 7 and 1B with different water fractions were fitted as shown in Table S26. The lifetime *τ*_f_ of trimer 7 clearly increases with higher water fractions from 45.66194 ns to 57.81773 ns, while there is no obvious trend for the polymer 1B.

### 2.3. Computational Investigations

**Figure 7:**
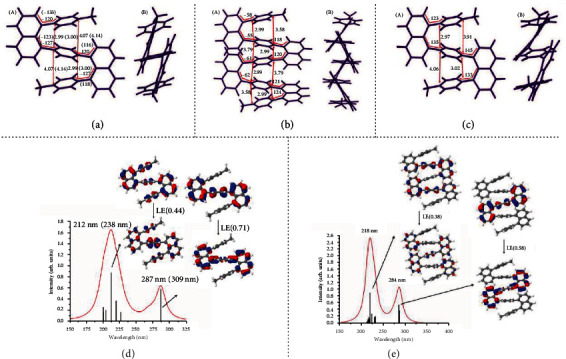
Selected geometry data for ground state trimer 7 (a), ground state pentamer (b), and S_1_ state optimized trimer 7 (c) structure: (A) front view and (B) side view. Corresponding X-ray data are shown for comparison in parentheses. Distances are given in Å and angles in degrees. UV spectrum and characterization for trimer (d) and pentamer (e) by means of NTOs (fractional occupation in parentheses); wavenumbers in parentheses show experimental results; all calculations are using the *ω*B97XD/SVP method in the gas phase.

Computational studies on the trimer 7 and the related pentamer (Figure 7) have been performed to analyze especially the character of the electronic transitions. Based on previous investigations on similar stacked systems (Figure 1(b)) [26] and other stacked PAHs such as pyrene dimers [27], calculations have been performed using two different types of methods: (i) time-dependent density functional theory (TD-DFT) using the *ω*B97XD range-separated functional and (ii) the *ab initio* second-order algebraic diagrammatic construction method (ADC(2)). More details about the methods used can be found in the SI, Section 5. Both methods give similar results. Thus, we will concentrate mostly on the presentation of the DFT data. All other results can be found in the SI as well.

The optimized geometry of trimer 7 in the ground state is displayed in Figure 7(a), A, for the gas phase using the *ω*B97XD/SVP method. Comparison of selected bond distances and angles with corresponding X-ray data shows good agreement. The overall zig-zag orientation of naphthalene and benzene rings (Figure 7(a), B) agrees well with the experimental structure also. The structural data computed with the same method in THF solution and by the SOS-MP2/SV(P) approach in the gas phase (Figure S30 and Figure S31) indicate only small effects induced by the solvent and by the different computational method, respectively. The computed structure of the pentamer is displayed in Figure 7(b). It shows a straightforward extension of the zig-zag arrangement of the trimer. The shorter of the two interring CC distances of 2.99 Å remains practically the same as in the trimer whereas the longer one is reduced from 4.07 Å (trimer) to 3.6-3.8 Å.

The calculated UV spectrum for the trimer is displayed in Figure 7(d) using the *ω*B97XD/SVP method in the gas phase. Corresponding spectra obtained with *ω*B97XD/SVP for THF solution (Figure S32) and ADC(2)/SV(P) for the gas phase (Figure S33) confirm these results. Quite good agreement with the experiment is observed in terms of the overall shape and the location of the band maxima of the two experimental band maxima (Figure S36); they are only shifted somewhat to higher energies. The analysis of the natural transition orbitals (NTOs) shows a combination of local excitations (LE) with significant contribution of charge transfer of ~0.5 *e* from the benzene ring to the naphthalene rings in case of S_1_. Only the central benzene ring is involved in the S_1_ transition. It should also be noted that especially the high-energy band is not dominated by one electron/hole pair transition alone. The spectrum of the pentamer resembles closely the trimer, as shown in Figure 7(e).

**Figure 8:**
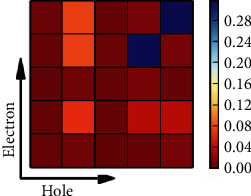
Omega matrix for the trimer 7 using the *ω*B97XD/SVP method and analyzing the first excited state. Segment nos. 1-3 represent the three benzene rings (seg. 2 is the middle one) and 4-5 number the two naphthalene rings.

The omega matrix (Equation S1) displayed in Figure 8 for trimer 7 is based on the transition density from the ground state to S_1_ and is used to visualize the localization of the transition in the different parts of the molecule. Individual diagonal blocks indicate local excitations, and multiple diagonal contributions signal the exciton character. Off-diagonal blocks denote CT between respective blocks. Five segments have been defined: segments 1 to 3 represent the three benzene rings and segments 4 to 5 indicate the two naphthalene units. For more information on the construction of this matrix, see the SI. The two blue squares in the diagonal of the picture demonstrate excitonic character in the two naphthalene units. This is the dominant contribution. Another weaker diagonal block shows participation of the center benzene ring. As already discussed above, this benzene ring is also involved in CT with the naphthalene moieties. The other segments show vanishing contributions. The location of the excitation on the exterior naphthalene rings is of interest in view of the AIE phenomenon discussed above and on possible mutual intermolecular interactions. However, more investigations on aggregated systems have to be performed in order to allow more mechanistic insight into the consequences of the aggregation on the electronic transitions.

To discuss the fluorescence emission process, the geometry of the S_1_ state has been optimized since the emission process starts from S_1_ according to Kasha's rule [45–47]. The S_1_ optimization does not change the overall shape of the trimer structure as a comparison between the structures in Figure 7(a) (ground state) and Figure 7(c) (S_1_ state) shows. The structural changes affect primarily the inner benzene ring, which follows a quinoid pattern, and the naphthalene rings. The CC bonds between the central benzene ring and the naphthalene rings are reduced somewhat as well. Changes in bond distances amount to a few hundredths of an Å and can be rationalized by the NTO excitation pattern displayed in Figure 7(d) for the S_1_ state. The optimized S_1_ structure of the trimer using the ADC(2)/SV(P) method follows a similar behavior. The S_1_ energy is decreased by 0.46 eV and 0.76 eV for the trimer using the *ω*B97XD/SVP and ADC(2)/SV(P) methods, respectively (compare vertical and adiabatic energies in Table 1). The Stokes shift computed as the difference of vertical absorption and emission is pronounced and amounts to 0.99 eV (85 nm) for *ω*B97XD/SVP and 1.15 eV (108 nm) for ADC(2)/SV(P). Table 1 shows also very good agreement of the *ω*B97XD calculations using the more flexible TZVP basis in comparison with the SVP results. The optimized structure for the S_1_ state of the pentamer using the *ω*B97XD/SVP method is shown in Figure S29. It follows the same structural changes as the trimer structure for S_1_ (Figure 7(c)). Also, the adiabatic transition energy and the Stokes shift (Table 1) are almost identical to the trimer values.

Last but not least, the polymerization of 4,7-bis(8-bromonaphthalen-1-yl)benzo[c][1,2,5]thiadiazole with 4,7-bis(4,4,5,5-tetramethyl-1,3,2-dioxaborolan-2-yl)benzo[c][1,2,5]thiadiazole under the above standard catalytic conditions has been proven to be successful. This system resulted in a chemical yield of 74% with Mn and Mw as 10,149 and 14,533, respectively (SI, Table S36). This strategy makes it possible to synthesize those multilayer 3D polymers with differentiated aromatic bridges instead of the same ones as shown in Scheme 1. In addition, another new two columns of multilayer 3D polymers have also been successfully obtained by using 2-(8-bromonaphthalen-1-yl)-4,4,5,5-tetramethyl-1,3,2-dioxaborolane as the sole monomer (SI, Figure S36).

## 3. Discussion

In conclusion, we present here a new class of structurally condensed triple-column/multilayer 3D polymers and oligomers. The key element of this intramolecularly stacked arrangement is characterized by only one aromatic plane which exists between each pair of naphthalene holding skeletons, which could be deduced from X-ray structural analysis of distinct monomers. The results determined by GPC, ^1^H-NMR, and MALDI-TOF analysis are convincing, supporting that the new Suzuki-Miyaura catalytic systems are significant for this effective polymerization. Preliminary DLS and TEM results of the particle size study are shown in the supporting information. Both multilayer 3D monomers and polymers in this work exhibit fluorescence activity and aggregation-induced emission (AIE) property under specific wavelength irradiation. Computational work has been conducted on geometries for ground states of the trimer 7 (A) and the pentamer (B) and the S_1_ state of the trimer 7 (C). We found that the UV/vis spectrum predicted by the two computational methods used is well consistent with the experimental data. The S_1_ transitions can be characterized as a combination of mostly local character combined with nonnegligible CT contributions. The most interesting outcome of the theoretical analysis is the finding that the S_1_ transition is located mostly on the naphthalene units, which could indicate sensitivity of this transition to the aggregation process.

## Figures and Tables

**Table 1:** Vertical absorption (eV/nm), adiabatic (eV/nm), vertical emission energies (eV/nm), and Stokes shifts (eV/nm) for the S_1_ state of trimer 7 and pentamer in gas phase.

System	Method	Vertical absorption	Adiabatic transition	Vertical emission	Stokes shift^a^
Trimer	*ω*B97XD/SVP	4.32/287	3.86/321	3.33/372	0.99/85
	ADC(2)/SV(P)	4.26/291	3.50/354	3.11/399	1.15/108
	*ω*B97XD/TZVP	4.35/285	3.86/321	3.27/379	1.08/94
Pentamer	*ω*B97XD/SVP	4.29/289	3.85/322	3.36/369	0.93/80

^a^Exp. value for trimer 91 nm.

## Supplementary Materials

Supplementary Materials Figures S1-S9 ^1^H NMR of the synthesized compounds 1-6 and 12. Figures S10-S21: ^1^H NMR and GPC data of 1A-1F. Figure S22 and S23: DLS size distributions curves of 7 and 1B. Figure S24: TEM images of 7 and 1B. Figure S25 and Tables S1-S5: crystallographic data for 7. Figure S26 and Tables S6-S10: crystallographic data for 8. Figure S27 and Tables S11-S15: crystallographic data for 9. Figure S28 and Tables S16-S20: crystallographic data for 10. Figure S29 and Tables S21-S25: crystallographic data for 11. Figure S30: selected geometry data for the trimer structure optimized for the ground state using the *ω*B97XD/SVP method in THF: (a) front view and (b) side view. Distances are given in Å and angles in degrees. Figure S31: selected geometry data for the trimer structure optimized for the ground state using the SOS-MP2/SV(P) method in the gas phase: (a) front view and (b) side view. Distances are given in Å and angles in degrees. Figure S32: UV spectrum and characterization of most important transitions for the trimer by means of NTOs (occupation fractions in parentheses) using the *ω*B97XD/SVP method in THF. Figure S33: UV spectrum and characterization of most important transitions for the trimer by means of NTOs (occupation fractions in parentheses) using the ADC(2)/SV(P) method in the gas phase. Figure S34: selected geometry data for the trimer structure optimized for the S1 state using the ADC(2)/SV(P) method in the gas phase: (a) front view and (b) side view. Distances are given in Å and angles in degrees. Figure S35: selected geometry data for the pentamer structure optimized for the S1 state using the *ω*B97XD/SVP method in the gas phase: (a) front view and (b) side view. Distances are given in Å and angles in degrees. Figure S36: UV-vis spectrum of trimer 7.
