# Supplementary material for: Triple-Columned and Multiple-Layered 3D Polymers: Design, Synthesis, Aggregation-Induced Emission (AIE), and Computational Study
Source: Research (Wash D C). 2021 Feb 8;2021:3565791. doi: 10.34133/2021/3565791 (PMC7888304; doi:10.34133/2021/3565791)
Supplement: Supplementary Materials — Figures S1-S9 1H NMR of the synthesized compounds 1-6 and 12. Figures S10-S21: 1H NMR and GPC data of 1A-1F. Figure S22 and S23: DLS size distributions curves of 7 and 1B. Figure S24: TEM images of 7 and 1B. Figure S25 and Tables S1-S5: crystallographic data for 7. Figure S26 and Tables S6-S10: crystallographic data for 8. Figure S27 and Tables S11-S15: crystallographic data for 9. Figure S28 and Tables S16-S20: crystallographic data for 10. Figure S29 and Tables S21-S25: crystallographic data for 11. Figure S30: selected geometry data for the trimer structure optimized for the ground state using the ωB97XD/SVP method in THF: (a) front view and (b) side view. Distances are given in Å and angles in degrees. Figure S31: selected geometry data for the trimer structure optimized for the ground state using the SOS-MP2/SV(P) method in the gas phase: (a) front view and (b) side view. Distances are given in Å and angles in degrees. Figure S32: UV spectrum and characterization of most important transitions for the trimer by means of NTOs (occupation fractions in parentheses) using the ωB97XD/SVP method in THF. Figure S33: UV spectrum and characterization of most important transitions for the trimer by means of NTOs (occupation fractions in parentheses) using the ADC(2)/SV(P) method in the gas phase. Figure S34: selected geometry data for the trimer structure optimized for the S1 state using the ADC(2)/SV(P) method in the gas phase: (a) front view and (b) side view. Distances are given in Å and angles in degrees. Figure S35: selected geometry data for the pentamer structure optimized for the S1 state using the ωB97XD/SVP method in the gas phase: (a) front view and (b) side view. Distances are given in Å and angles in degrees. Figure S36: UV-vis spectrum of trimer 7. [file 3565791.f1.pdf]

Supporting Information for

Triply Columned and Multiply Layered *3D* Polymers: Design, Synthesis, Aggregation-Induced Emission (AIE) and Computational Study

Guanzhao Wu,[1,2,†] Yangxue Liu,[1,†] Zhen Yang,[2,†] Liulei Ma,[1] Yao Tang,[1] Hossein Rouh[1] Qixuan Zheng,[1] Peng Zhou,[2] Jia-Yin Wang,[2] Farhan Siddique,[4] Sai Zhang,[1] Daniel Unruh,[1] Adelia J. A. Aquino,[3,4,]* Hans Lischka,[3,4,]* Kristin M. Hutchins[1,]* and Guigen Li[1,2,]*

[1] *Department of Chemistry and Biochemistry, Texas Tech University, Lubbock, Texas 79409-1061, USA.*
[2] *Institute of Chemistry and BioMedical Sciences, School of Chemistry and Chemical Engineering, Nanjing University, Nanjing, 210093, China.*
[3] *Department of Mechanical Engineering, Texas Tech University, Lubbock, TX, 79409, USA*
[4] *School of Pharmaceutical Sciences and Technology, Tianjin University, Tianjin 300072, P.R. China.*

[*]Correspondence should be addressed to Guigen Li; guigen.li@ttu.edu, Kristin M. Hutchins; kristin.hutchins@ttu.edu, Hans Lischka; hans.lischka@univie.ac.at, and Adelia J. A. Aquino; adelia.aquino@ttu.edu
[†]These authors contributed equally to this work.

Table of Contents

1. **General Information S2**
2. **Synthetic Procedures S3**
3. **NMR Spectrums and GPC data S8**
4. **X-ray Data S28**
5. **Computational details and spectrums S88**
6. **References S108**

1. General Information

Unless otherwise stated, all reactions were magnetically stirred and conducted in oven-dried glassware in anhydrous solvents under Ar. Solvents and liquid reagents, as well as solutions of solid or liquid reagents were added via syringes, stainless steel or polyethylene cannulas through rubber septa or through a weak Ar counter-flow. Cooling baths were prepared in Dewar vessels, filled with ice/water (0 °C) or dry ice/acetone (-78 °C). Heated oil baths were used for reactions requiring elevated temperatures. Solvents were removed under reduced pressure at 40-65 °C using a rotavapor. All given yields are isolated yields of chromatographically and NMR spectroscopically materials.

All commercially available chemicals were used as received without further purification. Solvents as follows: CH_3OH, toluene, EA, ether, DCM, dioxane, acetone were used without further purification. THF and DCM are delivered from an Innovation Technology solvent system.

The ^1H and ^{13}C NMR spectra were recorded in $CDCl_3$ or DMSO-d_6 on 400 MHz and 500 MHz instruments with TMS as internal standard. For referencing of the ^1H NMR spectra, the residual solvent signal (δ = 7.26 for $CDCl_3$ and δ = 2.50 for DMSO-d_6) were used. In the case of the ^{13}C NMR spectra, the signal of solvents (δ = 7.16 for $CDCl_3$ and δ = 39.52 for DMSO-d_6) were used. Chemical shifts(δ) were reported in ppm with respect to TMS. Data are represented as follows: chemical shift, multiplicity (s = singlet, d = doublet, t = triplet, m = multiplet), coupling constant (*J*, Hz), and integration. MALDI-TOF analyses were carried out using an ABI/MDS SCIEX 4800 Mass Spectrometer. Samples **7** and **1B** were characterized by DLS (Zetasizer APS) in methanol. Samples solution in Toluene were dropped on TEM grid substrates and the drying droplets were then inspected by TEM (H7650) at an accelerating voltage of 80 kV and 60 kV for monomer and polymer, respectively. X-Ray crystallographic analysis was performed with a SMART CCD and a P4 diffractometer. X-ray data were collected on a Rigaku XtaLAB Synergy-*i* Kappa diffractometer equipped with a PhotonJet-*i* X-ray source operated at 50 W (50kV, 1 mA) to generate Cu Kα radiation (λ = 1.54178 Å) and a HyPix-6000HE HPC detector. GPC data were collected using TOSOH EcoSEC HLC-8320 GPC equipped with a dual-flow refractive index detector. A UV detector is also included for UV visible polymers and can be used in tandem with the RI detector. The installed columns have a range of 500 – 10^7 Da. Samples were run for 30 minutes with flow rate 1.001 mL/min. Polystyrene (PS) standards were used for calibration in our experiments.

2. Synthetic Procedures

1 was synthesized from 1,8-diaminonaphthalene following the reported procedure [1] with very minor modification.

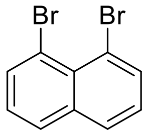

1,8-dibromonaphthalene (1): white solid, 2.3 g, 25% yield. 1H NMR (400 MHz, CHLOROFORM-D) δ 7.94 – 7.89 (m, 2H), 7.82 – 7.76 (m, 2H), 7.28 – 7.21 (m, 2H). MS(ESI): m/z, 285.2 [M+H]+.

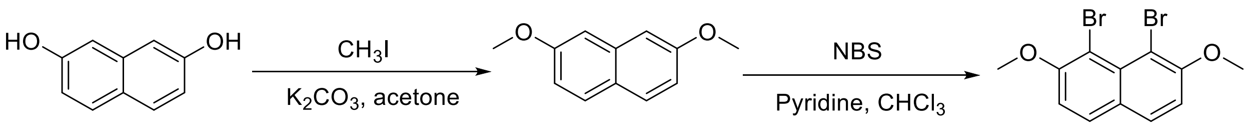

2-1 2-2 2

2,7-dimethoxynaphthalene (2-2)

2,7-dihydroxynaphtalene (8.0g, 50 mmol) was dissolved in 100 mL acetone. Then 34.2 g of powdered potassium carbonate and methyl iodide (18.7mL, 0.3mol) was added dropwise and the mixture was stirred under Argon at 0 ºC. Then the reaction mixture was slowly warmed to r.t. and 20 mL 1M NH_4OH was added with stirring and the acetone was evaporated, the colorless deposit was filtered. The solid was recrystallized in methanol and dried in air, giving a white solid (8.0g, 85% yield).^{1}H NMR ($CDCl_3$, 400 M HZ) δ: 7.64(dd, *J*=8.8, 9.2 Hz, 2H), 7.04(s, 2H), 6.98 (m, 2H), 3.90 (s, 6H). MS(ESI): m/z, 189.1 $[M+H]^+$.

1,8-dibromo-2,7-dimethoxynaphthalene (2)

NBS (8.18 g, 46.0 mmol) and 2.4 mL pyridine were dissolved in 70 mL of chloroform and the solution refluxed under argon for 1 hour. A solution of 2,7-dimethoxy naphthalene (2.16 g, 11.5 mmol) in 12.5 mL chloroform was added dropwise and refluxing continue for 9 hours. The mixture was cooled to r.t. and silica gel was added and product separated by recrystallizing in methanol, giving brown solid (2.79 g, 70% yield).^{1}H NMR ($CDCl_3$, 400 M HZ) δ: 7.73(dd, *J*=8.8, 8.8 Hz, 2H), 7.15(dd, *J*=9.2, 9.2 Hz 2H), 4.0 (s, 6H). MS(ESI): m/z, 345.6 $[M+H]^+$.

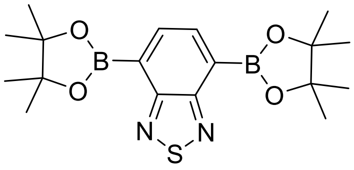

4,7-bis(4,4,5,5-tetramethyl-1,3,2-dioxaborolan-2-yl)benzo[*c*][1,2,5]thiadiazole (3): **3** was synthesized from 4,7-Dibromo-2,1,3-benzothiadiazole following the reported procedure [2] with very minor modification. Yellow solid, 82% yield. ^{1}H NMR (400 MHz, CHLOROFORM-D) δ 8.11 (d, *J* = 2.4 Hz, 2H), 1.43 (dd, *J* = 2.3 Hz, 24H). MS(ESI): m/z, 389.2 $[M+H]^+$.

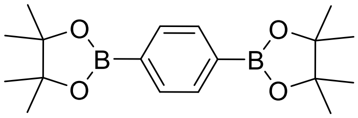

1,4-bis(4,4,5,5-tetramethyl-1,3,2-dioxaborolan-2-yl)benzene (4): white solid, 66% yield. ^{1}H NMR (400 MHz, CHLOROFORM-D) δ 7.78 (s, 4H), 1.33 (s, 24H). MS(ESI): m/z, 331.2 $[M+H]^+$.

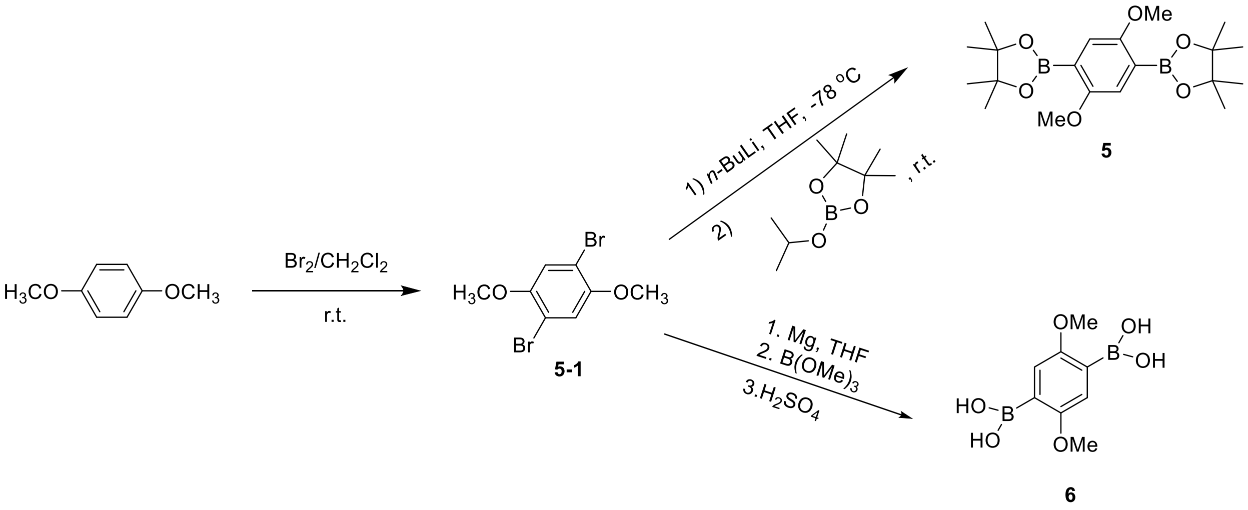

1,4-dibromo-2,5-dimethoxybenzene (5-1)

1,4-dimethoxybenzene (0.6 g, 4.3 mmol) was placed in 100-mL round bottle flask, and CH_2Cl_2 (30 mL) was added. A bromine solution (0.54 mL, 10.5 mmol) was added through dropping funnel and the mixture was stirred at room temperature overnight and quenched with an excess amount of 1M KOH. The reaction mixture was diluted with CH_2Cl_2 (30 mL) and washed with water (2 × 30 mL). The organic phase was separated and concentrated to dryness under reduced pressure. The residue was purified by recrystallization with EtOH, giving a white solid of **5-1** (0.8g, 62% yield). ^{1}H NMR (400 MHz, CHLOROFORM-D) δ 7.09 (s, 2H), 3.83 (s, 6H). MS (ESI): m/z, 295.5 $[M+H]^+$.

2,2'-(2,5-dimethoxy-1,4-phenylene)bis(4,4,5,5-tetramethyl-1,3,2-dioxaborolane) (5)

5-1 (1.5 g, 5.0 mmol) was placed in a 100-mL two-neck bottle and vacuum purged and filled with argon. Anhydrous THF (30 mL) was added, and the mixture was stirred at -78 °C for 30 minutes. 2.5 M *n*-butyl lithium (5.1 mL, 12.7 mmol) was slowly added at a rate of 0.1 mL/sec. The mixture was stirred at -78 ^{0}C for 30 minutes, then 2-isopropoxy-4,4,5,5-tetramethyl-1,3,2-dioxaborolane (4.2mL, 20.5 mmol) was added at a rate of 0.5 mL/sec. The reaction was slowly warmed to room temperature and stirred overnight. The reaction mixture was diluted with EtOAc (100 mL) and washed with water (3 × 100 mL). The organic phase was separated and concentrated to dryness under reduced pressure. The residue was purified by recrystallization with hexane, giving a white solid of **5** (0.8 g, 40% yield). ^{1}H NMR (400 MHz, CHLOROFORM-D) δ 7.14 (s, 2H), 3.81 (s, 6H), 1.34 (s, 24H). MS(ESI): m/z, 391.2 $[M+H]^+$.

(2,5-dimethoxy-1,4-phenylene)diboronic acid (6)

Dibromo dimethoxy benzene (2.0 g, 6.757 mmol) was added to the mixture of Magnesium powder (0.657 g, 27 mmol) and 35 mL dry THF. The reaction mixture was refluxed for 2 hours and cooled to -78 °C and 3.01 mL of $(MeO)_3B$ was added dropwise and the reaction was kept at room temperature for 12 hours. After that 70 ml of 2M H_2SO_4 was added and the reaction was stirred for 3 hours. The organic layer was separated and aqueous layer was extracted with ethyl acetate. All organic layer was washed with water and dried over magnesium sulfate. The solvent evaporated and the residue was recrystallized from water/acetonitrile mixture to afford a white solid, 50% yield. ^{1}H NMR (DMSO-*d*6): 3.77 (6H, s), 7.16 (2H, s), 7.80 (4H, s). MS(ESI): m/z, 227.8 $[M+H]^+$.

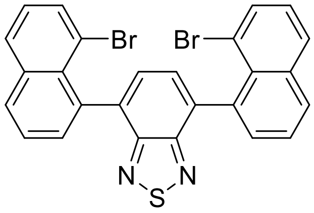

4,7-bis(8-bromonaphthalen-1-yl)benzo[c][1,2,5]thiadiazole (12)

To a round bottom flask, dissolved **3** (1.0 mmol), 1,8-dibromonaphthalene (2.3 mmol), K_2CO_3 (6.0 mmol) into THF/H_2O (30 ml/6 ml). After three vacuum/Ar cycles to replace the air inside with argon, $Pd(PPh_3)_4$ (0.2 mmol) was added to the flask. Heated the resulting mixture to 85°C and stirred for 12 h. Reaction was monitored by TLC analysis and worked up for column (hexane/DCM, 10/1 to 5/1) to afford green yellow solid, 58% yield. 1H NMR (400 MHz, CHLOROFORM-D) δ 8.04 – 7.99 (m, 2H), 7.96 (dd, J = 8.2, 1.0 Hz, 2H), 7.79 – 7.57 (m, 8H), 7.34 (dd, J = 15.2, 7.3 Hz, 2H). MS(ESI): m/z, 546.9 $[M+H]^+$.

General Procedure for **polymer 1A – 1F**

Take Polymer **1B** as an example for synthesis.

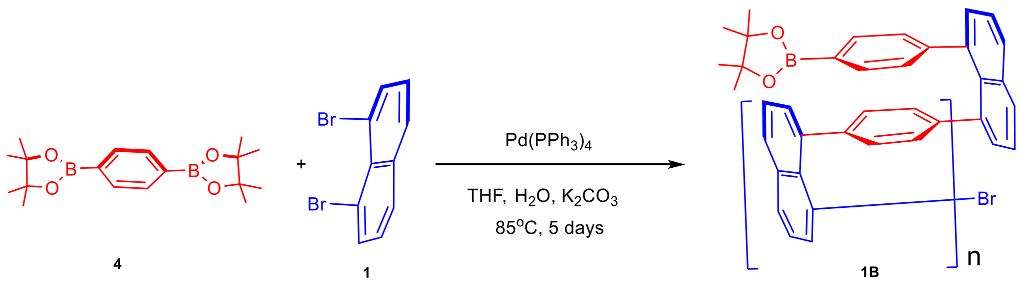

An oven dried 50 mL schlenk flask was kept under argon, then 1,4-bis(4,4,5,5-tetramethyl-1,3,2-dioxaborolan-2-yl)benzene (165.0 mg, 0.5 mmol, 1 equiv), 1,8-dibromonaphthalene (156.2 mg, 0.55 mmol, 1.1 equiv), $Pd(PPh_3)_4$ (28.9 mg, 0.025 mmol, 0.05 eq) and K_2CO_3 (207.0mg, 1.5 mmol, 3 equiv) were added. 15 mL THF and 3 ml H_2O were added to the Schlenk flask. The Schlenk flask was degassed under vacuum and refilled with argon. It was then heated at 85 ^{o}C for over 48 hours. The mixture was cooled to room temperature. Finally, the resulting mixture was poured into MeOH/HCl in one-pot. The precipitated materials were recovered by filtration through a Buchner funnel and washed with methanol and H_2O a few times. Then the solid was dried to give white solid (95.5 mg, 46.8 % yield, M_n = 15389, M_w/M_n = 1.667). ^{1}H NMR (400 MHz, CHLOROFORM-D) δ 8.03 – 7.24 (m, 234H), 7.22 – 5.51 (m, 333H), 1.24 (br, 12H).

Synthesis of **Polymer 1A**

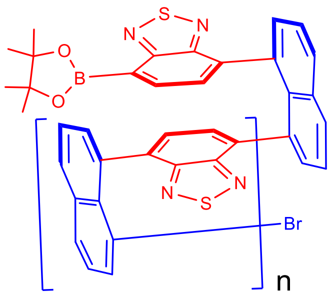

The same synthesis procedure as Polymer **1B**. Yellow solid (202.0 mg, 86.7 % yield, M_n = 11522, M_w/M_n = 2.421). ^{1}H NMR (400 MHz, CHLOROFORM-D) δ 8.29 – 7.24 (m, 233H), 7.19 – 6.17 (m, 166H), 1.23 (br, 12H).

Synthesis of **Polymer 1C**

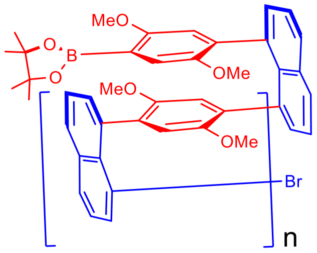

The same synthesis procedure as Polymer **1B**. Pale white solid (91.0 mg, 38.9% yield, M_n = 13488, M_w/M_n = 1.546). ^{1}H NMR (400 MHz, CHLOROFORM-D) δ 8.14 – 7.28 (m, 155H), 7.21 – 5.82 (m, 131H), 3.87 – 3.08 (m, 207H), 1.23 (br, 12H).

Synthesis of **Polymer 1D**

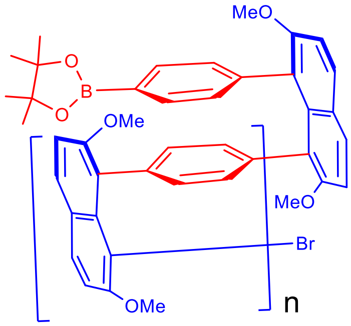

The same synthesis procedure as Polymer **1B**. Pale yellow solid (87.0 mg, 74.3 % yield, M_n = 19348, M_w/M_n = 1.524). ^{1}H NMR (400 MHz, CHLOROFORM-D) δ 7.95 – 7.25 (m, 160H), 7.24 – 6.64 (m, 169H), 4.16 – 3.07 (m, 97H), 1.27 (s, 12H).

Synthesis of **Polymer 1E**

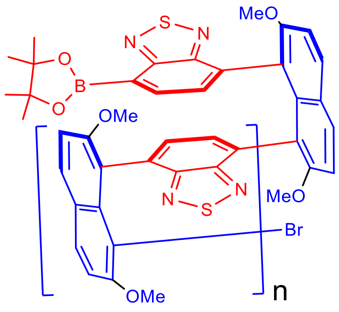

The same synthesis procedure as Polymer **1B**. Brown yellow solid (80.0 mg, 61.1% yield, M_n = 8526, M_w/M_n = 1.388). ^{1}H NMR (400 MHz, CHLOROFORM-D) δ 8.18 – 7.29 (m, 160H), 7.21 – 6.49 (m, 50H), 4.04 – 3.47 (m, 116H), 1.26 (s, 12H).

Synthesis of **Polymer 1F**

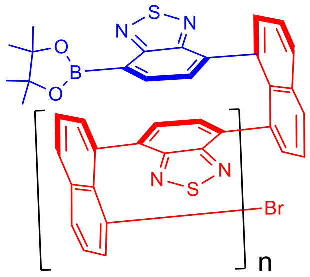

The same synthesis procedure as Polymer **1B**. Yellow solid (269.0 mg, 74.0% yield, M_n = 10149, M_w/M_n = 1.432). ^{1}H NMR (400 MHz, CHLOROFORM-D) δ 8.24 – 7.81 (m, 57H), 7.80 – 7.49 (m, 96H), 7.48 – 7.25 (m, 76H), 7.20 – 6.45 (m, 123H), 1.23 (s, 12H).

3. NMR Spectrums and GPC data

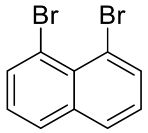

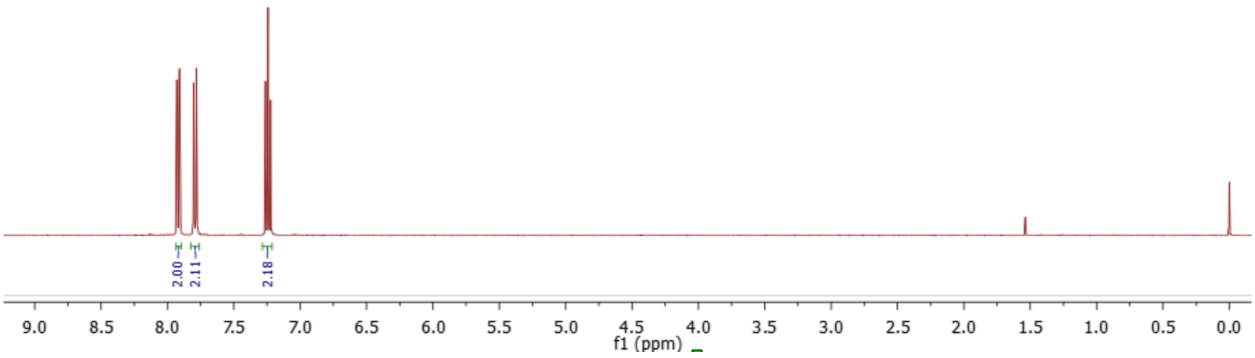

Figure S1. ^{1}H NMR spectrum of **1**

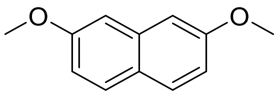

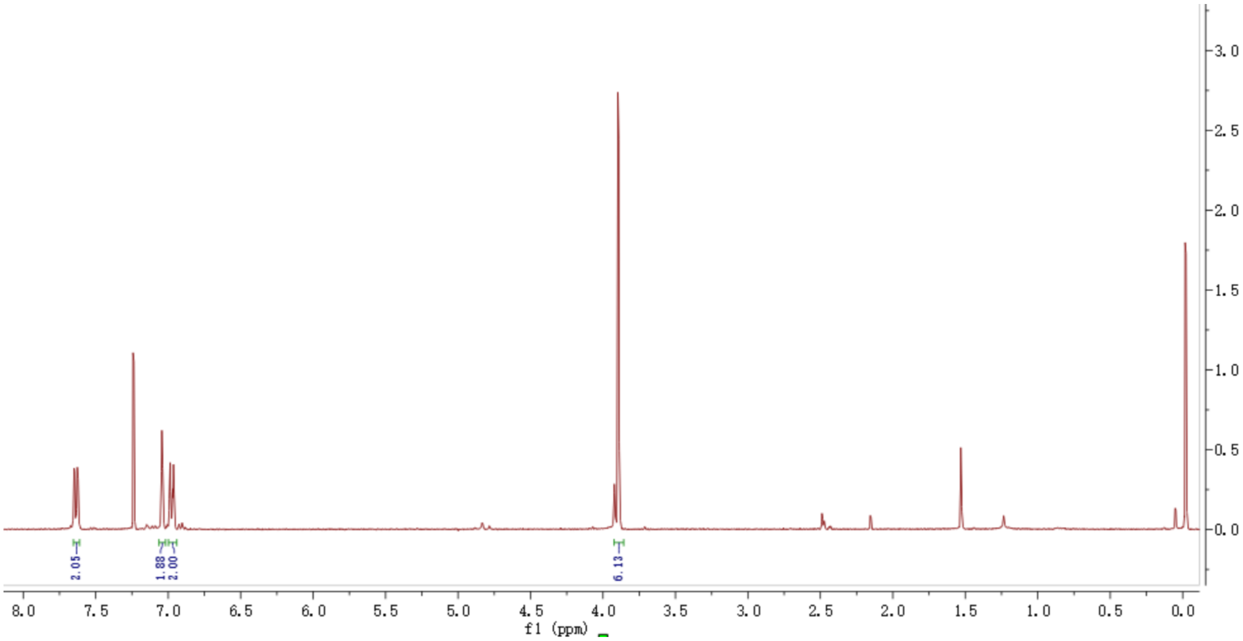

Figure S2. ^{1}H NMR spectrum of **2-2**

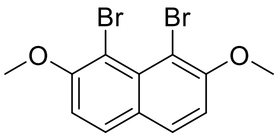

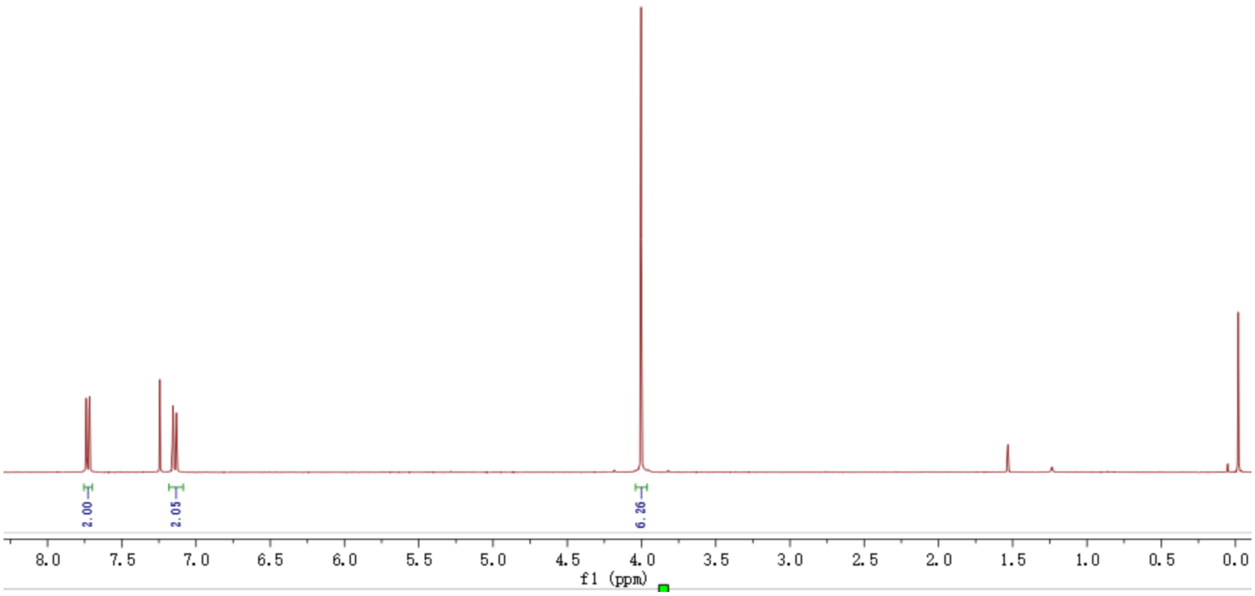

Figure S3. ^{1}H NMR spectrum of **2**

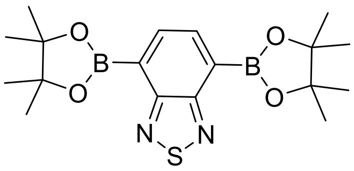

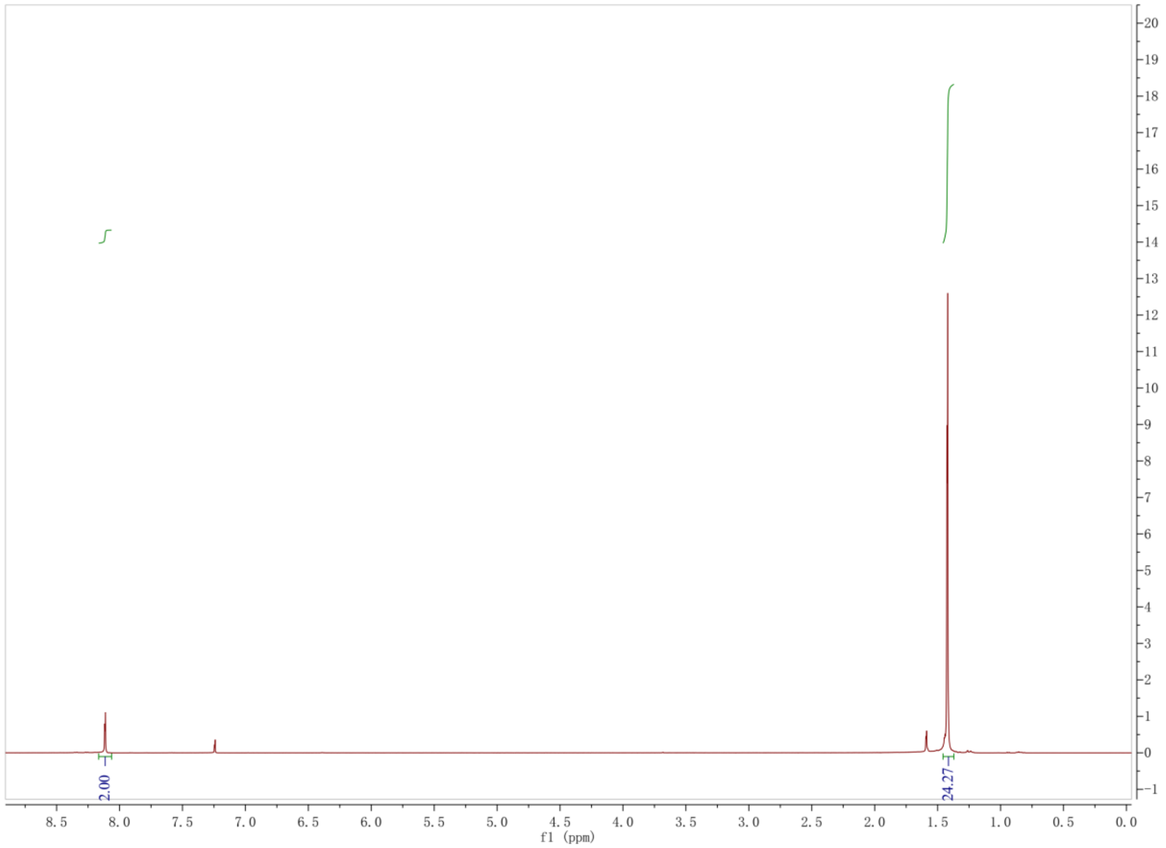

Figure S4. ^{1}H NMR spectrum of **3**

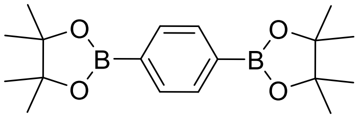

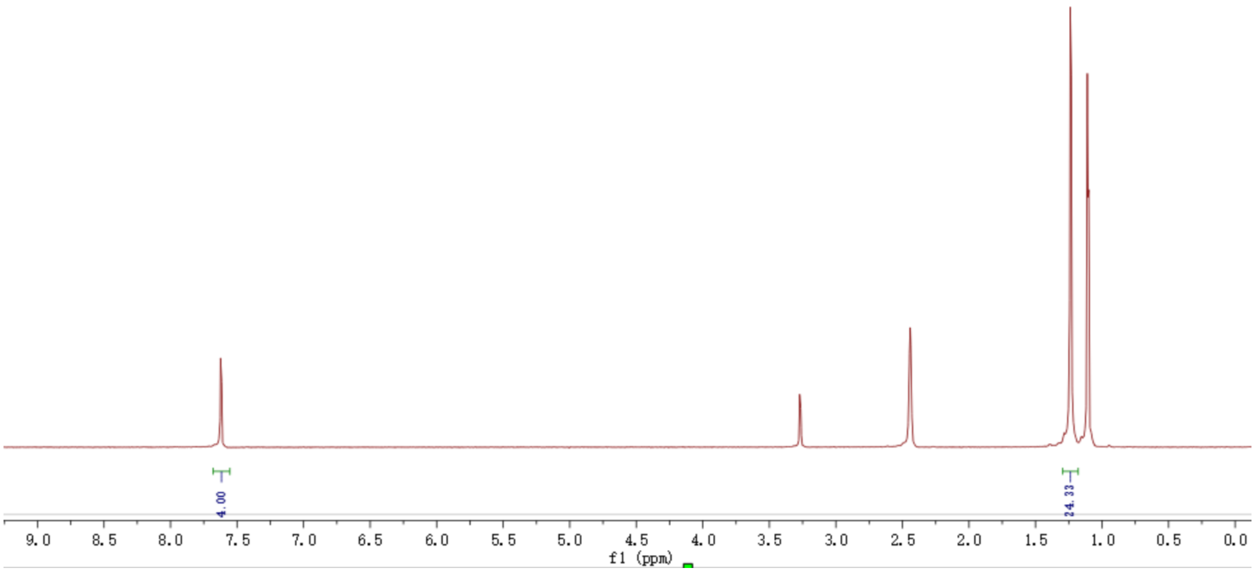

Figure S5. ^{1}H NMR spectrum of **4**

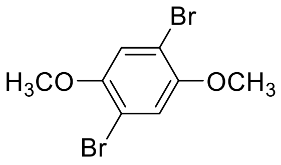

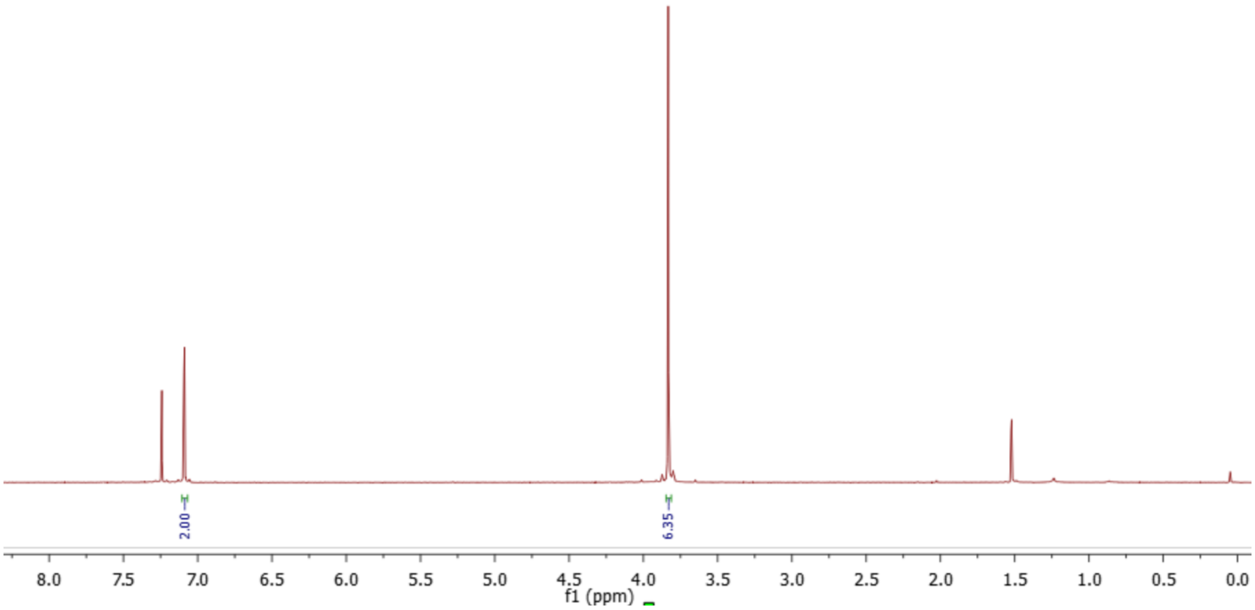

Figure S6. ^{1}H NMR spectrum of **5-1**

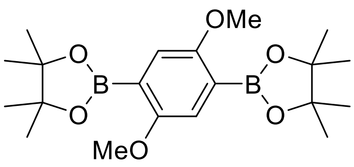

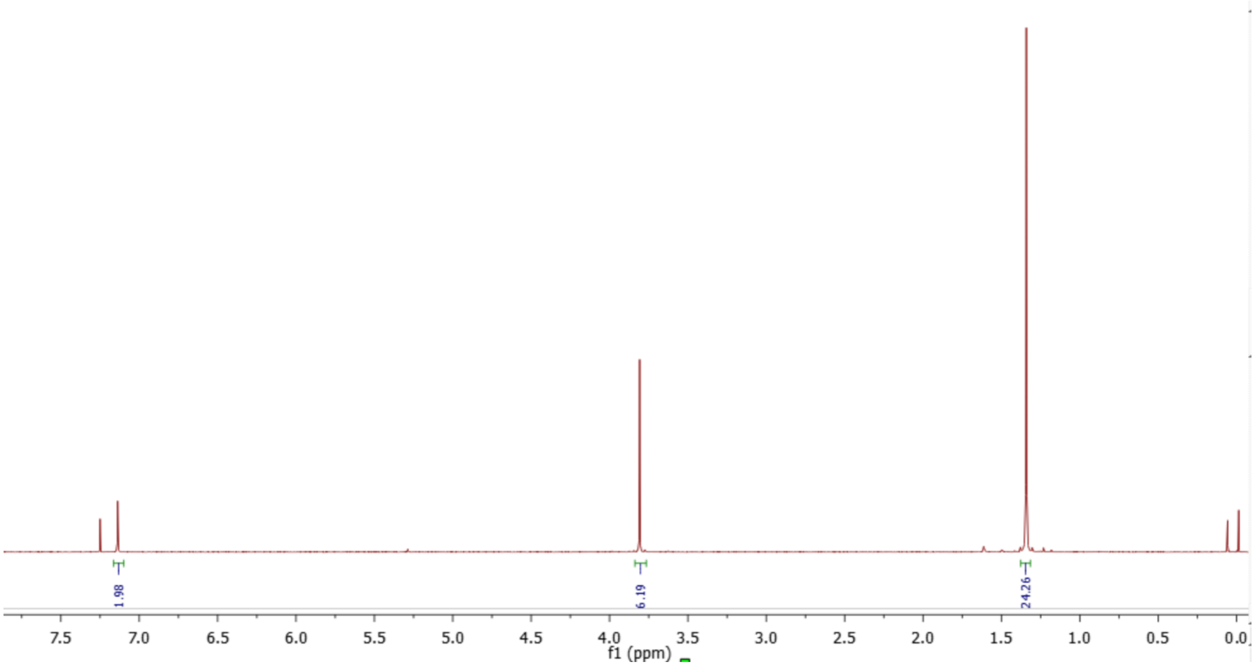

Figure S7. ^{1}H NMR spectrum of **5**

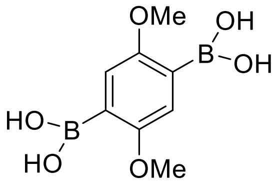

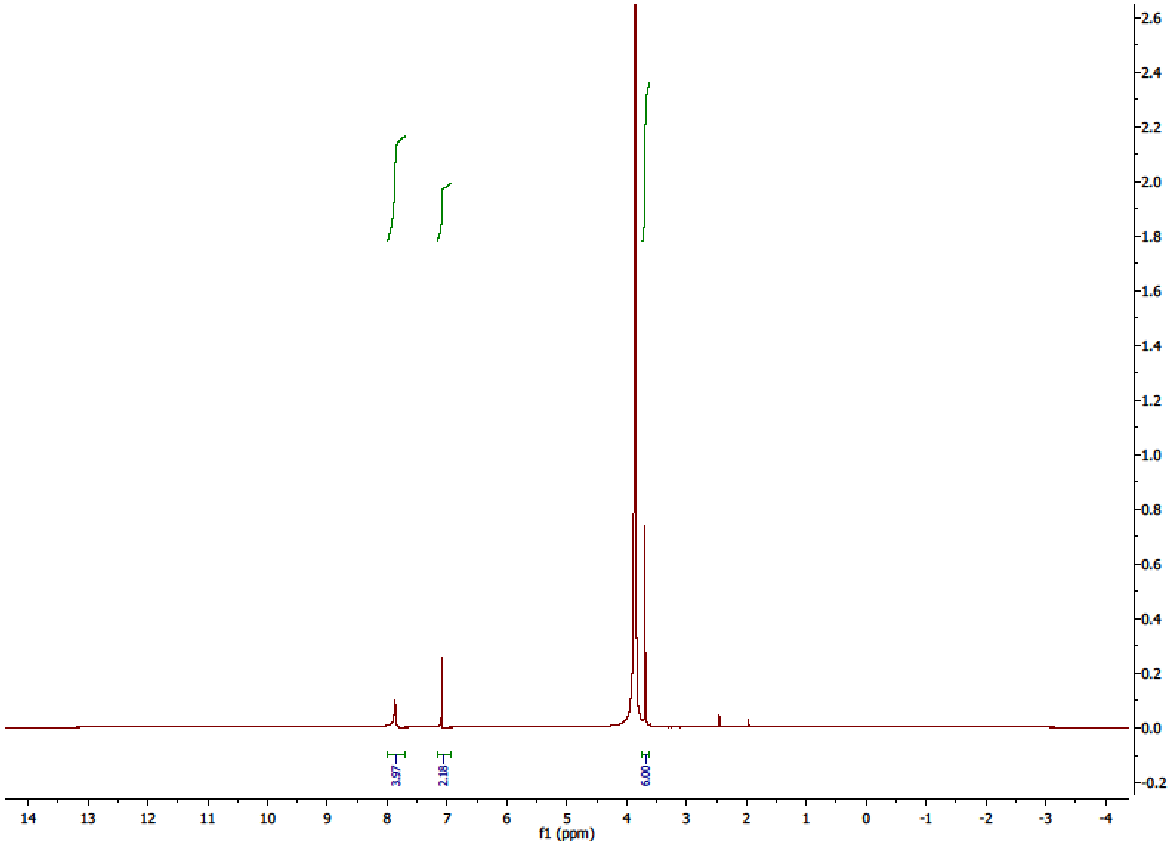

Figure S8. ^{1}H NMR spectrum of **6**

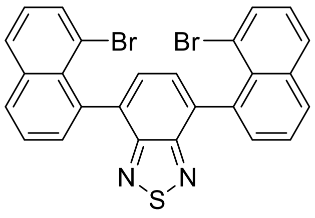

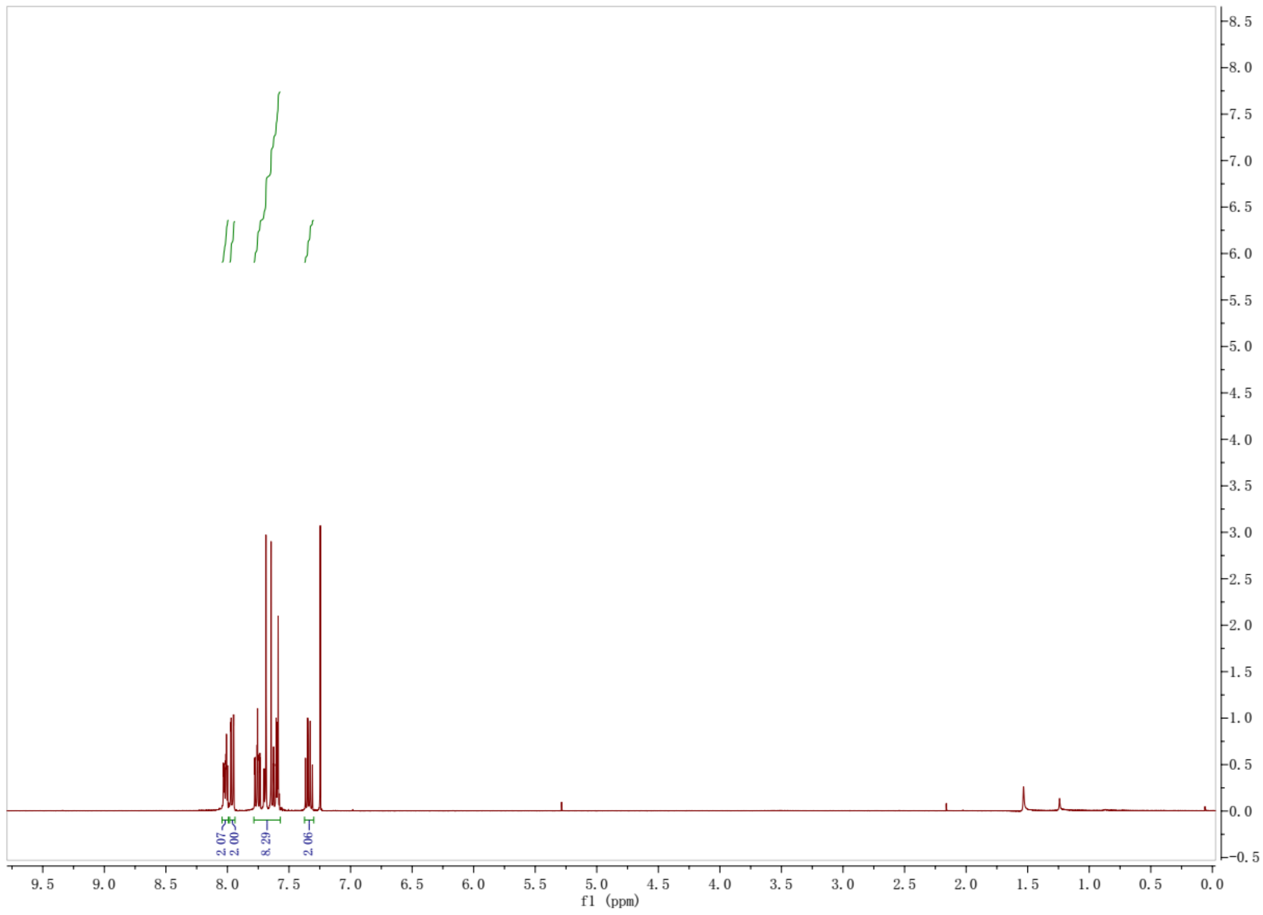

Figure S9. ^{1}H NMR spectrum of **12**

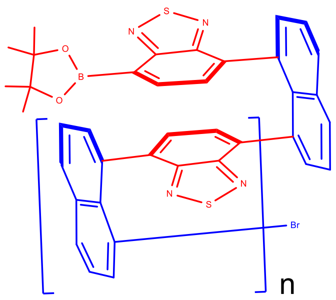

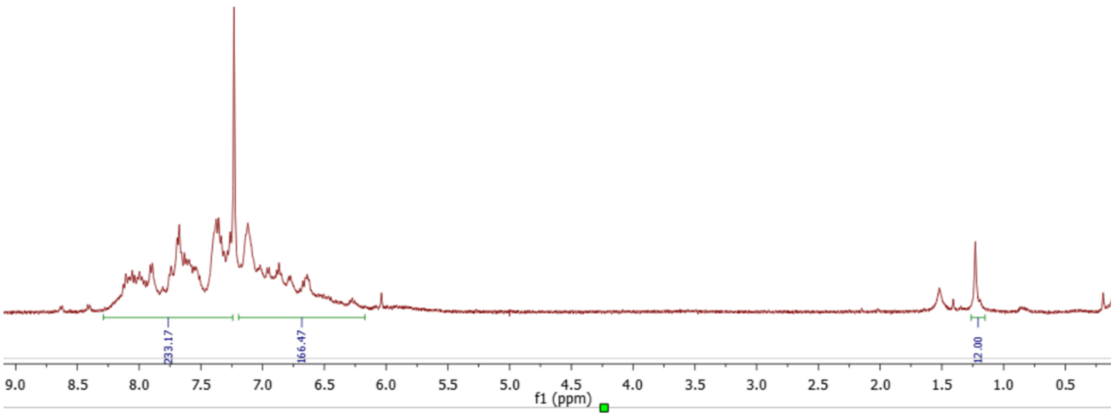

Figure S10. ^{1}H NMR spectrum of **1A**

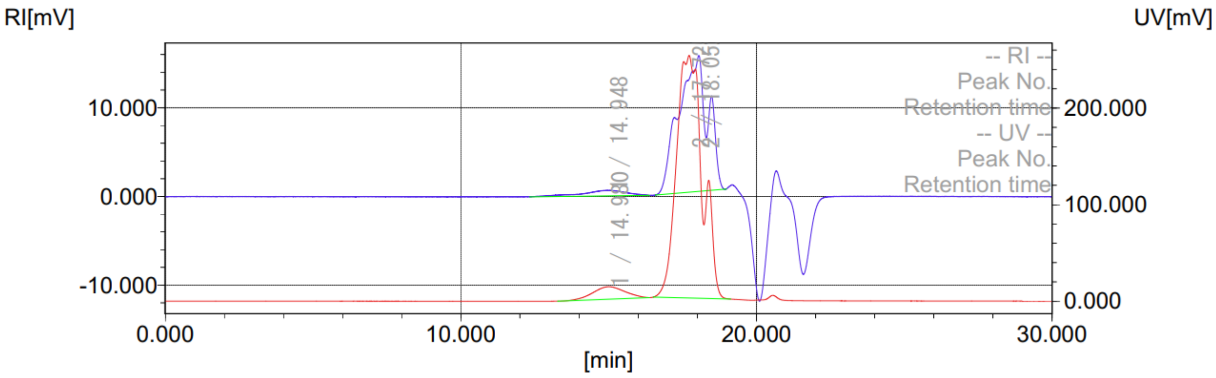

Result of molecular weight calculation (RI)

Peak 1 Base Peak

	[min]	[mV]	[mol]
Peak start	12.337	-0.055	262,159
Peak top	14.948	0.694	12,059
Peak end	16.372	0.117	2,252
Height [mV]			0.638
Area [mV*sec]			64.575
Height% [%]			3.991
[eta]			27891.57325

Mn	11,522
Mw	27,892
Mz	73,366
Mz+1	124,924
Mv	27,892
Mp	11,779
Mz/Mw	2.630
Mw/Mn	2.421
Mz+1/Mw	4.479

Figure S11. GPC data of **1A**

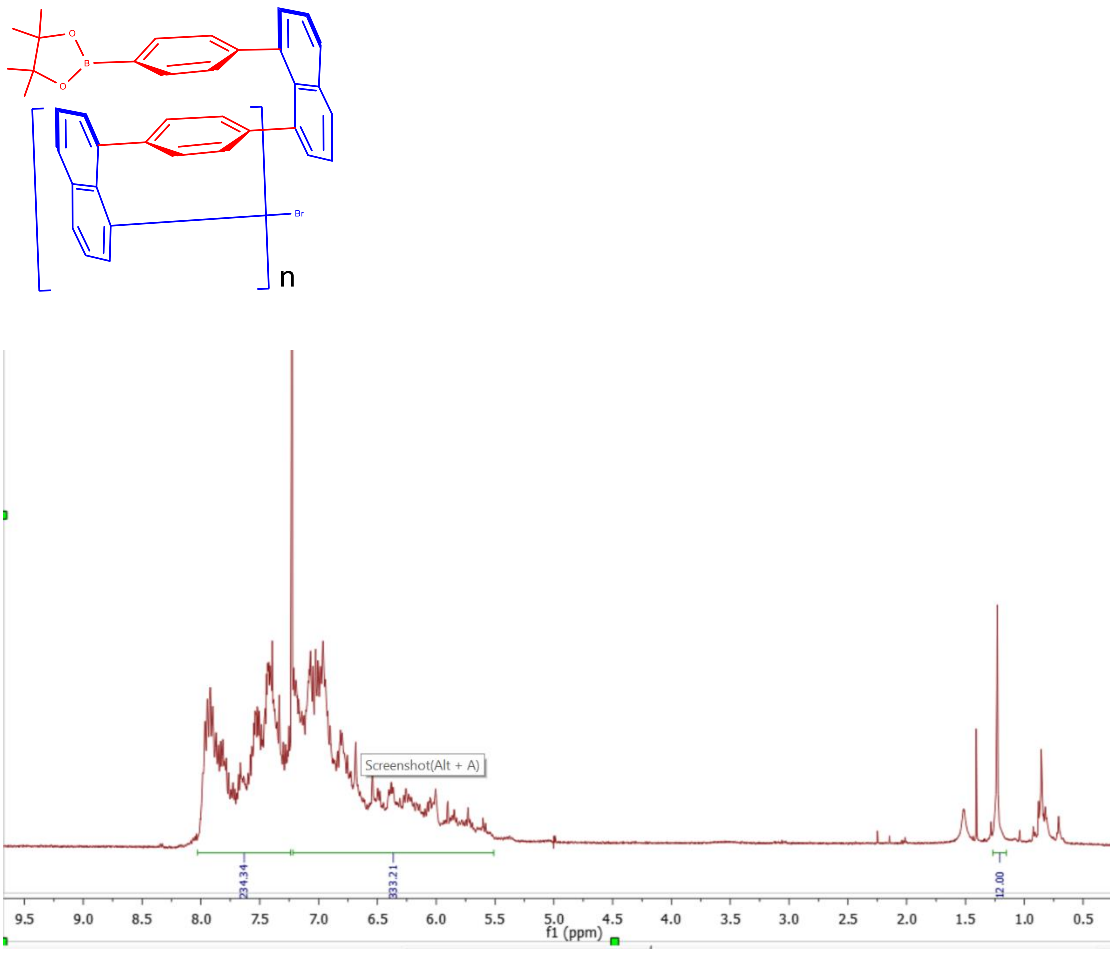

Figure S12. ^{1}H NMR spectrum of **1B**

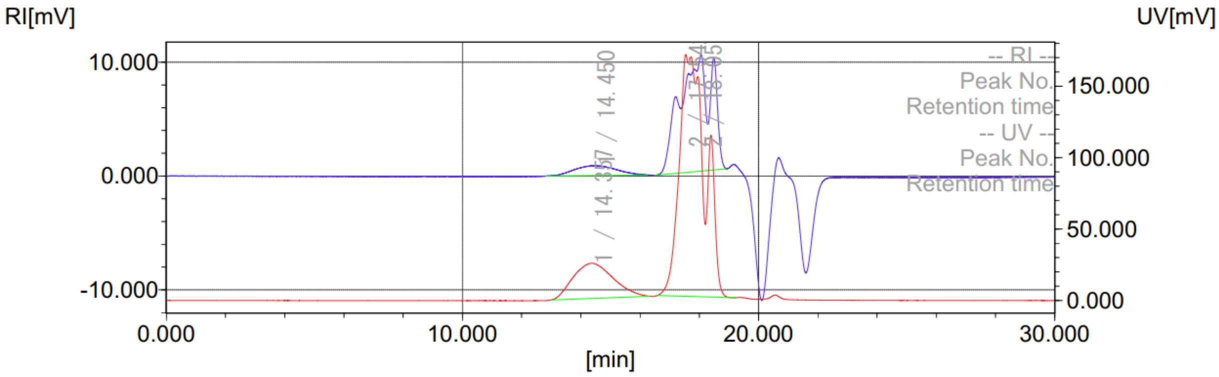

Result of molecular weight calculation (RI)

Peak 1 Base Peak

	[min]	[mV]	[mol]		
				Mn	15,389
Peak start	12.822	0.001	147,991	Mw	25,652
Peak top	14.450	0.931	21,701	Mz	39,254
Peak end	16.233	0.085	2,650	Mz+1	53,807
				Mv	25,652
Height [mV]			0.890	Mp	21,702
Area [mV*sec]			85.073	Mz/Mw	1.530
Height% [%]			8.001	Mw/Mn	1.667
[eta]			25652.08570	Mz+1/Mw	2.098

Figure S13. GPC data of **1B**

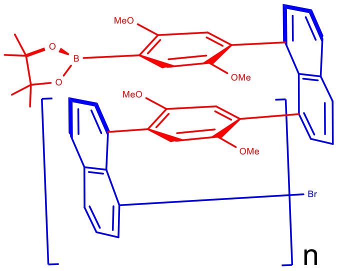

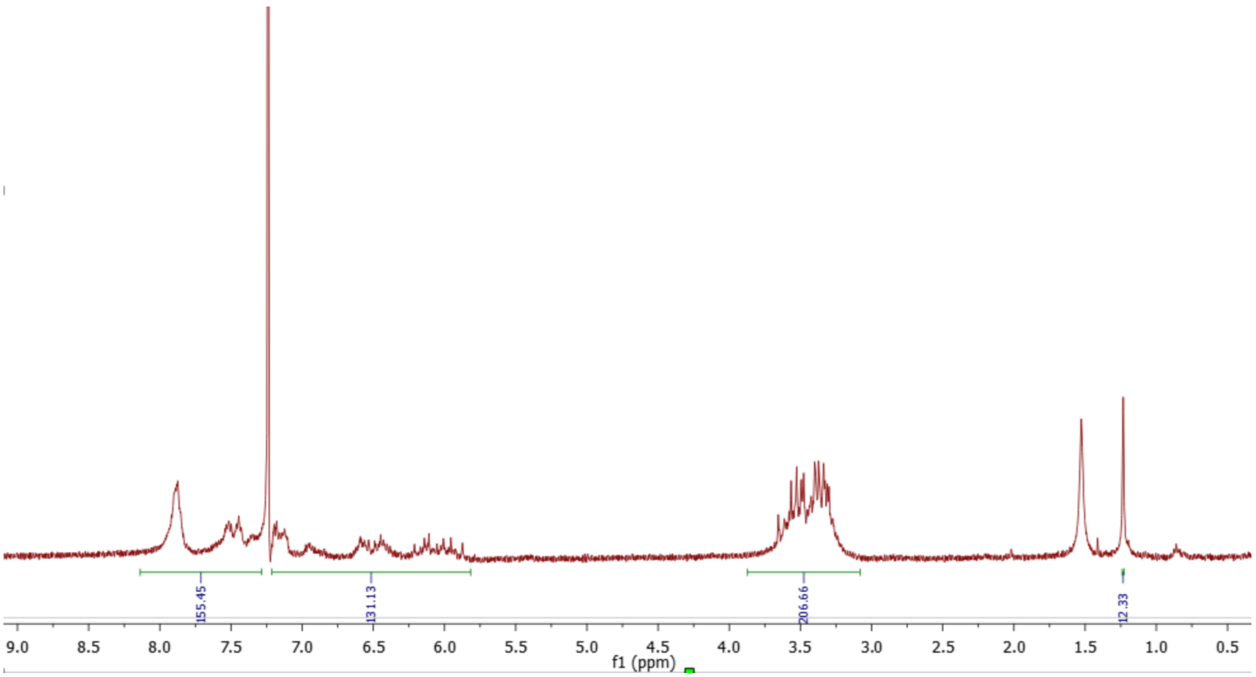

Figure S14. ^{1}H NMR spectrum of **1C**

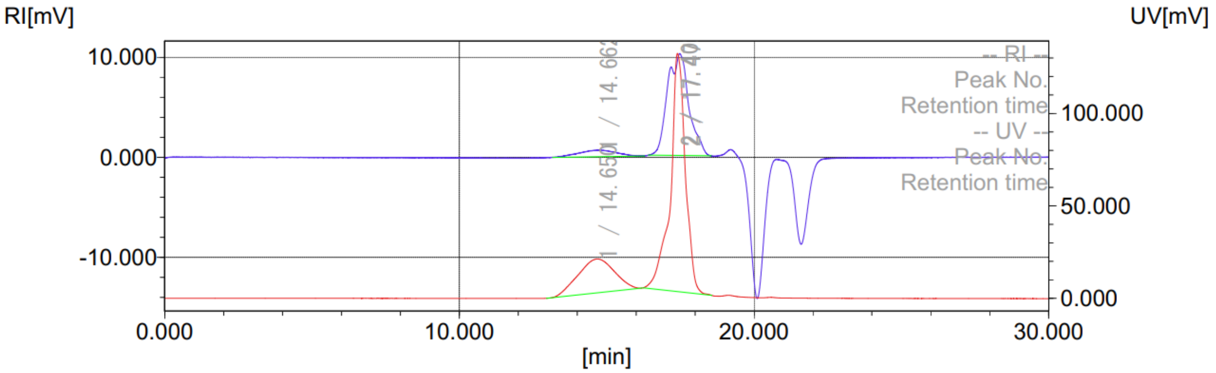

Result of molecular weight calculation (RI)

Peak 1 Base Peak

	[min]	[mV]	[mol]		
				Mn	13,488
Peak start	13.167	-0.010	98,534	Mw	20,847
Peak top	14.662	0.723	16,909	Mz	30,988
Peak end	16.280	0.128	2,509	Mz+1	41,923
				Mv	20,847
Height [mV]			0.667	Mp	16,909
Area [mV*sec]			57.690	Mz/Mw	1.486
Height% [%]			6.136	Mw/Mn	1.546
[eta]			20847.05645	Mz+1/Mw	2.011

Figure S15. GPC data of **1C**

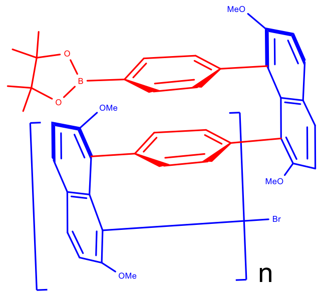

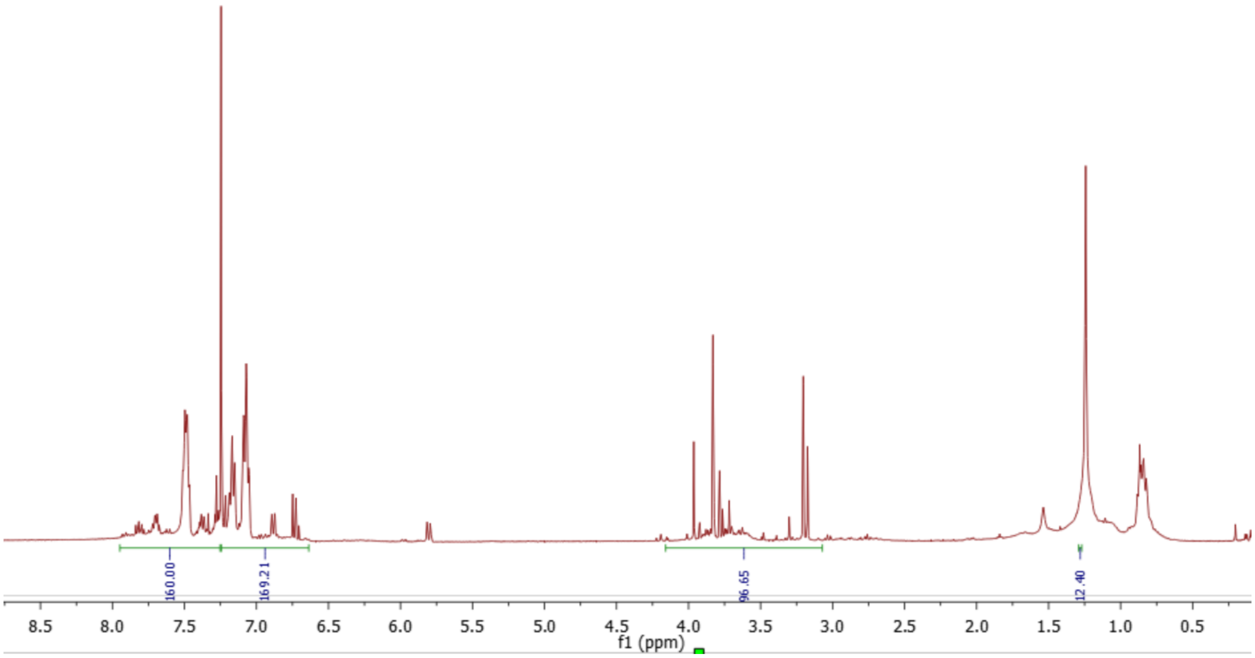

Figure S16. ^{1}H NMR spectrum of **1D**

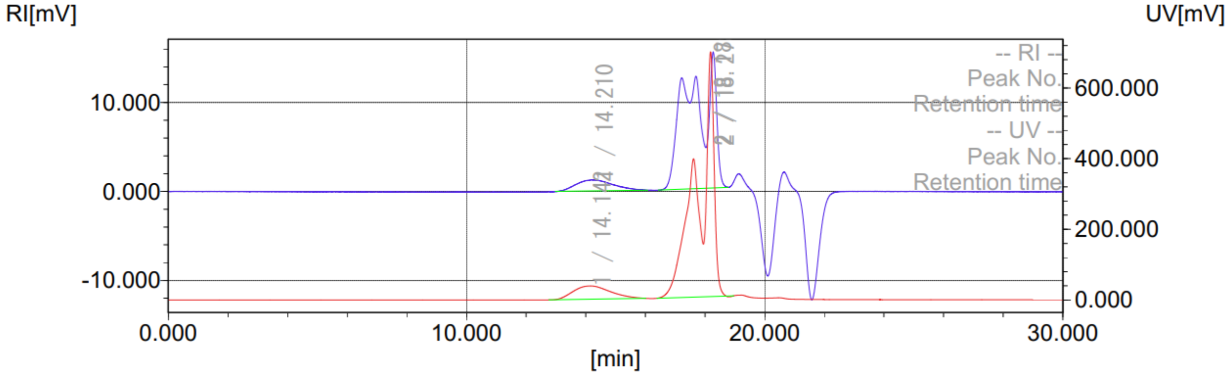

Result of molecular weight calculation (RI)

Peak 1 Base Peak

	[min]	[mV]	[mol]
Peak start	12.982	0.003	122,549
Peak top	14.210	1.305	28,799
Peak end	16.072	0.136	3,207
Height [mV]			1.249
Area [mV*sec]			106.342
Height% [%]			7.551
[eta]			29481.98502

Mn	19,348
Mw	29,482
Mz	40,671
Mz+1	51,177
Mv	29,482
Mp	28,128
Mz/Mw	1.380
Mw/Mn	1.524
Mz+1/Mw	1.736

Figure S17. GPC data of **1D**

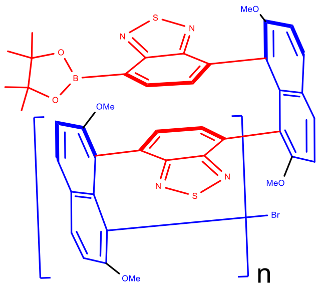

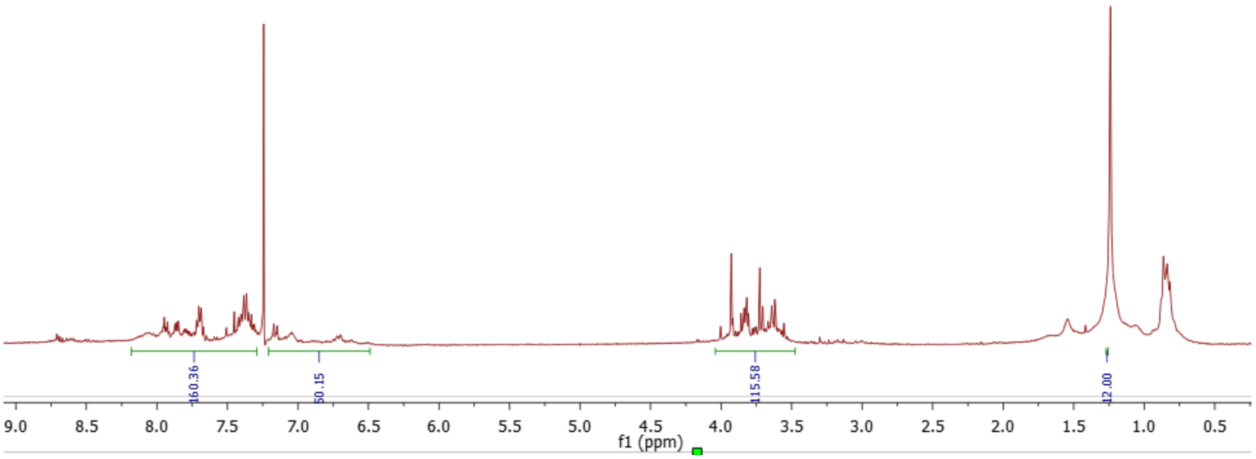

Figure S18. ^{1}H NMR spectrum of **1E**

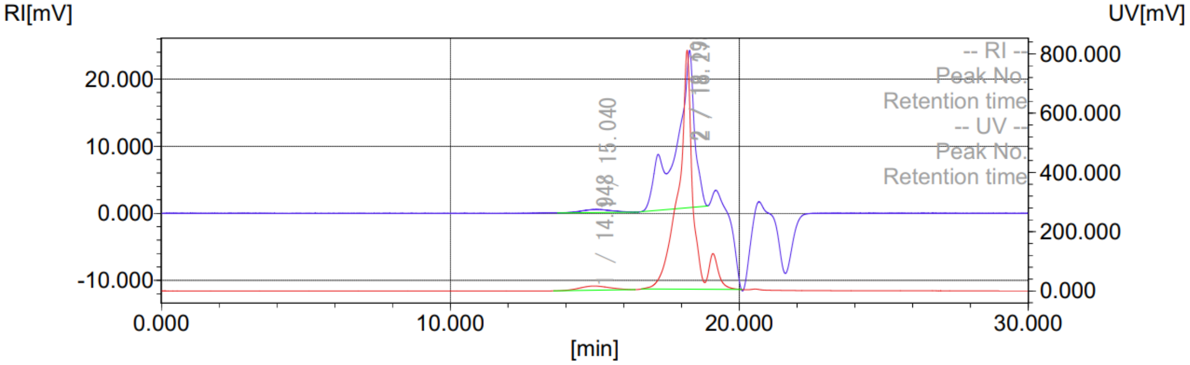

Result of molecular weight calculation (RI)

Peak 1 Base Peak

	[min]	[mV]	[mol]		
Peak start	13.720	0.053	51,318	Mn	8,526
Peak top	15.040	0.593	10,824	Mw	11,835
Peak end	16.510	0.155	1,913	Mz	15,976
				Mz+1	20,781
				Mv	11,835
Height [mV]			0.492	Mp	10,552
Area [mV*sec]			34.116	Mz/Mw	1.350
Height% [%]			2.054	Mw/Mn	1.388
[eta]			11835.06721	Mz+1/Mw	1.756

Figure S19. GPC data of **1E**

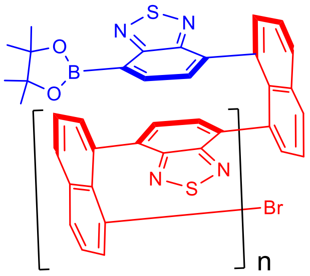

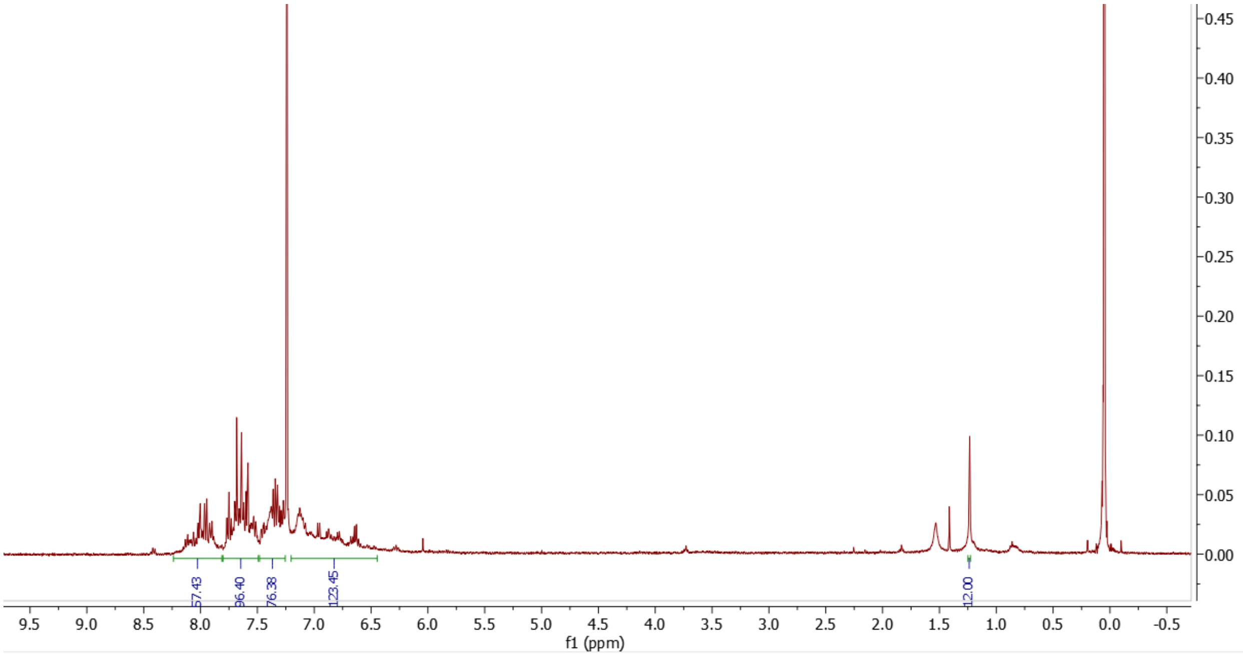

Figure S20. ^{1}H NMR spectrum of **1F**

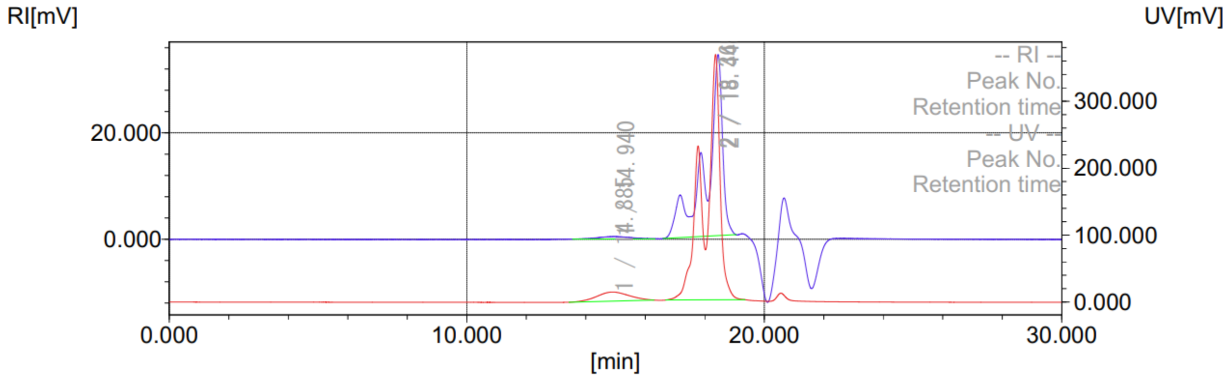

Result of molecular weight calculation (RI)

Peak 1 Base Peak

	[min]	[mV]	[mol]		
Peak start	13.558	0.014	62,093	Mn	10,149
Peak top	14.940	0.528	12,178	Mw	14,533
Peak end	16.327	0.084	2,374	Mz	20,517
				Mz+1	27,523
				Mv	14,533
Height [mV]			0.479	Mp	11,325
Area [mV*sec]			35.148	Mz/Mw	1.412
Height% [%]			1.388	Mw/Mn	1.432
[eta]			14532.93002	Mz+1/Mw	1.894

Figure S21. GPC data of **1F**

**Sample Name:**	7 in methanol again f avg		
**SOP Name:**	mansettings.nano		
**File Name:**	experiment1.dts	**Dispersant Name:**	Methanol
**Record Number:**	21	**Dispersant RI:**	1.326
**Material RI:**	1.59	**Viscosity (cP):**	0.5476
**Material Absorbtion:**	0.010	**Measurement Date and Time:**	Thursday, November 12, 2020 2:40:5...

**Temperature (°C):**	25.0	**Duration Used (s):**	60
**Count Rate (kcps):**	389.5	**Measurement Position (mm):**	3.00
**Cell Description:**	Disposable micro cuvette (40µl)	**Attenuator:**	10

			**Size (d.nm):**	**% Volume:**	**St Dev (d.n...**
**Z-Average (d.nm):**	999.9	**Peak 1:**	0.6906	66.7	0.1173
**PdI:**	0.483	**Peak 2:**	252.7	33.3	38.21
**Intercept:**	0.174	**Peak 3:**	0.000	0.0	0.000

Result quality : **Refer to quality report**

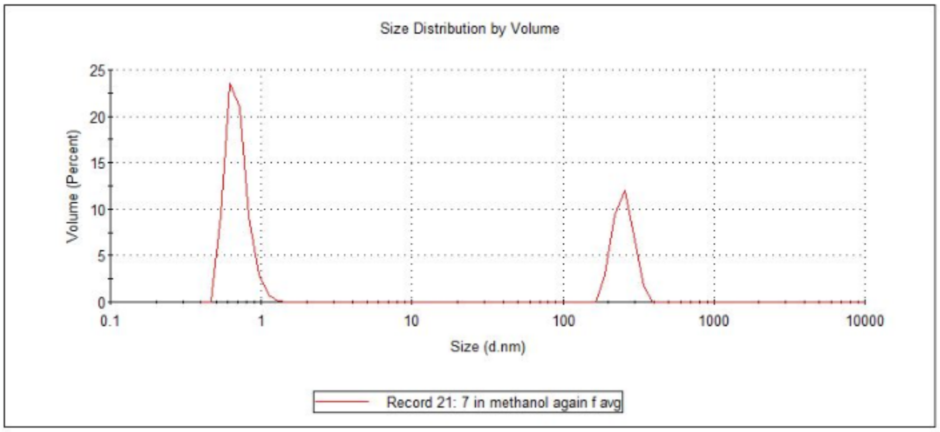

Figure S22. DLS size distributions curve showing hydrodynamic diameters of monomer **7**.

**Sample Name:**	1B in methanol 1		
**SOP Name:**	mansettings.nano		
**File Name:**	experiment1.dts	**Dispersant Name:**	Methanol
**Record Number:**	8	**Dispersant RI:**	1.326
**Material RI:**	1.59	**Viscosity (cP):**	0.5476
**Material Absorbtion:**	0.010	**Measurement Date and Time:**	Thursday, November 12, 2020 1:56:0.

**Temperature (°C):**	25.0	**Duration Used (s):**	60
**Count Rate (kcps):**	234.4	**Measurement Position (mm):**	3.00
**Cell Description:**	Disposable micro cuvette (40µl)	**Attenuator:**	6

			**Size (d.nm):**	**% Volume:**	**St Dev (d.n...**
**Z-Average (d.nm):**	1345	**Peak 1:**	142.8	17.5	17.86
**PdI:**	0.717	**Peak 2:**	1353	82.5	183.9
**Intercept:**	0.943	**Peak 3:**	0.000	0.0	0.000

Result quality : **Refer to quality report**

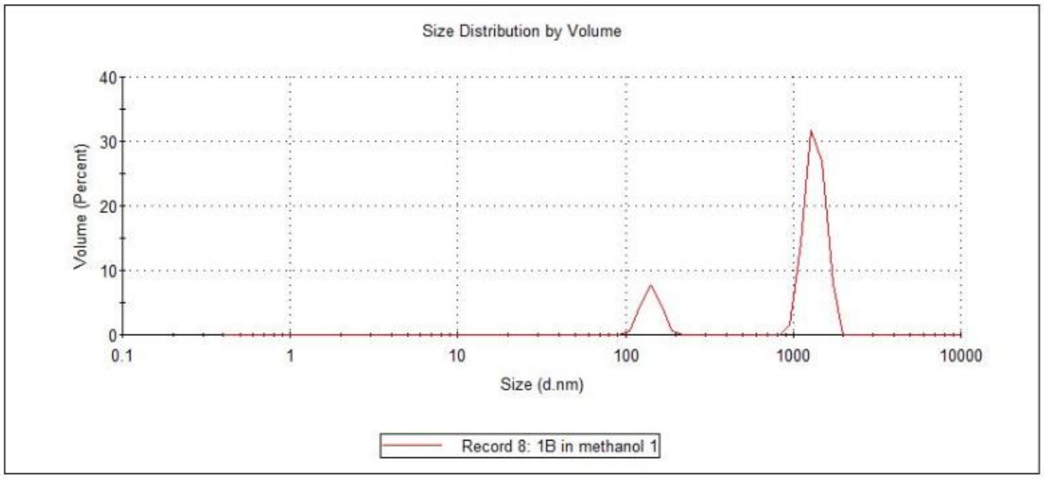

Figure S23. DLS size distributions curve showing hydrodynamic diameters of polymer **1B**.

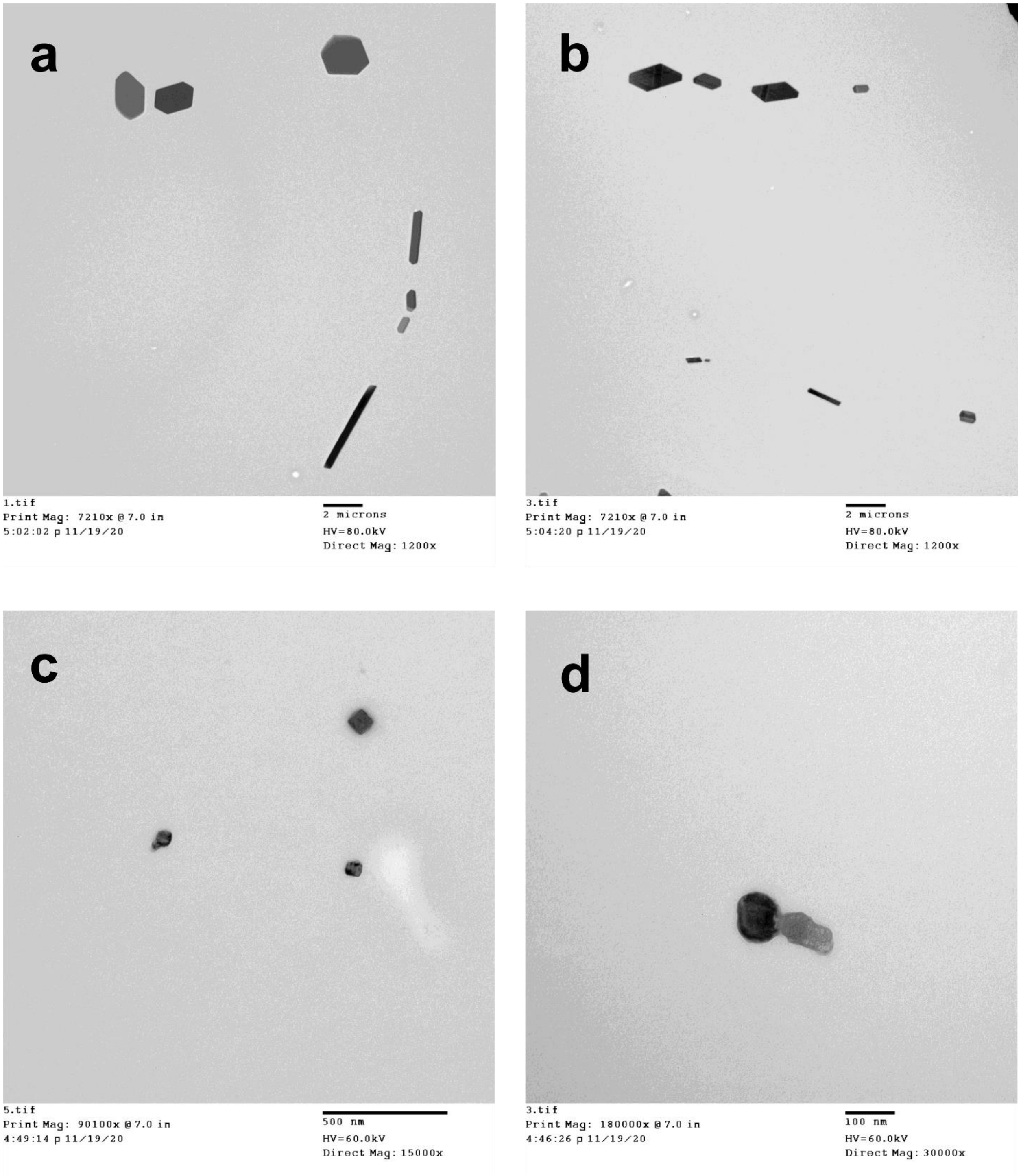

Figure S24. TEM images of monomer **7** and polymer **1B**. (a & b are crystalized monomer in hexagonal or rod shape; c & d are amorphous polymer.)

4. X-ray Data

General Data Collection

Data were collected on a Rigaku XtaLAB Synergy-*i* Kappa diffractometer equipped with a PhotonJet-*i* X-ray source operated at 50 W (50kV, 1 mA) to generate Cu Kα radiation (λ = 1.54178 Å) and a HyPix-6000HE HPC detector. Crystals were transferred from the vial and placed on a glass slide in polyisobutylene. A Zeiss Stemi 305 microscope was used to identify a suitable specimen for X-ray diffraction from a representative sample of the material. The crystal and a small amount of the oil were collected on a MīTiGen cryoloop and transferred to the instrument where it was placed under a cold nitrogen stream (Oxford) maintained at 100K throughout the duration of the experiment. The sample was optically centered with the aid of a video camera to insure that no translations were observed as the crystal was rotated through all positions.

A unit cell collection was then carried out. After it was determined that the unit cell was not present in the CCDC database a data collection strategy was calculated by *CrysAlis*^{Pro}[3]. The crystal was measured for size, morphology, and color.

(1)

Refinement Details

After data collection, the unit cell was re-determined using a subset of the full data collection. Intensity data were corrected for Lorentz, polarization, and background effects using the *CrysAlis*^{Pro} [3]. A numerical absorption correction was applied based on a Gaussian integration over a multifaceted crystal and followed by a semi-empirical correction for adsorption applied using the program *SCALE3 ABSPACK* [4]. The *SHELXL-2014* [5], series of programs was used for the solution and refinement of the crystal structure. Hydrogen atoms bound to carbon atoms were located in the difference Fourier map and were geometrically constrained using the appropriate AFIX commands.

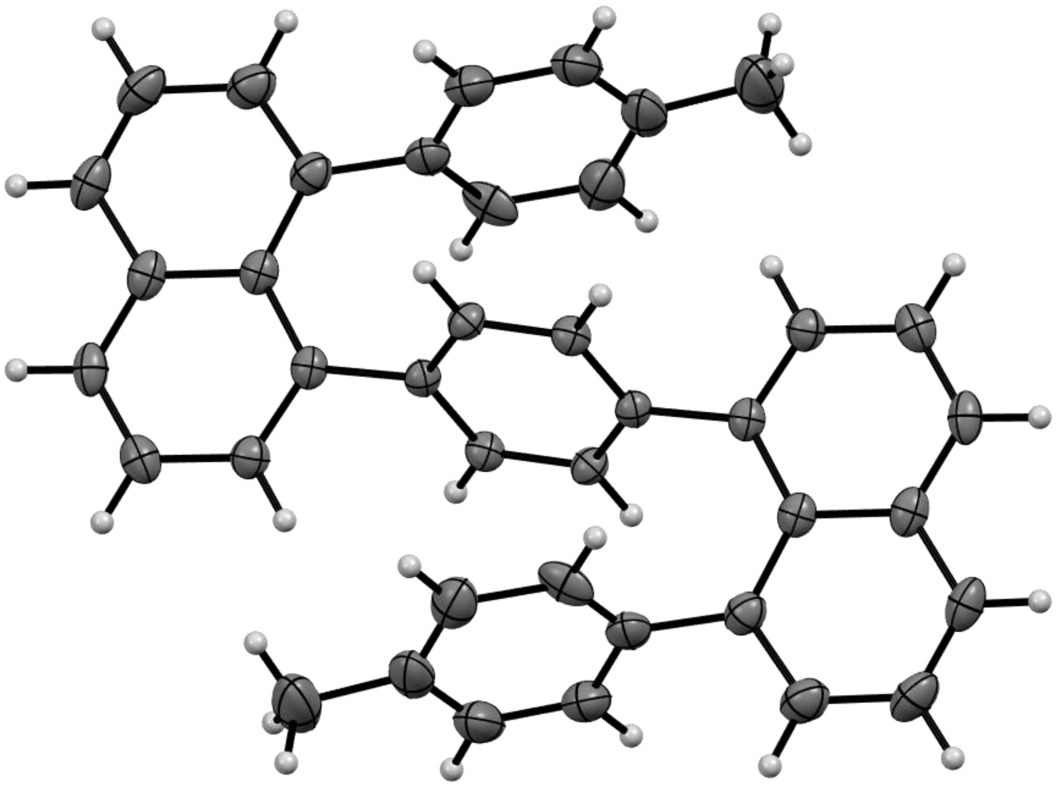

Figure S25. X-ray structure of **7**.

The thermal ellipsoids are represented at 50% probability. Carbon and hydrogen atoms are represented by gray and white ellipsoids, respectively.

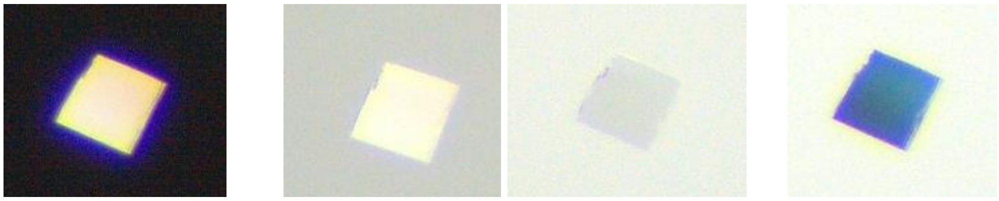

Crystal used for single crystal X-ray diffraction analysis.

Table S1. Crystal data and structure refinement for **7**.

Identification code	li19_29
Crystal Color	colourless
Crystal Habit	plate

Empirical formula	C40 H30
Formula weight	510.64
Temperature	136(2) K
Wavelength	1.54178 Å
Crystal system	Monoclinic
Space group	$P2_1/c$
Unit cell dimensions	a = 11.6542(2) Å alpha = 90 °.
	b = 16.9311(2) Å beta = 102.021(2) °.
	c = 7.10450(10) Å gamma = 90 °.
Volume	1371.11(4) $Å^3$
Z	2
Calculated density	1.237 Mg/m^3
Absorption coefficient	0.527 mm^{-1}
F(000)	540
Crystal size	0.149 x 0.136 x 0.026 mm
Theta range for data collection	3.878 to 77.222 °.
Limiting indices	-13<=h<=14, -14<=k<=21, -8<=l<=8
Reflections collected / unique	13007 / 2826 [R(int) = 0.0311]
Completeness to theta = 67.679°	99.8 %
Refinement method	Full-matrix least-squares on F^2
Data / restraints / parameters	2826 / 0 / 182
Goodness-of-fit on F^2	1.066
Final R indices [I>2sigma(I)]	R1 = 0.0569, wR2 = 0.1551
R indices (all data)	R1 = 0.0641, wR2 = 0.1615
Largest diff. peak and hole	0.836 and -0.239 e.$Å^{-3}$

Table S2. Atomic coordinates (x 10^4) and equivalent isotropic displacement parameters (Å^2 x 10^3) for li19_29. U(eq) is defined as one third of the trace of the orthogonalized Uij tensor.

	x	y	z	U(eq)
C(1)	4616(1)	4825(1)	11668(2)	24(1)
C(2)	4267(1)	4357(1)	10044(2)	24(1)
C(3)	4636(1)	4533(1)	8350(2)	22(1)
C(4)	4319(2)	4018(1)	6613(2)	24(1)
C(5)	5252(2)	3654(1)	6055(2)	28(1)
C(6)	5103(2)	3104(1)	4538(3)	32(1)
C(7)	4001(2)	2921(1)	3582(2)	31(1)
C(8)	3008(2)	3281(1)	4062(2)	29(1)
C(9)	3142(2)	3857(1)	5584(2)	26(1)
C(10)	2088(2)	4208(1)	5963(3)	31(1)
C(11)	1007(2)	3959(1)	4931(3)	39(1)
C(12)	894(2)	3386(1)	3474(3)	41(1)
C(13)	1870(2)	3060(1)	3037(3)	37(1)
C(14)	2049(2)	4852(1)	7401(3)	34(1)
C(15)	2471(2)	5573(1)	7145(3)	37(1)
C(16)	2403(2)	6191(1)	8516(3)	45(1)
C(17)	1845(2)	6058(1)	10040(3)	48(1)
C(18)	1415(2)	5306(2)	10247(3)	48(1)
C(19)	1505(2)	4712(1)	8941(3)	41(1)
C(20)	1666(2)	6714(2)	11387(4)	70(1)

Table S3. Bond lengths [Å] and angles [°] for li19_29.

C(1)-C(2)	1.388(2)
C(1)-C(3)#1	1.395(2)
C(1)-H(1)	0.9500
C(2)-C(3)	1.393(2)
C(2)-H(2)	0.9500
C(3)-C(4)	1.493(2)
C(4)-C(5)	1.378(2)
C(4)-C(9)	1.440(2)
C(5)-C(6)	1.407(2)
C(5)-H(5)	0.9500
C(6)-C(7)	1.359(3)
C(6)-H(6)	0.9500
C(7)-C(8)	1.411(3)
C(7)-H(7)	0.9500
C(8)-C(13)	1.423(2)
C(8)-C(9)	1.441(2)
C(9)-C(10)	1.439(2)
C(10)-C(11)	1.385(3)
C(10)-C(14)	1.501(3)
C(11)-C(12)	1.405(3)
C(11)-H(11)	0.9500
C(12)-C(13)	1.357(3)
C(12)-H(12)	0.9500
C(13)-H(13)	0.9500
C(14)-C(15)	1.342(3)
C(14)-C(19)	1.394(3)
C(15)-C(16)	1.443(3)

C(15)-H(15)	0.9500
C(16)-C(17)	1.393(3)
C(16)-H(16)	0.9500
C(17)-C(18)	1.387(4)
C(17)-C(20)	1.509(3)
C(18)-C(19)	1.388(3)
C(18)-H(18)	0.9500
C(19)-H(19)	0.9500
C(20)-H(20A)	0.9800
C(20)-H(20B)	0.9800
C(20)-H(20C)	0.9800
C(2)-C(1)-C(3)#1	120.85(15)
C(2)-C(1)-H(1)	119.6
C(3)#1-C(1)-H(1)	119.6
C(1)-C(2)-C(3)	120.45(15)
C(1)-C(2)-H(2)	119.8
C(3)-C(2)-H(2)	119.8
C(2)-C(3)-C(1)#1	118.69(15)
C(2)-C(3)-C(4)	121.48(14)
C(1)#1-C(3)-C(4)	119.70(14)
C(5)-C(4)-C(9)	119.63(15)
C(5)-C(4)-C(3)	115.13(15)
C(9)-C(4)-C(3)	125.17(15)
C(4)-C(5)-C(6)	122.55(16)
C(4)-C(5)-H(5)	118.7
C(6)-C(5)-H(5)	118.7
C(7)-C(6)-C(5)	119.13(16)

C(7)-C(6)-H(6)	120.4
C(5)-C(6)-H(6)	120.4
C(6)-C(7)-C(8)	121.19(16)
C(6)-C(7)-H(7)	119.4
C(8)-C(7)-H(7)	119.4
C(7)-C(8)-C(13)	119.07(16)
C(7)-C(8)-C(9)	120.54(16)
C(13)-C(8)-C(9)	120.38(17)
C(10)-C(9)-C(4)	125.93(15)
C(10)-C(9)-C(8)	117.17(15)
C(4)-C(9)-C(8)	116.89(15)
C(11)-C(10)-C(9)	119.53(17)
C(11)-C(10)-C(14)	115.36(16)
C(9)-C(10)-C(14)	125.10(15)
C(10)-C(11)-C(12)	122.39(19)
C(10)-C(11)-H(11)	118.8
C(12)-C(11)-H(11)	118.8
C(13)-C(12)-C(11)	119.67(17)
C(13)-C(12)-H(12)	120.2
C(11)-C(12)-H(12)	120.2
C(12)-C(13)-C(8)	120.81(17)
C(12)-C(13)-H(13)	119.6
C(8)-C(13)-H(13)	119.6
C(15)-C(14)-C(19)	120.03(19)
C(15)-C(14)-C(10)	120.28(18)
C(19)-C(14)-C(10)	119.57(18)
C(14)-C(15)-C(16)	119.66(19)

C(14)-C(15)-H(15)	120.2
C(16)-C(15)-H(15)	120.2
C(17)-C(16)-C(15)	120.5(2)
C(17)-C(16)-H(16)	119.8
C(15)-C(16)-H(16)	119.8
C(18)-C(17)-C(16)	118.0(2)
C(18)-C(17)-C(20)	120.4(2)
C(16)-C(17)-C(20)	121.5(2)
C(17)-C(18)-C(19)	121.0(2)
C(17)-C(18)-H(18)	119.5
C(19)-C(18)-H(18)	119.5
C(18)-C(19)-C(14)	120.8(2)
C(18)-C(19)-H(19)	119.6
C(14)-C(19)-H(19)	119.6
C(17)-C(20)-H(20A)	109.5
C(17)-C(20)-H(20B)	109.5
H(20A)-C(20)-H(20B)	109.5
C(17)-C(20)-H(20C)	109.5
H(20A)-C(20)-H(20C)	109.5
H(20B)-C(20)-H(20C)	109.5

Symmetry transformations used to generate equivalent atoms: #1 -x+1,-y+1,-z+2

Table S4. Anisotropic displacement parameters ($Å^2 \times 10^3$) for li19_29. The anisotropic displacement factor exponent takes the form: $-2 \pi^2 [h^2 a^{*2}U^{11} + ... + 2 h k a^* b^* U^{12}]$

	U11	U22	U33	U23	U13	U12
C(1)	27(1)	26(1)	20(1)	3(1)	5(1)	0(1)

C(2)	25(1)	23(1)	24(1)	1(1)	4(1)	-2(1)
C(3)	23(1)	23(1)	20(1)	0(1)	1(1)	2(1)
C(4)	30(1)	21(1)	19(1)	2(1)	3(1)	-1(1)
C(5)	30(1)	28(1)	25(1)	0(1)	4(1)	0(1)
C(6)	41(1)	27(1)	28(1)	0(1)	11(1)	4(1)
C(7)	48(1)	23(1)	21(1)	-1(1)	7(1)	-1(1)
C(8)	39(1)	25(1)	21(1)	2(1)	0(1)	-3(1)
C(9)	31(1)	23(1)	22(1)	2(1)	2(1)	-1(1)
C(10)	29(1)	29(1)	33(1)	1(1)	1(1)	-1(1)
C(11)	30(1)	39(1)	45(1)	0(1)	0(1)	0(1)
C(12)	35(1)	42(1)	41(1)	0(1)	-9(1)	-7(1)
C(13)	44(1)	31(1)	29(1)	-2(1)	-4(1)	-7(1)
C(14)	22(1)	42(1)	36(1)	-4(1)	1(1)	3(1)
C(15)	36(1)	33(1)	45(1)	16(1)	14(1)	20(1)
C(16)	40(1)	35(1)	54(1)	-5(1)	-4(1)	7(1)
C(17)	32(1)	53(1)	52(1)	-18(1)	-2(1)	14(1)
C(18)	30(1)	68(2)	48(1)	-13(1)	9(1)	1(1)
C(19)	28(1)	50(1)	45(1)	-5(1)	8(1)	-4(1)
C(20)	49(1)	80(2)	74(2)	-36(2)	-4(1)	20(1)

Table S5. Hydrogen coordinates (x 10^4) and isotropic displacement parameters (Å^2 x 10^3) for li19_29.

	x	y	z	U(eq)
H(1)	4343	4705	12806	29
H(2)	3772	3915	10089	29

H(5)	6025	3778	6720	34
H(6)	5766	2864	4188	38
H(7)	3896	2543	2571	37
H(11)	317	4185	5218	47
H(12)	138	3228	2798	50
H(13)	1793	2680	2035	44
H(15)	2811	5676	6066	44
H(16)	2742	6692	8378	54
H(18)	1052	5197	11299	58
H(19)	1193	4204	9098	49
H(20A)	2310	7094	11505	105
H(20B)	1651	6492	12656	105
H(20C)	920	6980	10875	105

(2)

Refinement Details

After data collection, the unit cell was re-determined using a subset of the full data collection. Intensity data were corrected for Lorentz, polarization, and background effects using the Bruker program APEX 3. A semi-empirical correction for adsorption was applied using the program *SADABS*. The *SHELXL-2014* [5], series of programs was used for the solution and refinement of the crystal structure. After the initial solution was performed, it was evident that there were enantiomeric molecules occupying the same crystallographic space. To model this positional disorder, atoms S1, N1, N2, C1, C2, C3, C4, C5, C6, C7, C8, C9, C10, C23, C24, C25, and C26 had their site occupancies constrained to 0.5. Hydrogen atoms bound to carbon atoms were located in the difference Fourier map and were geometrically constrained using the appropriate AFIX commands. The rigid-bond restraint RIGU was also applied globally to improve the ADPs of the disorder atom sites.

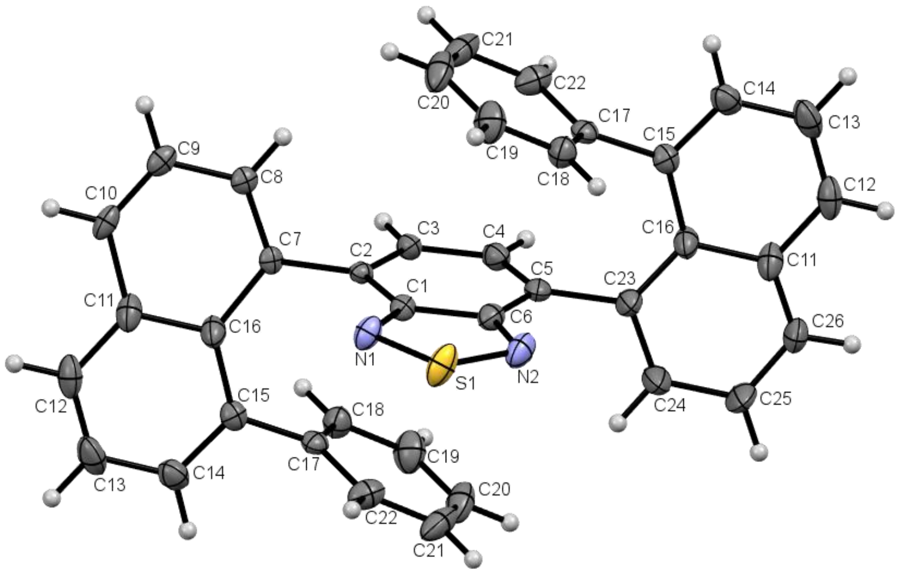

Figure S26. X-ray structure of **8**.

The thermal ellipsoids are represented at 50% probability. Carbon, hydrogen, nitrogen, and sulfur atoms are represented by gray, white, light blue, and light orange ellipsoids, respectively.

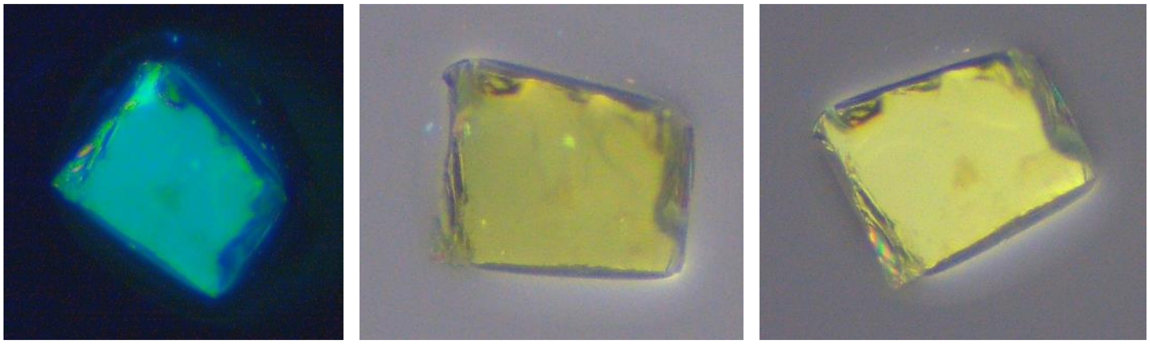

Crystal used for single crystal X-ray diffraction analysis.

Table S6. Crystal data and structure refinement for **8**.

Identification code	li19_04
Crystal Color	light yellow
Crystal Habit	blocky
Empirical formula	C38 H24 N2 S
Formula weight	540.65
Temperature	100(2) K

Wavelength	0.71073 Å
Crystal system	Monoclinic
Space group	$P2_1/n$
Unit cell dimensions	a = 8.6280(7) Å alpha = 90 °.
	b = 7.1962(6) Å beta = 99.1370(10) °.
	c = 22.0670(18) Å gamma = 90 °.
Volume	1352.73(19) $Å^3$
Z	2
Calculated density	1.327 Mg/m^3
Absorption coefficient	0.151 mm^{-1}
F(000)	564
Crystal size	0.270 x 0.230 x 0.190 mm
Theta range for data collection	1.869 to 27.215 °.
Limiting indices	-11<=h<=11, -9<=k<=9, -28<=l<=28
Reflections collected / unique	23523 / 3000 [R(int) = 0.0314]
Completeness to theta = 25.242°	99.9 %
Refinement method	Full-matrix least-squares on F^2
Data / restraints / parameters	3000 / 237 / 262
Goodness-of-fit on F^2	1.089
Final R indices [I>2sigma(I)]	R1 = 0.0408, wR2 = 0.1014
R indices (all data)	R1 = 0.0495, wR2 = 0.1069
Largest diff. peak and hole	0.324 and -0.286 e.$Å^{-3}$

Table S7. Atomic coordinates (x 10^4) and equivalent isotropic displacement parameters ($Å^2$ x 10^3) for li19_04. U(eq) is defined as one third of the trace of the orthogonalized Uij tensor.

	x	y	z	U(eq)

S(1)	7178(1)	1556(1)	5383(1)	34(1)
N(1)	5843(3)	1820(4)	4789(1)	25(1)
N(2)	7151(3)	3554(3)	5711(1)	30(1)
C(1)	5285(5)	3551(5)	4827(2)	17(1)
C(2)	4064(4)	4368(5)	4409(2)	15(1)
C(3)	3618(5)	6121(6)	4548(2)	21(1)
C(4)	4360(5)	7118(6)	5073(2)	24(1)
C(5)	5531(5)	6372(6)	5482(2)	22(1)
C(6)	6028(5)	4562(6)	5361(2)	22(1)
C(7)	3276(4)	3259(4)	3882(2)	15(1)
C(8)	1767(3)	2691(4)	3907(1)	18(1)
C(9)	963(4)	1435(4)	3481(2)	21(1)
C(10)	1707(8)	690(10)	3039(4)	23(1)
C(11)	3391(2)	1154(2)	2979(1)	28(1)
C(12)	4098(2)	559(2)	2477(1)	31(1)
C(13)	5526(2)	1211(2)	2397(1)	35(1)
C(14)	6299(2)	2531(2)	2805(1)	28(1)
C(15)	5662(1)	3192(2)	3296(1)	21(1)
C(16)	4190(2)	2456(2)	3411(1)	27(1)
C(17)	6498(1)	4780(2)	3640(1)	20(1)
C(18)	5897(2)	6570(2)	3554(1)	27(1)
C(19)	6716(2)	8080(2)	3833(1)	37(1)
C(20)	8148(2)	7830(2)	4198(1)	40(1)
C(21)	8770(2)	6074(2)	4283(1)	39(1)
C(22)	7955(1)	4554(2)	4006(1)	30(1)
C(23)	6354(4)	7412(5)	6029(2)	26(1)
C(24)	7767(4)	8216(6)	5974(2)	45(1)

C(25)	8586(4)	9355(6)	6438(2)	45(1)
C(26)	7918(8)	9724(11)	6940(4)	31(1)

Table S8. Bond lengths [Å] and angles [°] for li19_04.

S(1)-N(2)	1.611(3)
S(1)-N(1)	1.614(2)
N(1)-C(1)	1.342(4)
N(2)-C(6)	1.351(4)
C(1)-C(2)	1.413(4)
C(1)-C(6)	1.446(5)
C(2)-C(3)	1.368(4)
C(2)-C(7)	1.483(5)
C(3)-C(4)	1.426(6)
C(3)-H(3)	0.9500
C(4)-C(5)	1.355(5)
C(4)-H(4)	0.9500
C(5)-C(6)	1.410(5)
C(5)-C(23)	1.500(5)
C(7)-C(8)	1.375(4)
C(7)-C(16)	1.515(4)
C(8)-C(9)	1.406(4)
C(8)-H(8)	0.9500
C(9)-C(10)	1.360(8)
C(9)-H(9)	0.9500
C(10)-C(11)	1.517(8)

C(10)-H(10)	0.9500
C(11)-C(12)	1.4139(19)
C(11)-C(16)	1.4324(17)
C(12)-C(13)	1.355(2)
C(12)-H(12)	0.9500
C(13)-C(14)	1.4039(19)
C(13)-H(13)	0.9500
C(14)-C(15)	1.3737(17)
C(14)-H(14)	0.9500
C(15)-C(16)	1.4352(17)
C(15)-C(17)	1.4929(16)
C(17)-C(18)	1.3900(17)
C(17)-C(22)	1.3917(16)
C(18)-C(19)	1.3849(19)
C(18)-H(18)	0.9500
C(19)-C(20)	1.376(2)
C(19)-H(19)	0.9500
C(20)-C(21)	1.374(2)
C(20)-H(20)	0.9500
C(21)-C(22)	1.388(2)
C(21)-H(21)	0.9500
C(22)-H(22)	0.9500
C(23)-C(24)	1.372(5)
C(24)-C(25)	1.410(5)
C(24)-H(24)	0.9500
C(25)-C(26)	1.352(9)
C(25)-H(25)	0.9500

C(26)-H(26)	0.9500
N(2)-S(1)-N(1)	101.59(14)
C(1)-N(1)-S(1)	106.0(2)
C(6)-N(2)-S(1)	106.4(2)
N(1)-C(1)-C(2)	125.6(3)
N(1)-C(1)-C(6)	113.6(4)
C(2)-C(1)-C(6)	120.7(3)
C(3)-C(2)-C(1)	116.4(3)
C(3)-C(2)-C(7)	124.2(3)
C(1)-C(2)-C(7)	119.3(3)
C(2)-C(3)-C(4)	122.8(4)
C(2)-C(3)-H(3)	118.6
C(4)-C(3)-H(3)	118.6
C(5)-C(4)-C(3)	122.2(4)
C(5)-C(4)-H(4)	118.9
C(3)-C(4)-H(4)	118.9
C(4)-C(5)-C(6)	117.2(3)
C(4)-C(5)-C(23)	123.1(4)
C(6)-C(5)-C(23)	119.7(4)
N(2)-C(6)-C(5)	126.8(4)
N(2)-C(6)-C(1)	112.4(4)
C(5)-C(6)-C(1)	120.7(3)
C(8)-C(7)-C(2)	116.5(3)
C(8)-C(7)-C(16)	121.0(3)
C(2)-C(7)-C(16)	121.3(3)
C(7)-C(8)-C(9)	122.3(3)
C(7)-C(8)-H(8)	118.9

C(9)-C(8)-H(8)	118.9
C(10)-C(9)-C(8)	119.6(4)
C(10)-C(9)-H(9)	120.2
C(8)-C(9)-H(9)	120.2
C(9)-C(10)-C(11)	122.9(6)
C(9)-C(10)-H(10)	118.5
C(11)-C(10)-H(10)	118.5
C(12)-C(11)-C(16)	119.83(12)
C(12)-C(11)-C(10)	122.7(3)
C(16)-C(11)-C(10)	116.9(3)
C(13)-C(12)-C(11)	120.77(12)
C(13)-C(12)-H(12)	119.6
C(11)-C(12)-H(12)	119.6
C(12)-C(13)-C(14)	119.96(12)
C(12)-C(13)-H(13)	120.0
C(14)-C(13)-H(13)	120.0
C(15)-C(14)-C(13)	122.11(12)
C(15)-C(14)-H(14)	118.9
C(13)-C(14)-H(14)	118.9
C(14)-C(15)-C(16)	119.18(11)
C(14)-C(15)-C(17)	116.35(11)
C(16)-C(15)-C(17)	124.19(10)
C(11)-C(16)-C(15)	118.01(11)
C(11)-C(16)-C(7)	117.24(16)
C(15)-C(16)-C(7)	123.51(16)
C(18)-C(17)-C(22)	118.01(11)
C(18)-C(17)-C(15)	120.10(10)

C(22)-C(17)-C(15)	121.62(11)
C(19)-C(18)-C(17)	120.92(12)
C(19)-C(18)-H(18)	119.5
C(17)-C(18)-H(18)	119.5
C(20)-C(19)-C(18)	120.33(14)
C(20)-C(19)-H(19)	119.8
C(18)-C(19)-H(19)	119.8
C(21)-C(20)-C(19)	119.63(13)
C(21)-C(20)-H(20)	120.2
C(19)-C(20)-H(20)	120.2
C(20)-C(21)-C(22)	120.35(13)
C(20)-C(21)-H(21)	119.8
C(22)-C(21)-H(21)	119.8
C(21)-C(22)-C(17)	120.76(13)
C(21)-C(22)-H(22)	119.6
C(17)-C(22)-H(22)	119.6
C(24)-C(23)-C(5)	117.0(3)
C(23)-C(24)-C(25)	122.1(3)
C(23)-C(24)-H(24)	119.0
C(25)-C(24)-H(24)	119.0
C(26)-C(25)-C(24)	118.6(5)
C(26)-C(25)-H(25)	120.7
C(24)-C(25)-H(25)	120.7
C(11)#1-C(26)-C(25)	119.9(7)
C(11)#1-C(26)-H(26)	120.1
C(25)-C(26)-H(26)	120.1

Symmetry transformations used to generate equivalent atoms:#1 -x+1,-y+1,-z+1

Table S9. Anisotropic displacement parameters ($Å^2 \times 10^3$) for li19_04. The anisotropic displacement factor exponent takes the form: $-2 \pi^2 [h^2 a^{*2}U^{11} + ... + 2 h k a^* b^* U^{12}]$

	U11	U22	U33	U23	U13	U12
S(1)	35(1)	26(1)	34(1)	6(1)	-15(1)	-2(1)
N(1)	26(1)	21(1)	25(1)	3(1)	-5(1)	-2(1)
N(2)	29(1)	35(1)	24(1)	3(1)	-5(1)	-8(1)
C(1)	19(2)	16(2)	15(2)	5(1)	1(1)	-1(1)
C(2)	14(1)	19(2)	14(2)	2(1)	3(1)	0(1)
C(3)	19(2)	20(2)	23(2)	-4(2)	4(2)	0(1)
C(4)	24(2)	22(2)	27(2)	-5(2)	9(2)	-1(2)
C(5)	20(2)	28(2)	19(2)	-3(2)	7(1)	-6(1)
C(6)	21(2)	27(2)	18(2)	3(2)	2(1)	-3(2)
C(7)	18(1)	14(2)	13(1)	4(1)	-1(1)	4(1)
C(8)	18(1)	17(1)	17(1)	5(1)	0(1)	3(1)
C(9)	19(1)	19(1)	24(1)	7(1)	-4(1)	0(1)
C(10)	23(3)	18(2)	24(2)	3(2)	-8(2)	-4(2)
C(11)	41(1)	24(1)	19(1)	0(1)	-1(1)	-9(1)
C(12)	46(1)	21(1)	24(1)	-7(1)	-5(1)	5(1)
C(13)	41(1)	38(1)	27(1)	-11(1)	5(1)	13(1)
C(14)	27(1)	32(1)	26(1)	-2(1)	6(1)	6(1)
C(15)	24(1)	20(1)	18(1)	3(1)	2(1)	2(1)
C(16)	34(1)	30(1)	18(1)	-3(1)	5(1)	-12(1)
C(17)	19(1)	25(1)	17(1)	2(1)	6(1)	-2(1)

C(18)	26(1)	26(1)	26(1)	-2(1)	1(1)	1(1)
C(19)	49(1)	26(1)	35(1)	-2(1)	2(1)	-5(1)
C(20)	49(1)	41(1)	27(1)	1(1)	-1(1)	-25(1)
C(21)	27(1)	56(1)	31(1)	13(1)	-6(1)	-18(1)
C(22)	23(1)	35(1)	31(1)	12(1)	2(1)	-1(1)
C(23)	25(2)	29(2)	25(2)	-7(2)	7(1)	-10(1)
C(24)	41(2)	67(3)	33(2)	-23(2)	20(2)	-27(2)
C(25)	35(2)	60(3)	41(2)	-19(2)	14(2)	-30(2)
C(26)	33(3)	34(4)	26(2)	-11(2)	1(2)	-9(2)

Table S10. Hydrogen coordinates (x 10^4) and isotropic displacement parameters (Å^2 x 10^3) for li19_04.

	x	y	z	U(eq)
H(3)	2780	6698	4283	25
H(4)	4020	8348	5139	29
H(8)	1249	3164	4224	21
H(9)	-95	1110	3501	26
H(10)	1148	-160	2755	27
H(12)	3566	-307	2192	38
H(13)	6003	774	2064	42
H(14)	7294	2983	2741	34
H(18)	4912	6761	3302	32
H(19)	6287	9293	3771	45
H(20)	8704	8866	4391	47

H(21)	9762	5898	4532	47
H(22)	8397	3347	4066	36
H(24)	8209	7998	5613	54
H(25)	9585	9853	6400	54
H(26)	8382	10608	7233	38

(3)

Refinement Details

After data collection, the unit cell was re-determined using a subset of the full data collection. Intensity data were corrected for Lorentz, polarization, and background effects using the *CrysAlis^{Pro}* [3]. A numerical absorption correction was applied based on a Gaussian integration over a multifaceted crystal and followed by a semi-empirical correction for adsorption applied using the program *SCALE3 ABSPACK* [4]. The *SHELXL-2014* [5], series of programs was used for the solution and refinement of the crystal structure. The main structure was determined it was found that the crystal structure also contained a mixture of highly disordered DCM and hexane molecules in the interstitial space. This electron density was treated using the programs PLATON/SQUEEZE. The resulting analysis concluded that there was 115 electrons in the void space. However, due to the lack of any other empirical evidence this information was not added to the CIF file. Hydrogen atoms bound to carbon atoms were located in the difference Fourier map and were geometrically constrained using the appropriate AFIX commands.

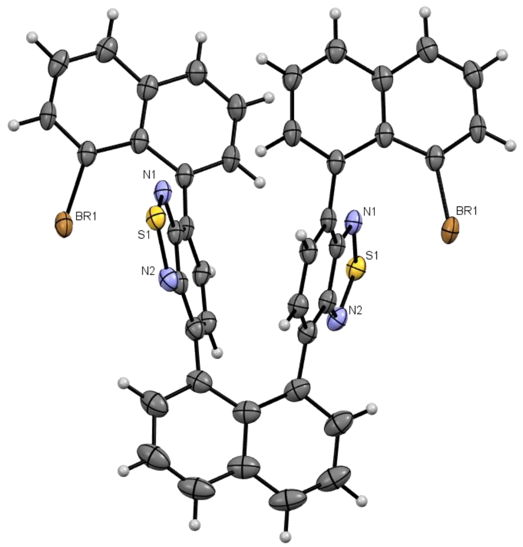

Figure S27. X-ray structure of **9**.

The thermal ellipsoids are represented at 50% probability. Carbon, hydrogen, nitrogen, sulfur and bromine atoms are represented by gray, white, light blue, light orange, and dark orange ellipsoids, respectively.

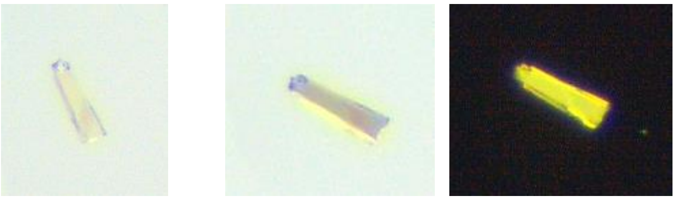

Crystal used for single crystal X-ray diffraction analysis.

Table S11. Crystal data and structure refinement for **9**.

Identification code	li19_23	
Crystal Color	light yellow	
Crystal Habit	needle	
Empirical formula	C42 H22 Br2 N4 S2	
Formula weight	806.57	
Temperature	100(2) K	
Wavelength	1.54178 Å	
Crystal system	Monoclinic	
Space group	*P*2/*c*	
Unit cell dimensions	a = 10.3171(3) Å	alpha = 90 °.
	b = 13.5577(3) Å	beta = 106.957(3) °.
	c = 14.8096(3) Å	gamma = 90 °.
Volume	1981.45(9) $Å^3$	
Z	2	
Calculated density	1.352 Mg/m^3	
Absorption coefficient	3.838 mm^{-1}	
F(000)	808	

Crystal size	0.171 x 0.044 x 0.037 mm
Theta range for data collection	3.260 to 77.618 °.
Limiting indices	-13<=h<=13, -17<=k<=17, -18<=l<=13
Reflections collected / unique	32415 / 4168 [R(int) = 0.0522]
Completeness to theta = 67.679°	99.8 %
Refinement method	Full-matrix least-squares on F^2
Data / restraints / parameters	4168 / 0 / 227
Goodness-of-fit on F^2	1.087
Final R indices [I>2sigma(I)]	R1 = 0.0433, wR2 = 0.1102
R indices (all data)	R1 = 0.0462, wR2 = 0.1117
Largest diff. peak and hole	0.796 and -1.111 e.Å^{-3}

Table S12. Atomic coordinates (x 10^4) and equivalent isotropic displacement parameters (Å^2 x 10^3) for li19_23. U(eq) is defined as one third of the trace of the orthogonalized Uij tensor.

	x	y	z	U(eq)
Br(1)	1047(1)	6078(1)	3830(1)	35(1)
S(1)	5432(1)	6378(1)	5045(1)	31(1)
N(1)	4466(2)	5700(2)	4216(2)	29(1)
N(2)	5223(3)	7443(2)	4536(2)	32(1)
C(1)	3923(3)	6306(2)	3488(2)	28(1)
C(2)	4359(3)	7309(2)	3667(2)	29(1)
C(3)	3939(3)	8044(2)	2953(2)	32(1)
C(4)	3098(3)	7734(2)	2099(2)	32(1)
C(5)	2648(3)	6745(2)	1926(2)	31(1)
C(6)	3014(3)	6016(2)	2597(2)	29(1)
C(7)	2620(3)	4968(2)	2400(2)	29(1)
C(8)	1995(3)	4373(2)	2968(2)	30(1)

C(9)	1404(3)	4718(2)	3671(2)	31(1)
C(10)	955(3)	4076(3)	4242(2)	34(1)
C(11)	1022(3)	3065(3)	4123(2)	38(1)
C(12)	1470(3)	2695(3)	3418(2)	36(1)
C(13)	1943(3)	3334(2)	2818(2)	33(1)
C(14)	2346(3)	2928(3)	2062(2)	36(1)
C(15)	2818(3)	3514(3)	1482(2)	37(1)
C(16)	2978(3)	4528(3)	1667(2)	35(1)
C(17)	4365(4)	9092(2)	3111(2)	37(1)
C(18)	5000	9596(3)	2500	38(1)
C(19)	5000	10644(4)	2500	45(1)
C(20)	4595(5)	11156(3)	3211(3)	55(1)
C(21)	4157(5)	10655(3)	3863(3)	55(1)
C(22)	3993(4)	9625(3)	3793(2)	47(1)

Table S13. Bond lengths [Å] and angles [°] for li19_23.

Br(1)-C(9)	1.909(3)
S(1)-N(2)	1.615(3)
S(1)-N(1)	1.622(3)
N(1)-C(1)	1.342(4)
N(2)-C(2)	1.347(4)
C(1)-C(2)	1.432(4)
C(1)-C(6)	1.433(4)
C(2)-C(3)	1.426(4)
C(3)-C(4)	1.374(4)

C(3)-C(17)	1.486(5)
C(4)-C(5)	1.418(5)
C(4)-H(4)	0.9500
C(5)-C(6)	1.374(4)
C(5)-H(5)	0.9500
C(6)-C(7)	1.483(4)
C(7)-C(16)	1.381(4)
C(7)-C(8)	1.446(4)
C(8)-C(13)	1.424(5)
C(8)-C(9)	1.430(4)
C(9)-C(10)	1.384(4)
C(10)-C(11)	1.386(5)
C(10)-H(10)	0.9500
C(11)-C(12)	1.355(5)
C(11)-H(11)	0.9500
C(12)-C(13)	1.425(4)
C(12)-H(12)	0.9500
C(13)-C(14)	1.416(4)
C(14)-C(15)	1.360(5)
C(14)-H(14)	0.9500
C(15)-C(16)	1.402(5)
C(15)-H(15)	0.9500
C(16)-H(16)	0.9500
C(17)-C(22)	1.384(5)
C(17)-C(18)	1.435(4)
C(18)-C(19)	1.421(7)
C(19)-C(20)#1	1.422(5)

C(19)-C(20)	1.422(5)
C(20)-C(21)	1.361(7)
C(20)-H(20)	0.9500
C(21)-C(22)	1.407(6)
C(21)-H(21)	0.9500
C(22)-H(22)	0.9500
N(2)-S(1)-N(1)	100.64(13)
C(1)-N(1)-S(1)	106.3(2)
C(2)-N(2)-S(1)	106.6(2)
N(1)-C(1)-C(2)	113.4(3)
N(1)-C(1)-C(6)	125.4(3)
C(2)-C(1)-C(6)	121.2(3)
N(2)-C(2)-C(3)	126.0(3)
N(2)-C(2)-C(1)	113.0(3)
C(3)-C(2)-C(1)	121.0(3)
C(4)-C(3)-C(2)	116.3(3)
C(4)-C(3)-C(17)	121.1(3)
C(2)-C(3)-C(17)	122.7(3)
C(3)-C(4)-C(5)	122.8(3)
C(3)-C(4)-H(4)	118.6
C(5)-C(4)-H(4)	118.6
C(6)-C(5)-C(4)	122.9(3)
C(6)-C(5)-H(5)	118.6
C(4)-C(5)-H(5)	118.6
C(5)-C(6)-C(1)	115.9(3)
C(5)-C(6)-C(7)	123.0(3)
C(1)-C(6)-C(7)	120.8(3)

C(16)-C(7)-C(8)	118.9(3)
C(16)-C(7)-C(6)	116.6(3)
C(8)-C(7)-C(6)	124.3(3)
C(13)-C(8)-C(9)	115.6(3)
C(13)-C(8)-C(7)	117.7(3)
C(9)-C(8)-C(7)	126.8(3)
C(10)-C(9)-C(8)	122.0(3)
C(10)-C(9)-Br(1)	114.9(2)
C(8)-C(9)-Br(1)	123.0(2)
C(9)-C(10)-C(11)	120.3(3)
C(9)-C(10)-H(10)	119.8
C(11)-C(10)-H(10)	119.8
C(12)-C(11)-C(10)	120.4(3)
C(12)-C(11)-H(11)	119.8
C(10)-C(11)-H(11)	119.8
C(11)-C(12)-C(13)	120.7(3)
C(11)-C(12)-H(12)	119.6
C(13)-C(12)-H(12)	119.6
C(14)-C(13)-C(8)	120.1(3)
C(14)-C(13)-C(12)	119.4(3)
C(8)-C(13)-C(12)	120.6(3)
C(15)-C(14)-C(13)	121.0(3)
C(15)-C(14)-H(14)	119.5
C(13)-C(14)-H(14)	119.5
C(14)-C(15)-C(16)	119.5(3)
C(14)-C(15)-H(15)	120.2
C(16)-C(15)-H(15)	120.2

C(7)-C(16)-C(15)	122.3(3)
C(7)-C(16)-H(16)	118.8
C(15)-C(16)-H(16)	118.8
C(22)-C(17)-C(18)	119.1(3)
C(22)-C(17)-C(3)	118.8(3)
C(18)-C(17)-C(3)	121.7(3)
C(19)-C(18)-C(17)	118.4(2)
C(19)-C(18)-C(17)#1	118.4(2)
C(17)-C(18)-C(17)#1	123.1(4)
C(18)-C(19)-C(20)#1	119.2(2)
C(18)-C(19)-C(20)	119.2(2)
C(20)#1-C(19)-C(20)	121.5(5)
C(21)-C(20)-C(19)	120.8(4)
C(21)-C(20)-H(20)	119.6
C(19)-C(20)-H(20)	119.6
C(20)-C(21)-C(22)	120.0(4)
C(20)-C(21)-H(21)	120.0
C(22)-C(21)-H(21)	120.0
C(17)-C(22)-C(21)	121.2(4)
C(17)-C(22)-H(22)	119.4
C(21)-C(22)-H(22)	119.4

Symmetry transformations used to generate equivalent atoms: #1 -x+1,y,-z+1/2

Table S14. Anisotropic displacement parameters ($Å^2 \times 10^3$) for li19_23. The anisotropic displacement factor exponent takes the form: $-2 \pi^2 [h^2 a^{*2}U^{11} + ... + 2 h k a^* b^* U^{12}]$

	U11	U22	U33	U23	U13	U12
Br(1)	33(1)	48(1)	26(1)	2(1)	15(1)	6(1)
S(1)	32(1)	41(1)	19(1)	-1(1)	6(1)	3(1)
N(1)	29(1)	41(1)	20(1)	0(1)	10(1)	2(1)
N(2)	36(1)	39(1)	22(1)	-2(1)	8(1)	5(1)
C(1)	24(1)	42(2)	18(1)	1(1)	9(1)	2(1)
C(2)	27(1)	39(2)	23(1)	-1(1)	9(1)	4(1)
C(3)	35(2)	38(2)	24(1)	0(1)	10(1)	3(1)
C(4)	31(2)	40(2)	22(1)	2(1)	6(1)	3(1)
C(5)	29(2)	46(2)	18(1)	2(1)	8(1)	-1(1)
C(6)	28(1)	42(2)	21(1)	1(1)	12(1)	0(1)
C(7)	26(1)	42(2)	21(1)	0(1)	7(1)	0(1)
C(8)	26(1)	45(2)	20(1)	0(1)	6(1)	-1(1)
C(9)	27(1)	45(2)	21(1)	1(1)	7(1)	0(1)
C(10)	28(2)	56(2)	21(1)	1(1)	12(1)	-2(1)
C(11)	33(2)	54(2)	26(1)	7(1)	8(1)	-8(1)
C(12)	32(2)	47(2)	29(2)	3(1)	7(1)	-5(1)
C(13)	27(1)	45(2)	24(1)	2(1)	4(1)	-1(1)
C(14)	35(2)	43(2)	29(2)	-3(1)	9(1)	-1(1)
C(15)	37(2)	49(2)	27(1)	-5(1)	14(1)	-1(1)
C(16)	33(2)	49(2)	25(1)	0(1)	14(1)	-2(1)
C(17)	44(2)	36(2)	26(2)	-1(1)	2(1)	7(1)
C(18)	46(3)	34(2)	27(2)	0	-1(2)	0
C(19)	52(3)	35(2)	36(2)	0	-7(2)	0
C(20)	69(3)	35(2)	43(2)	-7(2)	-11(2)	9(2)
C(21)	67(3)	48(2)	40(2)	-12(2)	0(2)	18(2)
C(22)	59(2)	47(2)	30(2)	-6(1)	6(2)	13(2)

Table S15. Hydrogen coordinates (x 10^4) and isotropic displacement parameters (Å^2 x 10^3) for li19_23.

	x	y	z	U(eq)
H(4)	2805	8202	1604	38
H(5)	2069	6579	1319	37
H(10)	600	4330	4719	41
H(11)	752	2630	4537	45
H(12)	1469	2002	3323	43
H(14)	2286	2236	1957	43
H(15)	3038	3238	955	44
H(16)	3346	4926	1274	42
H(20)	4631	11856	3231	66
H(21)	3962	11001	4366	66
H(22)	3619	9288	4221	56

(4)

Refinement Details

After data collection, the unit cell was re-determined using a subset of the full data collection. Intensity data were corrected for Lorentz, polarization, and background effects using the *CrysAlis^{Pro}* [3]. A numerical absorption correction was applied based on a Gaussian integration over a multifaceted crystal and followed by a semi-empirical correction for adsorption applied using the program *SCALE3 ABSPACK* [4]. The *SHELXL-2014* [5], series of programs was used for the solution and refinement of the crystal structure. The interstitial dichloromethane molecule was positionally disordered over two sites (A and B) with site occupancy values of 0.73 and 0.27 for the A and B sites, respectively. SIMU and DELU restraints were also applied to maintain reasonable ADP vales for chlorine atoms. Hydrogen atoms bound to carbon atoms were located in the difference Fourier map and were geometrically constrained using the appropriate AFIX commands.

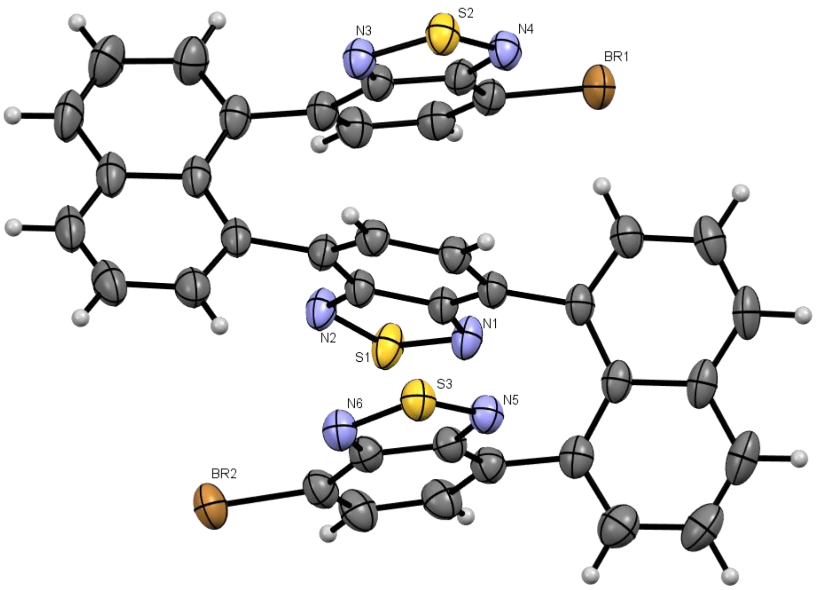

Figure S28. X-ray structure of **10**.

The thermal ellipsoids are represented at 50% probability. Carbon, hydrogen, nitrogen, sulfur, and bromine atoms are represented by gray, white, light blue, yellow, and light orange ellipsoids, respectively. The interstitial DCM molecule was omitted for clarity.

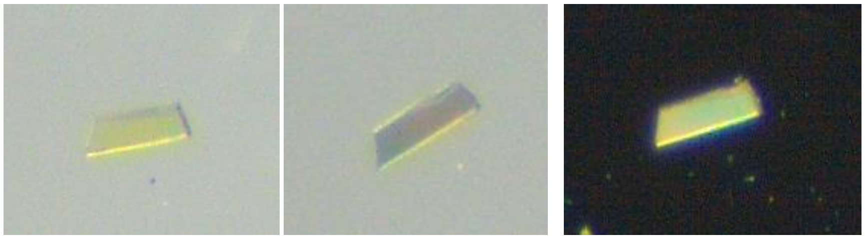

Crystal used for single crystal X-ray diffraction analysis.

Table S16. Crystal data and structure refinement for **10**.

Identification code	li19_14
Crystal Color	pale yellow
Crystal Habit	prism
Empirical formula	C39 H20 Br2 Cl2 N6 S3
Formula weight	899.51
Temperature	100(2) K

Wavelength	1.54178 Å
Crystal system	Triclinic
Space group	$P\bar{1}$
Unit cell dimensions	a = 10.6083(2) Å alpha = 67.156(2) °.
	b = 12.0712(3) Å beta = 80.895(2) °.
	c = 14.9537(2) Å gamma = 86.742(2) °.
Volume	1742.44(6) $Å^3$
Z	2
Calculated density	1.714 Mg/m^3
Absorption coefficient	6.376 mm^{-1}
F(000)	896
Crystal size	0.175 x 0.050 x 0.041 mm
Theta range for data collection	3.243 to 77.455 °.
Limiting indices	-13<=h<=13, -15<=k<=15, -18<=l<=17
Reflections collected / unique	92517 / 7242 [R(int) = 0.0548]
Completeness to theta = 67.679°	100.0 %
Refinement method	Full-matrix least-squares on F^2
Data / restraints / parameters	7242 / 12 / 488
Goodness-of-fit on F^2	1.053
Final R indices [I>2sigma(I)]	R1 = 0.0366, wR2 = 0.1013
R indices (all data)	R1 = 0.0394, wR2 = 0.1035
Largest diff. peak and hole	0.583 and -0.655 e.$Å^{-3}$

Table S17. Atomic coordinates (x 10^4) and equivalent isotropic displacement parameters ($Å^2$ x 10^3) for li19_14. U(eq) is defined as one third of the trace of the orthogonalized Uij tensor.

	x	y	z	U(eq)
Br(1)	615(1)	7529(1)	4681(1)	51(1)

Br(2)	8875(1)	8712(1)	-123(1)	50(1)
Cl(1A)	933(7)	1299(4)	2544(4)	121(2)
Cl(1B)	1195(9)	1282(8)	2496(5)	61(2)
Cl(2A)	107(4)	3673(4)	2421(4)	88(1)
Cl(2B)	-309(14)	3162(17)	2757(9)	109(3)
S(1)	4123(1)	10175(1)	1367(1)	45(1)
S(2)	2588(1)	4163(1)	4089(1)	43(1)
S(3)	8600(1)	5876(1)	3235(1)	41(1)
N(1)	4325(2)	9512(2)	2503(2)	41(1)
N(2)	4393(2)	9090(2)	988(2)	42(1)
N(3)	2722(2)	4826(2)	2907(2)	41(1)
N(4)	1941(2)	5219(2)	4425(2)	40(1)
N(5)	7976(2)	6838(2)	3674(2)	38(1)
N(6)	8729(2)	6670(2)	2064(2)	42(1)
C(1)	4647(2)	8368(2)	2632(2)	34(1)
C(2)	4672(2)	8125(2)	1767(2)	35(1)
C(3)	4960(2)	6962(2)	1756(2)	36(1)
C(4)	5259(2)	6105(2)	2618(2)	38(1)
C(5)	5240(2)	6345(2)	3475(2)	36(1)
C(6)	4968(2)	7453(2)	3518(2)	35(1)
C(7)	5026(3)	6732(2)	843(2)	39(1)
C(8)	3935(3)	6673(2)	408(2)	40(1)
C(9)	2630(3)	6707(2)	831(2)	42(1)
C(10)	1661(3)	6617(3)	351(2)	54(1)
C(11)	1920(4)	6523(3)	-574(2)	63(1)
C(12)	3127(3)	6486(3)	-986(2)	57(1)
C(13)	4171(3)	6547(2)	-521(2)	46(1)

C(14)	5429(3)	6439(2)	-949(2)	51(1)
C(15)	6436(3)	6436(3)	-496(2)	54(1)
C(16)	6234(3)	6586(2)	401(2)	47(1)
C(17)	2200(2)	6868(2)	1771(2)	39(1)
C(18)	2261(2)	5946(2)	2718(2)	36(1)
C(19)	1811(2)	6154(2)	3592(2)	36(1)
C(20)	1247(2)	7279(2)	3523(2)	38(1)
C(21)	1144(2)	8128(2)	2620(2)	42(1)
C(22)	1619(2)	7914(2)	1765(2)	42(1)
C(23)	4931(2)	7641(2)	4447(2)	36(1)
C(24)	5796(2)	8391(2)	4611(2)	38(1)
C(25)	6870(2)	9040(2)	3927(2)	41(1)
C(26)	7598(3)	9753(3)	4161(2)	54(1)
C(27)	7350(3)	9873(3)	5074(3)	58(1)
C(28)	6379(3)	9248(3)	5748(2)	54(1)
C(29)	5584(3)	8493(2)	5548(2)	44(1)
C(30)	4581(3)	7854(3)	6274(2)	50(1)
C(31)	3799(3)	7118(3)	6111(2)	50(1)
C(32)	3977(3)	7016(2)	5199(2)	44(1)
C(33)	7295(2)	8944(2)	2958(2)	39(1)
C(34)	7832(2)	7860(2)	2899(2)	36(1)
C(35)	8275(2)	7761(2)	1979(2)	38(1)
C(36)	8231(2)	8801(2)	1100(2)	43(1)
C(37)	7754(3)	9833(2)	1164(2)	48(1)
C(38)	7297(3)	9911(2)	2088(2)	47(1)
C(39)	1145(4)	2462(4)	2901(3)	78(1)

Table S18. Bond lengths [Å] and angles [°] for li19_14.

Br(1)-C(20)	1.886(2)
Br(2)-C(36)	1.889(3)
Cl(1A)-C(39)	1.721(6)
Cl(1B)-C(39)	1.747(8)
Cl(2A)-C(39)	1.765(5)
Cl(2B)-C(39)	1.719(10)
S(1)-N(2)	1.612(2)
S(1)-N(1)	1.617(2)
S(2)-N(3)	1.619(2)
S(2)-N(4)	1.623(2)
S(3)-N(5)	1.608(2)
S(3)-N(6)	1.625(2)
N(1)-C(1)	1.349(3)
N(2)-C(2)	1.348(3)
N(3)-C(18)	1.349(3)
N(4)-C(19)	1.338(3)
N(5)-C(34)	1.346(3)
N(6)-C(35)	1.342(3)
C(1)-C(2)	1.430(3)
C(1)-C(6)	1.434(3)
C(2)-C(3)	1.426(3)
C(3)-C(4)	1.374(3)
C(3)-C(7)	1.486(3)
C(4)-C(5)	1.416(3)
C(4)-H(4)	0.9500

C(5)-C(6)	1.376(3)
C(5)-H(5)	0.9500
C(6)-C(23)	1.487(3)
C(7)-C(16)	1.380(4)
C(7)-C(8)	1.433(4)
C(8)-C(9)	1.434(4)
C(8)-C(13)	1.437(3)
C(9)-C(10)	1.374(4)
C(9)-C(17)	1.489(3)
C(10)-C(11)	1.415(4)
C(10)-H(10)	0.9500
C(11)-C(12)	1.337(5)
C(11)-H(11)	0.9500
C(12)-C(13)	1.417(4)
C(12)-H(12)	0.9500
C(13)-C(14)	1.408(4)
C(14)-C(15)	1.352(4)
C(14)-H(14)	0.9500
C(15)-C(16)	1.404(4)
C(15)-H(15)	0.9500
C(16)-H(16)	0.9500
C(17)-C(22)	1.371(3)
C(17)-C(18)	1.430(3)
C(18)-C(19)	1.430(3)
C(19)-C(20)	1.425(3)
C(20)-C(21)	1.358(4)
C(21)-C(22)	1.412(4)

C(21)-H(21)	0.9500
C(22)-H(22)	0.9500
C(23)-C(32)	1.387(4)
C(23)-C(24)	1.434(3)
C(24)-C(29)	1.436(3)
C(24)-C(25)	1.438(4)
C(25)-C(26)	1.362(4)
C(25)-C(33)	1.495(3)
C(26)-C(27)	1.411(4)
C(26)-H(26)	0.9500
C(27)-C(28)	1.348(5)
C(27)-H(27)	0.9500
C(28)-C(29)	1.419(4)
C(28)-H(28)	0.9500
C(29)-C(30)	1.409(4)
C(30)-C(31)	1.361(4)
C(30)-H(30)	0.9500
C(31)-C(32)	1.399(4)
C(31)-H(31)	0.9500
C(32)-H(32)	0.9500
C(33)-C(38)	1.369(4)
C(33)-C(34)	1.426(3)
C(34)-C(35)	1.429(3)
C(35)-C(36)	1.427(3)
C(36)-C(37)	1.350(4)
C(37)-C(38)	1.428(4)
C(37)-H(37)	0.9500

C(38)-H(38)	0.9500
C(39)-H(39A)	0.9900
C(39)-H(39B)	0.9900
C(39)-H(39C)	0.9900
C(39)-H(39D)	0.9900
N(2)-S(1)-N(1)	101.34(10)
N(3)-S(2)-N(4)	101.03(11)
N(5)-S(3)-N(6)	101.03(11)
C(1)-N(1)-S(1)	106.03(16)
C(2)-N(2)-S(1)	105.99(16)
C(18)-N(3)-S(2)	106.37(17)
C(19)-N(4)-S(2)	105.53(17)
C(34)-N(5)-S(3)	106.52(17)
C(35)-N(6)-S(3)	105.74(17)
N(1)-C(1)-C(2)	113.1(2)
N(1)-C(1)-C(6)	126.1(2)
C(2)-C(1)-C(6)	120.7(2)
N(2)-C(2)-C(3)	124.6(2)
N(2)-C(2)-C(1)	113.5(2)
C(3)-C(2)-C(1)	121.9(2)
C(4)-C(3)-C(2)	115.8(2)
C(4)-C(3)-C(7)	122.7(2)
C(2)-C(3)-C(7)	121.4(2)
C(3)-C(4)-C(5)	122.4(2)
C(3)-C(4)-H(4)	118.8
C(5)-C(4)-H(4)	118.8
C(6)-C(5)-C(4)	123.8(2)

C(6)-C(5)-H(5)	118.1
C(4)-C(5)-H(5)	118.1
C(5)-C(6)-C(1)	115.3(2)
C(5)-C(6)-C(23)	121.3(2)
C(1)-C(6)-C(23)	123.3(2)
C(16)-C(7)-C(8)	119.8(2)
C(16)-C(7)-C(3)	116.0(2)
C(8)-C(7)-C(3)	124.1(2)
C(7)-C(8)-C(9)	125.4(2)
C(7)-C(8)-C(13)	117.1(2)
C(9)-C(8)-C(13)	117.5(2)
C(10)-C(9)-C(8)	120.0(2)
C(10)-C(9)-C(17)	114.8(2)
C(8)-C(9)-C(17)	125.1(2)
C(9)-C(10)-C(11)	121.4(3)
C(9)-C(10)-H(10)	119.3
C(11)-C(10)-H(10)	119.3
C(12)-C(11)-C(10)	119.9(3)
C(12)-C(11)-H(11)	120.0
C(10)-C(11)-H(11)	120.0
C(11)-C(12)-C(13)	121.6(3)
C(11)-C(12)-H(12)	119.2
C(13)-C(12)-H(12)	119.2
C(14)-C(13)-C(12)	120.2(2)
C(14)-C(13)-C(8)	120.2(3)
C(12)-C(13)-C(8)	119.6(3)
C(15)-C(14)-C(13)	121.2(2)

C(15)-C(14)-H(14)	119.4
C(13)-C(14)-H(14)	119.4
C(14)-C(15)-C(16)	119.7(3)
C(14)-C(15)-H(15)	120.2
C(16)-C(15)-H(15)	120.2
C(7)-C(16)-C(15)	121.8(3)
C(7)-C(16)-H(16)	119.1
C(15)-C(16)-H(16)	119.1
C(22)-C(17)-C(18)	115.8(2)
C(22)-C(17)-C(9)	120.2(2)
C(18)-C(17)-C(9)	123.8(2)
N(3)-C(18)-C(17)	126.6(2)
N(3)-C(18)-C(19)	112.5(2)
C(17)-C(18)-C(19)	120.9(2)
N(4)-C(19)-C(20)	125.7(2)
N(4)-C(19)-C(18)	114.6(2)
C(20)-C(19)-C(18)	119.7(2)
C(21)-C(20)-C(19)	118.8(2)
C(21)-C(20)-Br(1)	121.83(19)
C(19)-C(20)-Br(1)	119.38(18)
C(20)-C(21)-C(22)	120.6(2)
C(20)-C(21)-H(21)	119.7
C(22)-C(21)-H(21)	119.7
C(17)-C(22)-C(21)	124.0(2)
C(17)-C(22)-H(22)	118.0
C(21)-C(22)-H(22)	118.0
C(32)-C(23)-C(24)	119.5(2)

C(32)-C(23)-C(6)	115.2(2)
C(24)-C(23)-C(6)	125.2(2)
C(23)-C(24)-C(29)	117.2(2)
C(23)-C(24)-C(25)	126.1(2)
C(29)-C(24)-C(25)	116.7(2)
C(26)-C(25)-C(24)	120.4(2)
C(26)-C(25)-C(33)	116.0(3)
C(24)-C(25)-C(33)	123.5(2)
C(25)-C(26)-C(27)	122.1(3)
C(25)-C(26)-H(26)	119.0
C(27)-C(26)-H(26)	119.0
C(28)-C(27)-C(26)	119.4(3)
C(28)-C(27)-H(27)	120.3
C(26)-C(27)-H(27)	120.3
C(27)-C(28)-C(29)	121.2(3)
C(27)-C(28)-H(28)	119.4
C(29)-C(28)-H(28)	119.4
C(30)-C(29)-C(28)	119.3(2)
C(30)-C(29)-C(24)	120.5(2)
C(28)-C(29)-C(24)	120.2(3)
C(31)-C(30)-C(29)	121.1(2)
C(31)-C(30)-H(30)	119.4
C(29)-C(30)-H(30)	119.4
C(30)-C(31)-C(32)	119.4(3)
C(30)-C(31)-H(31)	120.3
C(32)-C(31)-H(31)	120.3
C(23)-C(32)-C(31)	122.2(3)

C(23)-C(32)-H(32)	118.9
C(31)-C(32)-H(32)	118.9
C(38)-C(33)-C(34)	116.5(2)
C(38)-C(33)-C(25)	122.2(2)
C(34)-C(33)-C(25)	121.1(2)
N(5)-C(34)-C(33)	125.1(2)
N(5)-C(34)-C(35)	113.0(2)
C(33)-C(34)-C(35)	121.9(2)
N(6)-C(35)-C(36)	127.7(2)
N(6)-C(35)-C(34)	113.8(2)
C(36)-C(35)-C(34)	118.5(2)
C(37)-C(36)-C(35)	119.1(2)
C(37)-C(36)-Br(2)	121.5(2)
C(35)-C(36)-Br(2)	119.37(19)
C(36)-C(37)-C(38)	121.7(2)
C(36)-C(37)-H(37)	119.2
C(38)-C(37)-H(37)	119.2
C(33)-C(38)-C(37)	122.2(2)
C(33)-C(38)-H(38)	118.9
C(37)-C(38)-H(38)	118.9
Cl(2B)-C(39)-Cl(1B)	108.7(6)
Cl(1A)-C(39)-Cl(2A)	112.1(3)
Cl(1A)-C(39)-H(39A)	109.2
Cl(2A)-C(39)-H(39A)	109.2
Cl(1A)-C(39)-H(39B)	109.2
Cl(2A)-C(39)-H(39B)	109.2
H(39A)-C(39)-H(39B)	107.9

Cl(2B)-C(39)-H(39C)	110.0
Cl(1B)-C(39)-H(39C)	110.0
Cl(2B)-C(39)-H(39D)	110.0
Cl(1B)-C(39)-H(39D)	110.0
H(39C)-C(39)-H(39D)	108.3

Symmetry transformations used to generate equivalent atoms: #1 -x+1,y,-z+1/2

Table S19. Anisotropic displacement parameters ($Å^2 \times 10^3$) for li19_14. The anisotropic displacement factor exponent takes the form: $-2 \pi^2 [h^2 a^{*2}U^{11} + ... + 2 h k a^* b^* U^{12}]$

	U11	U22	U33	U23	U13	U12
Br(1)	57(1)	59(1)	43(1)	-30(1)	-1(1)	1(1)
Br(2)	60(1)	54(1)	31(1)	-11(1)	-2(1)	2(1)
Cl(1A)	165(4)	86(2)	128(3)	-63(2)	2(2)	-22(2)
Cl(1B)	85(3)	66(3)	51(2)	-43(2)	-14(2)	4(2)
Cl(2A)	94(2)	93(2)	107(2)	-65(2)	-44(2)	23(1)
Cl(2B)	117(6)	146(7)	99(5)	-82(5)	-46(4)	52(5)
S(1)	74(1)	33(1)	31(1)	-12(1)	-18(1)	7(1)
S(2)	60(1)	34(1)	32(1)	-9(1)	-10(1)	4(1)
S(3)	50(1)	37(1)	36(1)	-13(1)	-9(1)	7(1)
N(1)	59(1)	37(1)	30(1)	-15(1)	-13(1)	8(1)
N(2)	67(1)	33(1)	27(1)	-11(1)	-12(1)	0(1)
N(3)	55(1)	36(1)	31(1)	-12(1)	-9(1)	2(1)
N(4)	51(1)	38(1)	30(1)	-11(1)	-6(1)	0(1)
N(5)	44(1)	35(1)	33(1)	-11(1)	-8(1)	3(1)
N(6)	49(1)	42(1)	35(1)	-15(1)	-6(1)	4(1)
C(1)	44(1)	33(1)	27(1)	-12(1)	-6(1)	2(1)
C(2)	47(1)	34(1)	26(1)	-11(1)	-6(1)	1(1)

C(3)	47(1)	35(1)	29(1)	-15(1)	-5(1)	-2(1)
C(4)	45(1)	33(1)	35(1)	-14(1)	-5(1)	1(1)
C(5)	45(1)	33(1)	28(1)	-8(1)	-8(1)	2(1)
C(6)	41(1)	37(1)	26(1)	-12(1)	-6(1)	1(1)
C(7)	58(1)	32(1)	27(1)	-13(1)	-1(1)	-2(1)
C(8)	62(2)	32(1)	24(1)	-10(1)	-6(1)	1(1)
C(9)	60(1)	37(1)	28(1)	-11(1)	-12(1)	7(1)
C(10)	61(2)	64(2)	40(2)	-23(1)	-18(1)	9(1)
C(11)	80(2)	74(2)	43(2)	-25(2)	-28(2)	6(2)
C(12)	92(2)	55(2)	27(1)	-17(1)	-18(1)	3(2)
C(13)	77(2)	36(1)	26(1)	-12(1)	-5(1)	-1(1)
C(14)	83(2)	43(1)	29(1)	-17(1)	2(1)	-3(1)
C(15)	72(2)	46(1)	40(2)	-20(1)	11(1)	-1(1)
C(16)	59(2)	44(1)	40(1)	-19(1)	1(1)	-5(1)
C(17)	46(1)	40(1)	29(1)	-12(1)	-8(1)	2(1)
C(18)	42(1)	36(1)	31(1)	-12(1)	-7(1)	2(1)
C(19)	44(1)	37(1)	30(1)	-13(1)	-7(1)	-2(1)
C(20)	42(1)	39(1)	35(1)	-17(1)	-5(1)	0(1)
C(21)	46(1)	37(1)	44(1)	-16(1)	-7(1)	4(1)
C(22)	51(1)	36(1)	34(1)	-8(1)	-9(1)	5(1)
C(23)	47(1)	37(1)	24(1)	-10(1)	-10(1)	7(1)
C(24)	50(1)	35(1)	30(1)	-14(1)	-12(1)	10(1)
C(25)	52(1)	36(1)	36(1)	-15(1)	-11(1)	9(1)
C(26)	60(2)	47(2)	58(2)	-24(1)	-14(1)	3(1)
C(27)	71(2)	52(2)	67(2)	-36(2)	-28(2)	8(1)
C(28)	77(2)	56(2)	47(2)	-33(1)	-29(2)	21(1)
C(29)	62(2)	44(1)	34(1)	-20(1)	-20(1)	18(1)

C(30)	70(2)	56(2)	25(1)	-19(1)	-12(1)	19(1)
C(31)	64(2)	52(2)	27(1)	-9(1)	-3(1)	9(1)
C(32)	52(1)	45(1)	30(1)	-11(1)	-6(1)	3(1)
C(33)	43(1)	37(1)	36(1)	-14(1)	-5(1)	0(1)
C(34)	40(1)	35(1)	31(1)	-9(1)	-7(1)	0(1)
C(35)	39(1)	39(1)	33(1)	-13(1)	-5(1)	1(1)
C(36)	45(1)	47(1)	31(1)	-9(1)	-3(1)	-1(1)
C(37)	57(2)	39(1)	36(1)	-4(1)	-3(1)	1(1)
C(38)	54(1)	35(1)	43(2)	-9(1)	-4(1)	1(1)
C(39)	98(3)	82(2)	66(2)	-43(2)	-13(2)	6(2)

Table S20. Hydrogen coordinates (x 10^4) and isotropic displacement parameters (Å^2 x 10^3) for li19_14.

	x	y	z	U(eq)
H(4)	5486	5323	2639	45
H(5)	5427	5704	4053	43
H(10)	800	6618	647	64
H(11)	1236	6486	-902	75
H(12)	3289	6417	-1605	68
H(14)	5573	6367	-1567	62
H(15)	7277	6334	-783	64
H(16)	6948	6585	713	57
H(21)	748	8874	2563	51
H(22)	1530	8532	1148	51
H(26)	8296	10183	3694	64
H(27)	7863	10391	5212	69

H(28)	6225	9314	6369	65
H(30)	4448	7938	6888	60
H(31)	3138	6677	6612	60
H(32)	3426	6502	5090	52
H(37)	7722	10525	579	57
H(38)	6983	10659	2100	56
H(39A)	2039	2750	2676	93
H(39B)	993	2163	3627	93
H(39C)	1260	2148	3601	93
H(39D)	1841	3043	2516	93

(5)

Refinement Details

After data collection, the unit cell was re-determined using a subset of the full data collection. Intensity data were corrected for Lorentz, polarization, and background effects using the *CrysAlis*^{Pro} [3]. A numerical absorption correction was applied based on a Gaussian integration over a multifaceted crystal and followed by a semi-empirical correction for adsorption applied using the program *SCALE3 ABSPACK* [4]. The *SHELXL-2014* [5], series of programs was used for the solution and refinement of the crystal structure. The chlorine atom Cl4 was positionally disordered about a mirror plane and its site occupancy was set to 0.5. Hydrogen atoms bound to carbon atoms were located in the difference Fourier map and were geometrically constrained using the appropriate AFIX commands.

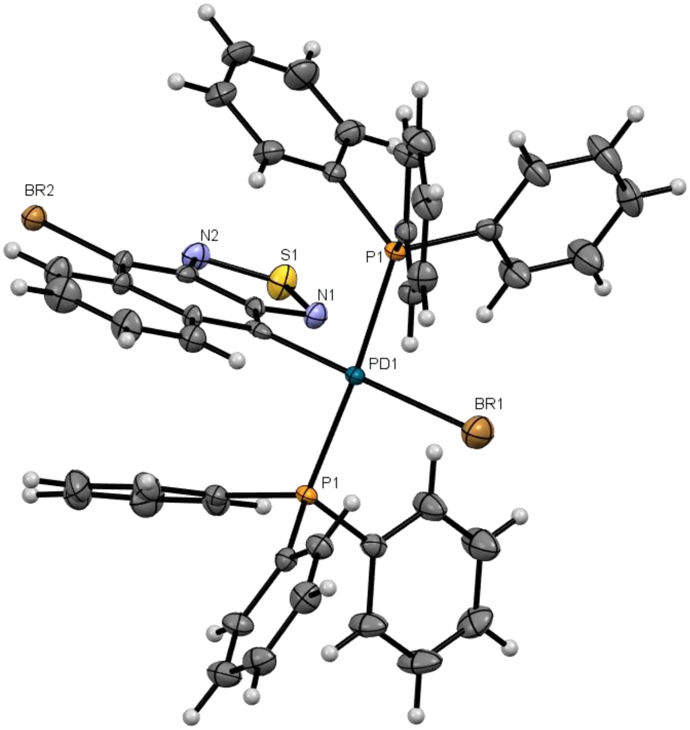

Figure S29. X-ray structure of **11**.

The thermal ellipsoids are represented at 50% probability. Carbon, hydrogen, nitrogen, phosphorus, sulfur, bromine, and palladium atoms are represented by gray, white, light blue, light orange, yellow, dark orange, and teal ellipsoids, respectively. The interstitial dichloromethane molecules were omitted for clarity.

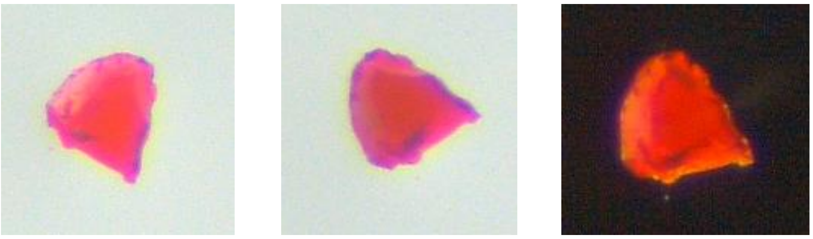

Crystal used for single crystal X-ray diffraction analysis.

Table S21. Crystal data and structure refinement for **11**.

Identification code	li19_34
Crystal Color	dark orange
Crystal Habit	irregular
Empirical formula	C48 H38 Br2 Cl4 N2 P2 Pd S

Formula weight	1144.82
Temperature	100(2) K
Wavelength	1.54178 Å
Crystal system	Monoclinic
Space group	*Im*
Unit cell dimensions	a = 9.76620(10) Å alpha = 90 °.
	b = 15.25770(10) Å beta = 96.8790(10) °.
	c = 15.4858(2) Å gamma = 90 °.
Volume	2290.92(4) Å^3
Z	2
Calculated density	1.660 Mg/m^3
Absorption coefficient	8.833 mm^{-1}
F(000)	1140
Crystal size	0.164 x 0.150 x 0.130 mm
Theta range for data collection	4.082 to 77.227 °.
Limiting indices	-12<=h<=12, -17<=k<=19, -19<=l<=19
Reflections collected / unique	22188 / 4511 [R(int) = 0.0324]
Completeness to theta = 67.679°	100.0 %
Refinement method	Full-matrix least-squares on F^2
Data / restraints / parameters	4511 / 26 / 307
Goodness-of-fit on F^2	1.031
Final R indices [I>2sigma(I)]	R1 = 0.0350, wR2 = 0.0983
R indices (all data)	R1 = 0.0351, wR2 = 0.0985
Absolute structure parameter	0.011(4)
Largest diff. peak and hole	0.863 and -1.580 e.Å^{-3}

Table S22. Atomic coordinates (x 10^4) and equivalent isotropic displacement parameters ($Å^2$ x 10^3) for li19_34. U(eq) is defined as one third of the trace of the orthogonalized Uij tensor.

	x	y	z	U(eq)
Pd(1)	4309(1)	5000	4656(1)	14(1)
Br(1)	5543(1)	5000	6150(1)	40(1)
Br(2)	453(1)	5000	728(1)	24(1)
Cl(1)	10036(5)	5000	7937(3)	60(1)
Cl(2)	9776(4)	5000	6034(2)	49(1)
Cl(3)	4543(4)	5000	8547(3)	66(1)
Cl(4)	7355(6)	4684(5)	9338(4)	81(3)
S(1)	-546(3)	5000	3661(2)	30(1)
P(1)	4225(2)	3475(1)	4606(1)	15(1)
N(1)	1017(9)	5000	4102(6)	23(2)
N(2)	-419(10)	5000	2636(6)	25(2)
C(1)	1802(10)	5000	3396(6)	17(2)
C(2)	965(10)	5000	2564(6)	19(2)
C(3)	1601(11)	5000	1816(6)	19(2)
C(4)	3043(10)	5000	1846(6)	17(2)
C(5)	3704(12)	5000	1096(7)	28(2)
C(6)	5145(11)	5000	1189(9)	35(3)
C(7)	5929(12)	5000	1969(8)	30(2)
C(8)	5324(12)	5000	2740(8)	25(2)
C(9)	3854(9)	5000	2700(6)	16(2)
C(10)	3255(10)	5000	3483(7)	18(2)
C(11)	5894(7)	2947(5)	4587(4)	19(1)
C(12)	5996(7)	2068(5)	4320(5)	24(1)
C(13)	7273(8)	1666(5)	4330(6)	32(2)

C(14)	8474(8)	2129(5)	4618(5)	31(2)
C(15)	8387(7)	2994(5)	4864(5)	29(2)
C(16)	7110(7)	3411(5)	4856(5)	22(1)
C(17)	3490(6)	3024(4)	5545(4)	20(1)
C(18)	3695(9)	2154(6)	5790(6)	35(2)
C(19)	3120(10)	1829(6)	6502(7)	43(2)
C(20)	2349(9)	2346(6)	6962(5)	37(2)
C(21)	2139(10)	3203(6)	6727(6)	44(2)
C(22)	2709(10)	3540(6)	6017(6)	35(2)
C(23)	3169(7)	2977(4)	3674(4)	20(1)
C(24)	3691(8)	2909(5)	2884(5)	29(2)
C(25)	2909(11)	2550(5)	2166(5)	38(2)
C(26)	1592(11)	2251(6)	2234(6)	39(2)
C(27)	1047(9)	2347(6)	3012(6)	39(2)
C(28)	1838(8)	2698(5)	3732(5)	28(2)
C(29)	8980(20)	5000	7032(11)	68(5)
C(30)	6328(16)	5000	8415(11)	51(4)

Table S23. Bond lengths [Å] and angles [°] for li19_34.

Pd(1)-C(10)	1.978(11)
Pd(1)-P(1)#1	2.3291(13)
Pd(1)-P(1)	2.3292(13)
Pd(1)-Br(1)	2.4776(12)
Br(2)-C(3)	1.909(10)
Cl(1)-C(29)	1.638(18)
Cl(2)-C(29)	1.81(2)

Cl(3)-C(30)	1.779(16)
Cl(4)-C(30)	1.714(17)
Cl(4)-H(30B)#1	1.808(17)
S(1)-N(1)	1.596(9)
S(1)-N(2)	1.607(10)
P(1)-C(11)	1.821(7)
P(1)-C(17)	1.830(7)
P(1)-C(23)	1.836(7)
N(1)-C(1)	1.409(13)
N(2)-C(2)	1.369(14)
C(1)-C(10)	1.410(14)
C(1)-C(2)	1.441(14)
C(2)-C(3)	1.378(15)
C(3)-C(4)	1.403(14)
C(4)-C(5)	1.395(16)
C(4)-C(9)	1.458(13)
C(5)-C(6)	1.397(15)
C(5)-H(5)	0.9500
C(6)-C(7)	1.351(18)
C(6)-H(6)	0.9500
C(7)-C(8)	1.395(16)
C(7)-H(7)	0.9500
C(8)-C(9)	1.430(14)
C(8)-H(8)	0.9500
C(9)-C(10)	1.407(14)
C(11)-C(16)	1.402(10)
C(11)-C(12)	1.411(10)

C(12)-C(13)	1.388(11)
C(12)-H(12)	0.9500
C(13)-C(14)	1.395(12)
C(13)-H(13)	0.9500
C(14)-C(15)	1.380(12)
C(14)-H(14)	0.9500
C(15)-C(16)	1.399(10)
C(15)-H(15)	0.9500
C(16)-H(16)	0.9500
C(17)-C(22)	1.368(11)
C(17)-C(18)	1.389(11)
C(18)-C(19)	1.388(12)
C(18)-H(18)	0.9500
C(19)-C(20)	1.351(13)
C(19)-H(19)	0.9500
C(20)-C(21)	1.367(13)
C(20)-H(20)	0.9500
C(21)-C(22)	1.390(12)
C(21)-H(21)	0.9500
C(22)-H(22)	0.9500
C(23)-C(28)	1.381(10)
C(23)-C(24)	1.384(11)
C(24)-C(25)	1.385(11)
C(24)-H(24)	0.9500
C(25)-C(26)	1.380(15)
C(25)-H(25)	0.9500
C(26)-C(27)	1.383(14)

C(26)-H(26)	0.9500
C(27)-C(28)	1.386(11)
C(27)-H(27)	0.9500
C(28)-H(28)	0.9500
C(29)-H(29A)	0.9900
C(29)-H(29B)	0.9900
C(30)-H(30A)	0.9900
C(30)-H(30B)	0.9900
C(30)-H(30A)#1	0.9899
C(30)-H(30B)#1	0.9900
C(10)-Pd(1)-P(1)#1	87.56(4)
C(10)-Pd(1)-P(1)	87.55(4)
P(1)#1-Pd(1)-P(1)	174.81(9)
C(10)-Pd(1)-Br(1)	177.8(3)
P(1)#1-Pd(1)-Br(1)	92.41(4)
P(1)-Pd(1)-Br(1)	92.41(4)
C(30)-Cl(4)-H(30B)#1	32.5(3)
N(1)-S(1)-N(2)	103.8(5)
C(11)-P(1)-C(17)	106.5(3)
C(11)-P(1)-C(23)	102.9(3)
C(17)-P(1)-C(23)	103.4(3)
C(11)-P(1)-Pd(1)	114.4(2)
C(17)-P(1)-Pd(1)	111.4(2)
C(23)-P(1)-Pd(1)	117.0(2)
C(1)-N(1)-S(1)	104.4(7)
C(2)-N(2)-S(1)	106.0(7)
N(1)-C(1)-C(10)	124.1(9)

N(1)-C(1)-C(2)	113.0(9)
C(10)-C(1)-C(2)	122.8(9)
N(2)-C(2)-C(3)	128.1(9)
N(2)-C(2)-C(1)	112.7(9)
C(3)-C(2)-C(1)	119.1(9)
C(2)-C(3)-C(4)	121.6(9)
C(2)-C(3)-Br(2)	117.7(8)
C(4)-C(3)-Br(2)	120.7(7)
C(5)-C(4)-C(3)	122.4(9)
C(5)-C(4)-C(9)	120.0(9)
C(3)-C(4)-C(9)	117.6(9)
C(4)-C(5)-C(6)	118.4(10)
C(4)-C(5)-H(5)	120.8
C(6)-C(5)-H(5)	120.8
C(7)-C(6)-C(5)	123.2(11)
C(7)-C(6)-H(6)	118.4
C(5)-C(6)-H(6)	118.4
C(6)-C(7)-C(8)	120.9(11)
C(6)-C(7)-H(7)	119.6
C(8)-C(7)-H(7)	119.6
C(7)-C(8)-C(9)	119.3(10)
C(7)-C(8)-H(8)	120.3
C(9)-C(8)-H(8)	120.3
C(10)-C(9)-C(8)	118.8(9)
C(10)-C(9)-C(4)	123.0(9)
C(8)-C(9)-C(4)	118.2(9)
C(9)-C(10)-C(1)	115.8(9)

C(9)-C(10)-Pd(1)	124.5(7)
C(1)-C(10)-Pd(1)	119.7(7)
C(16)-C(11)-C(12)	118.7(6)
C(16)-C(11)-P(1)	120.0(5)
C(12)-C(11)-P(1)	121.3(5)
C(13)-C(12)-C(11)	120.8(7)
C(13)-C(12)-H(12)	119.6
C(11)-C(12)-H(12)	119.6
C(12)-C(13)-C(14)	120.0(7)
C(12)-C(13)-H(13)	120.0
C(14)-C(13)-H(13)	120.0
C(15)-C(14)-C(13)	119.7(7)
C(15)-C(14)-H(14)	120.2
C(13)-C(14)-H(14)	120.2
C(14)-C(15)-C(16)	121.1(7)
C(14)-C(15)-H(15)	119.4
C(16)-C(15)-H(15)	119.4
C(15)-C(16)-C(11)	119.7(7)
C(15)-C(16)-H(16)	120.1
C(11)-C(16)-H(16)	120.1
C(22)-C(17)-C(18)	118.4(7)
C(22)-C(17)-P(1)	120.3(6)
C(18)-C(17)-P(1)	121.3(6)
C(19)-C(18)-C(17)	120.0(8)
C(19)-C(18)-H(18)	120.0
C(17)-C(18)-H(18)	120.0
C(20)-C(19)-C(18)	121.0(8)

C(20)-C(19)-H(19)	119.5
C(18)-C(19)-H(19)	119.5
C(19)-C(20)-C(21)	119.6(8)
C(19)-C(20)-H(20)	120.2
C(21)-C(20)-H(20)	120.2
C(20)-C(21)-C(22)	120.2(8)
C(20)-C(21)-H(21)	119.9
C(22)-C(21)-H(21)	119.9
C(17)-C(22)-C(21)	120.8(8)
C(17)-C(22)-H(22)	119.6
C(21)-C(22)-H(22)	119.6
C(28)-C(23)-C(24)	119.0(6)
C(28)-C(23)-P(1)	121.3(6)
C(24)-C(23)-P(1)	119.6(6)
C(23)-C(24)-C(25)	120.9(8)
C(23)-C(24)-H(24)	119.6
C(25)-C(24)-H(24)	119.6
C(26)-C(25)-C(24)	120.0(8)
C(26)-C(25)-H(25)	120.0
C(24)-C(25)-H(25)	120.0
C(25)-C(26)-C(27)	119.4(8)
C(25)-C(26)-H(26)	120.3
C(27)-C(26)-H(26)	120.3
C(26)-C(27)-C(28)	120.5(8)
C(26)-C(27)-H(27)	119.8
C(28)-C(27)-H(27)	119.8
C(23)-C(28)-C(27)	120.3(8)

C(23)-C(28)-H(28)	119.8
C(27)-C(28)-H(28)	119.8
Cl(1)-C(29)-Cl(2)	116.1(12)
Cl(1)-C(29)-H(29A)	108.3
Cl(2)-C(29)-H(29A)	108.3
Cl(1)-C(29)-H(29B)	108.3
Cl(2)-C(29)-H(29B)	108.3
H(29A)-C(29)-H(29B)	107.4
Cl(4)-C(30)-Cl(3)	112.8(9)
Cl(4)-C(30)-H(30A)	109.0
Cl(3)-C(30)-H(30A)	109.0
Cl(4)-C(30)-H(30B)	109.0
Cl(3)-C(30)-H(30B)	109.0
H(30A)-C(30)-H(30B)	107.8
Cl(4)-C(30)-H(30A)#1	132.4(5)
Cl(3)-C(30)-H(30A)#1	109.0(4)
H(30A)-C(30)-H(30A)#1	76.6
H(30B)-C(30)-H(30A)#1	33.5
Cl(4)-C(30)-H(30B)#1	79.0(4)
Cl(3)-C(30)-H(30B)#1	109.03(13)
H(30A)-C(30)-H(30B)#1	33.5
H(30B)-C(30)-H(30B)#1	133.6
H(30A)#1-C(30)-H(30B)#1	107.8

Symmetry transformations used to generate equivalent atoms: #1 x,-y+1,z

Table S24. Anisotropic displacement parameters (Å^2 x 10^3) for li19_34. The anisotropic displacement factor exponent takes the form: $-2 \pi^2 [h^2 a^{*2}U^{11} + ... + 2 h k a^* b^* U^{12}]$

	U11	U22	U33	U23	U13	U12
Pd(1)	18(1)	10(1)	14(1)	0	-1(1)	0
Br(1)	48(1)	35(1)	34(1)	0	-8(1)	0
Br(2)	28(1)	20(1)	21(1)	0	-5(1)	0
Cl(1)	78(3)	57(2)	44(2)	0	7(2)	0
Cl(2)	49(2)	51(2)	46(2)	0	4(1)	0
Cl(3)	53(2)	68(3)	79(3)	0	24(2)	0
Cl(4)	63(3)	127(8)	49(3)	-7(3)	-4(2)	22(3)
S(1)	20(1)	44(2)	25(1)	0	2(1)	0
P(1)	19(1)	10(1)	16(1)	0(1)	1(1)	0(1)
N(1)	27(4)	23(4)	18(4)	0	-6(3)	0
N(2)	20(4)	27(5)	26(5)	0	-7(4)	0
C(1)	25(5)	12(4)	14(4)	0	8(4)	0
C(2)	28(5)	9(4)	19(5)	0	-7(4)	0
C(3)	24(5)	19(5)	12(4)	0	-2(4)	0
C(4)	20(4)	16(4)	14(4)	0	-6(3)	0
C(5)	24(5)	34(6)	25(5)	0	1(4)	0
C(6)	20(5)	42(7)	43(7)	0	8(5)	0
C(7)	24(5)	37(6)	32(5)	0	16(4)	0
C(8)	28(5)	24(5)	25(5)	0	2(4)	0
C(9)	16(4)	19(5)	13(4)	0	7(3)	0
C(10)	20(5)	7(4)	27(5)	0	8(4)	0
C(11)	23(3)	19(3)	16(3)	2(3)	5(2)	2(3)
C(12)	24(3)	17(3)	32(4)	1(3)	4(3)	-2(3)
C(13)	37(4)	22(4)	40(4)	3(3)	9(3)	5(3)
C(14)	27(4)	33(4)	34(4)	7(3)	12(3)	9(3)
C(15)	23(3)	34(4)	29(4)	2(3)	5(3)	-1(3)

C(16)	22(3)	21(3)	24(3)	0(3)	3(3)	-1(3)
C(17)	19(3)	16(3)	23(3)	-1(3)	-3(2)	0(2)
C(18)	40(4)	23(4)	44(5)	12(4)	18(4)	5(3)
C(19)	48(5)	28(4)	55(5)	21(4)	20(4)	7(4)
C(20)	40(4)	40(5)	32(4)	13(4)	13(3)	-4(4)
C(21)	60(6)	30(4)	50(5)	3(4)	31(5)	4(4)
C(22)	48(5)	23(4)	39(4)	1(4)	24(4)	3(4)
C(23)	27(3)	13(3)	19(3)	0(2)	0(3)	1(2)
C(24)	34(4)	22(4)	29(4)	-5(3)	0(3)	-4(3)
C(25)	63(6)	25(4)	26(4)	-7(3)	0(4)	-7(4)
C(26)	56(5)	27(4)	30(4)	-6(4)	-15(4)	-8(4)
C(27)	36(4)	39(5)	39(5)	-2(4)	-10(3)	-9(4)
C(28)	29(4)	25(4)	28(4)	-4(3)	-3(3)	-1(3)
C(29)	67(11)	90(13)	44(9)	0	-6(8)	0
C(30)	50(8)	57(9)	47(8)	0	9(7)	0

Table S25. Hydrogen coordinates (x 10^4) and isotropic displacement parameters (Å^2 x 10^3) for li19_34.

	x	y	z	U(eq)
H(5)	3187	5000	536	34
H(6)	5595	5000	678	42
H(7)	6906	5000	1992	36
H(8)	5883	5000	3286	31
H(12)	5182	1747	4132	29
H(13)	7330	1076	4141	39
H(14)	9347	1849	4644	37

H(15)	9208	3312	5042	34
H(16)	7067	4006	5032	27
H(18)	4229	1781	5470	42
H(19)	3271	1234	6668	51
H(20)	1956	2114	7446	44
H(21)	1601	3570	7051	53
H(22)	2555	4137	5858	42
H(24)	4599	3111	2834	34
H(25)	3278	2510	1627	46
H(26)	1065	1982	1750	47
H(27)	125	2171	3054	47
H(28)	1463	2747	4268	33
H(29A)	8380	5523	7031	82
H(29B)	8380	4477	7031	82
H(30A)	6482	4598	7934	61
H(30B)	6599	5596	8250	61

5. Computational details and spectrums

Two stacked structures were investigated theoretically, trimer **7** and the corresponding pentamer. Two distinct classes of computational methods were used. The first one comprised the *ab initio* method second-order Møller-Plesset perturbation theory [6] including the empirical scaled opposite-spin (SOS) [7,8] (SOS-MP2) for the ground state geometry optimizations and the corresponding second-order algebraic diagrammatic construction (ADC(2)) approach [9,10] for the excited state calculations including optimizations in the S_1 state. The split valence SV(P) basis [11] was used.

Optimization of ground-state geometries was performed with the SOS-MP2 method, and excited states were calculated with the ADC(2) method using the SV(P) basis set [12]. Solvation effects for tetrahydrofuran were treated for vertical excitations using the conductor-like screening model (COSMO) [13,14] based on a continuum approach. The values of $\varepsilon = 7.52$ [15] for the relative dielectric constant and $n = 1.4073$ [16] for the refractive index of tetrahydrofuran were used. For S_1 geometry optimization ADC(2)/SV(P) method was also used. Adiabatic excitation energies were calculated by using the minimum ground state (S_0) and minimum excited state (S_1).

The second class of methods is based on density functional theory (DFT) using the long-range corrected ωB97XD functional [17] and the SVP basis set [11]. Excited state calculations, including geometry optimizations of the S_1 state, were performed using the time-dependent density functional theory [18]. Environmental effects were included by means of the polarizable continuum model (PCM) (15) using for tetrahydrofuran (THF) as solvent with values of $\varepsilon = 7.52$ [15] for the relative dielectric constant and $n = 1.41$ [16] for the refractive index. Vertical PCM excitations used the state-specific corrected linear response (cLR) method [19]. Adiabatic excitation energies were calculated by using the minimum ground state (S_0) and minimum excited state (S_1) energies.

Charge transfer between fragments for a given electronic transition was described using the q(CT) value [20] computed from the omega matrix Ω_{AB}^{α} , via the transition density $\mathbf{D}_{0\alpha}$ as follows,

$$\Omega_{AB}^{\alpha} = \frac{1}{2}\sum_{\substack{a\in A\\ b\in B}}\left[\left(\mathbf{D}^{0\alpha}\right)\right] \Omega_{AB}^{\alpha} = \frac{1}{2}\sum_{\substack{a\in A\\ b\in B}}\left[\left(\mathbf{D}^{0\alpha,[AO]}\mathbf{S}^{[AO]}\right)_{ab}\left(\mathbf{S}^{[AO]}\mathbf{D}^{0\alpha,[AO]}\right)_{ab}\right] \quad \text{(S1)}$$

α labels the excited state and AO denotes the atomic orbitals. The matrix Ω_{AB}^{α} describes the charge transfer contribution from fragment A to fragment B ($A \neq B$), and contributions from excitations occurring on the same fragment ($A = B$). The total CT character for a given system composed of multiple fragments is then given by q(CT):

$$q(CT)=\frac{1}{\Omega^{\alpha}}\sum_{A}\sum_{A\neq B}\Omega_{AB}^{\alpha} \qquad \text{(S1)}$$

where is the sum of the charge transfer values for all fragment pairs, A and B. When q(CT) = 1 e, one electron has been transferred, and when q(CT) = 0 e, the transition is a local excitation or Frenkel excitonic state.

The SOS-MP2 and ADC(2) calculations were performed with the Turbomole program system [21]; all DFT calculations were performed using Gaussian09 [22]. Charge transfer (qCT) [20] due to electronic excitation is computed from an analysis of the transition density matrix and Natural Transition Orbitals (NTOs) [23] by means of the TheoDore program [20, 24, 25]. The CT is defined from the stacked benzene system to the entirety of naphthalene rings. A transition is characterized as having CT character if qCT ≥ 0.5 *e*, otherwise is classified as local excitation (LE).

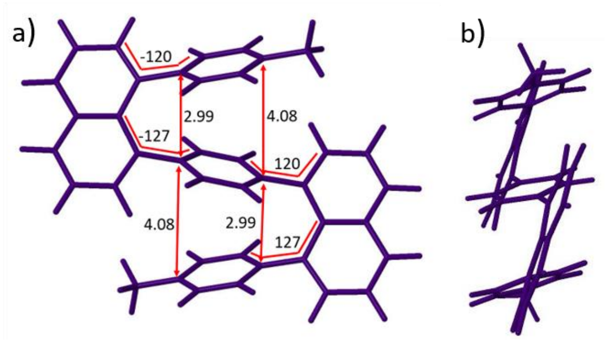

Figure S30. Selected geometry data for the trimer structure optimized for the ground state using the ωB97XD/SVP method in THF: a) front view and b) side view. Distances are given in Å and angles in degrees.

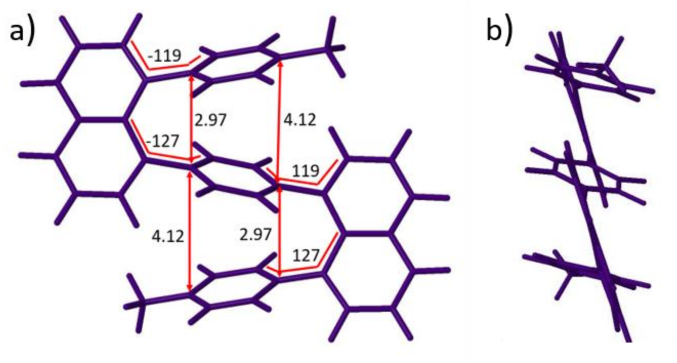

Figure S31. Selected geometry data for the trimer structure optimized for the ground state using the SOS-MP2/SV(P) method in the gas phase: a) front view and b) side view. Distances are given in Å and angles in degrees.

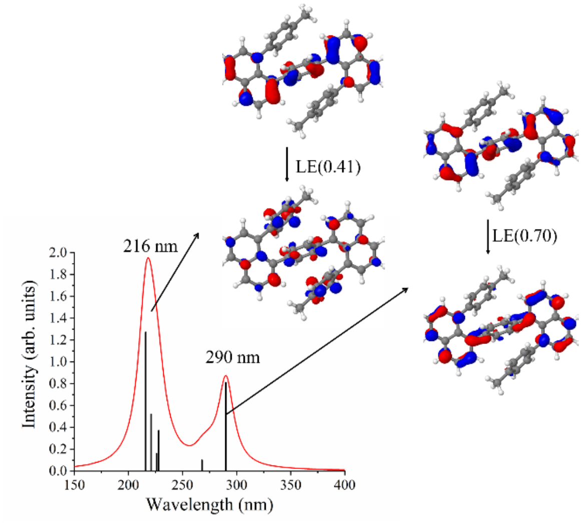

Figure S32. UV spectrum and characterization of most important transitions for the trimer by means of NTOs (occupation fractions in parentheses) using the ωB97XD/SVP method in THF.

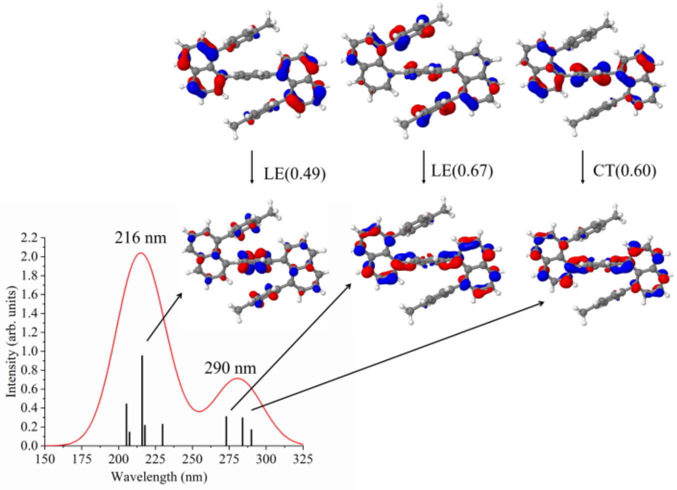

Figure S33. UV spectrum and characterization of most important transitions for the trimer by means of NTOs (occupation fractions in parentheses) using the ADC(2)/SV(P) method in the gas phase.

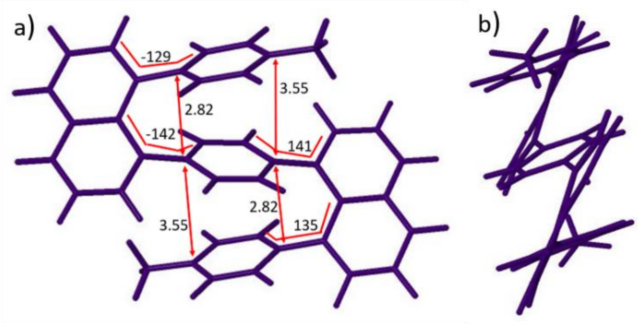

Figure S34. Selected geometry data for the trimer structure optimized for the S_1 state using the ADC(2)/SV(P) method in the gas phase: a) front view and b) side view. Distances are given in Å and angles in degrees.

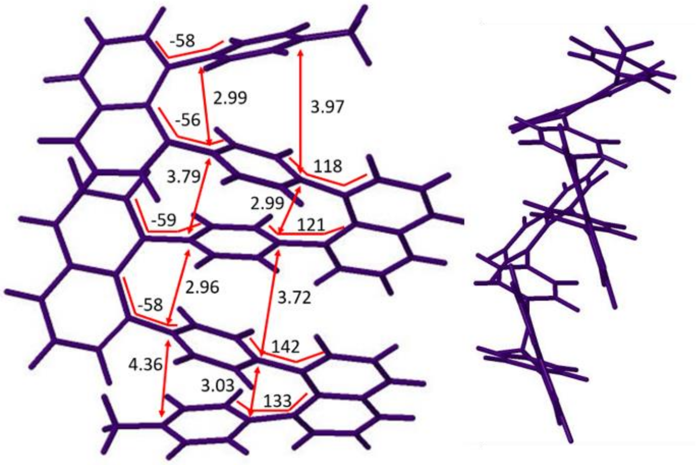

Figure S35. Selected geometry data for the pentamer structure optimized for the S_1 state using the ωB97XD/SVP method in the gas phase: a) front view and b) side view. Distances are given in Å and angles in degrees.

Optimized Cartesian coordinates for different structures.

a) SOS-MP2/SV(P) method for the gas phase for Trimer

C	-0.6376992	-0.4307363	1.1644902
H	-1.1226930	-0.7833988	2.0849809
C	-0.7734436	-1.1671986	-0.0166739
H	-1.3680308	-2.0902835	-0.0216928
C	-0.1483022	-0.7377105	-1.2019480
C	-0.2367780	-1.5449024	-2.4521463
C	0.9733277	-2.0019159	-2.9701370
H	1.9003880	-1.6993604	-2.4664419

C	1.0331624	-2.8866797	-4.0771074
H	2.0064345	-3.2312777	-4.4508478
C	-0.1398908	-3.3446653	-4.6408413
H	-0.1188558	-4.0655329	-5.4694235
C	-1.4065201	-2.8895989	-4.1608257
C	-1.4799842	-1.9309540	-3.0875800
C	-2.7899659	-1.4234826	-2.7289772
C	-3.9216359	-1.9605714	-3.3391299
H	-4.9049311	-1.5559826	-3.0658325
C	-3.8386586	-2.9616423	-4.3408059
H	-4.7558297	-3.3518566	-4.8014377
C	-2.5993354	-3.3965127	-4.7620410
H	-2.5102488	-4.1362055	-5.5690725
C	-3.0273394	-0.2824481	-1.7946685
C	-2.4604461	0.9849340	-2.0348646
H	-1.7983707	1.1261751	-2.8989770
C	-2.7325772	2.0607414	-1.1837042
H	-2.2683014	3.0365577	-1.3850667
C	-3.5905644	1.9172827	-0.0745866
C	-4.1740998	0.6578825	0.1472273
H	-4.8521270	0.5162032	1.0008157
C	-3.8984820	-0.4261834	-0.6995556
H	-4.3473910	-1.4072617	-0.4924078
C	-3.9099209	3.1046393	0.8105572
H	-2.9999770	3.6950321	1.0270318
H	-4.3455792	2.7788638	1.7733085
H	-4.6412597	3.7781698	0.3211664
C	0.6376868	0.4308282	-1.1644734
H	1.1227311	0.7835576	-2.0848935
C	0.7734205	1.1672946	0.0167132
H	1.3679780	2.0903838	0.0218219
C	0.1482746	0.7377797	1.2019609
C	0.2367730	1.5449214	2.4521282
C	-0.9733107	2.0019296	2.9701354
H	-1.9003634	1.6993579	2.4664232
C	-1.0331260	2.8866733	4.0771202
H	-2.0063566	3.2313166	4.4509106
C	0.1399870	3.3445972	4.6408188
H	0.1191168	4.0654165	5.4694336
C	1.4065475	2.8895107	4.1607537
C	1.4800157	1.9309158	3.0875045

C	2.7899453	1.4234448	2.7289126
C	3.9216817	1.9605136	3.3390763
H	4.9049309	1.5558770	3.0657761
C	3.8386455	2.9615610	4.3407769
H	4.7558388	3.3517666	4.8014623
C	2.5993887	3.3963756	4.7619832
H	2.5101280	4.1360085	5.5690884
C	3.0272935	0.2824444	1.7946391
C	2.4604138	-0.9849408	2.0348698
H	1.7983525	-1.1261670	2.8989843
C	2.7325807	-2.0607294	1.1837084
H	2.2683642	-3.0365691	1.3851059
C	3.5905529	-1.9172417	0.0745792
C	4.1740536	-0.6578057	-0.1472644
H	4.8520873	-0.5160759	-1.0008361
C	3.8983815	0.4262244	0.6995223
H	4.3471921	1.4073636	0.4924206
C	3.9099201	-3.1046143	-0.8105375
H	2.9999853	-3.6950641	-1.0269163
H	4.3455026	-2.7788757	-1.7733407
H	4.6413134	-3.7780987	-0.3211526

b) wB97XD/SVP method for the gas phase for Trimer

C	-0.37846500	-0.60344100	1.18859400
H	-0.67818600	-1.06359900	2.13303200
C	0.90743100	-0.09977800	1.04632200
H	1.61759200	-0.17853300	1.87213900
C	1.30974500	0.49970100	-0.14985200
C	2.67427600	1.07522200	-0.30070800
C	2.72839900	2.42467200	-0.59692400
H	1.78617900	2.95357400	-0.75629400
C	3.94253700	3.13888400	-0.63763700
H	3.93812500	4.20716700	-0.86405400
C	5.11131300	2.48893900	-0.34526900
H	6.06147300	3.02817900	-0.32757200
C	5.11845400	1.09631700	-0.06115300
C	3.89822200	0.34097800	-0.08583900
C	3.99753800	-1.08846500	0.10214600
C	5.22401800	-1.64697800	0.41218800
H	5.28275500	-2.72979000	0.54317800

C	6.40332800	-0.88014900	0.50605000
H	7.35034700	-1.36741300	0.74651000
C	6.35253200	0.46246500	0.24628200
H	7.26039800	1.07021300	0.26891100
C	2.87333600	-2.05131100	-0.09066200
C	2.18159700	-2.12749400	-1.30522500
H	2.44607800	-1.44422000	-2.11517100
C	1.15135500	-3.04220800	-1.48276100
H	0.61633000	-3.06661000	-2.43585200
C	0.78470500	-3.93132900	-0.46441400
C	1.49342700	-3.87096400	0.73840200
H	1.23115700	-4.55203800	1.55272900
C	2.52288400	-2.94950500	0.92143700
H	3.04517500	-2.90576400	1.88049400
C	-0.31562100	-4.93698700	-0.68417700
H	-1.18440600	-4.47672200	-1.17855600
H	-0.65660400	-5.37778000	0.26388800
H	0.02898400	-5.76212500	-1.32824900
C	0.37851800	0.60384700	-1.18831000
H	0.67825100	1.06396300	-2.13276500
C	-0.90739000	0.10022800	-1.04602300
H	-1.61755500	0.17897900	-1.87183800
C	-1.30966300	-0.49936500	0.15010500
C	-2.67411500	-1.07512100	0.30079600
C	-2.72808500	-2.42459900	0.59690900
H	-1.78581200	-2.95336500	0.75639800
C	-3.94213500	-3.13898100	0.63737500
H	-3.93761300	-4.20727400	0.86374000
C	-5.11095500	-2.48918900	0.34482200
H	-6.06103700	-3.02856200	0.32692200
C	-5.11823400	-1.09656300	0.06072200
C	-3.89810900	-0.34107400	0.08567200
C	-3.99751400	1.08832700	-0.10244600
C	-5.22398100	1.64671200	-0.41276100
H	-5.28279000	2.72951000	-0.54383100
C	-6.40319600	0.87975200	-0.50681500
H	-7.35021900	1.36690400	-0.74748800
C	-6.35231600	-0.46285000	-0.24698700
H	-7.26011300	-1.07069600	-0.26979600
C	-2.87339600	2.05122400	0.09059500
C	-2.18211100	2.12756900	1.30541500

H	-2.44685600	1.44434900	2.11532200
C	-1.15208400	3.04244300	1.48328800
H	-0.61746200	3.06703500	2.43660200
C	-0.78513300	3.93151500	0.46499500
C	-1.49333200	3.87092000	-0.73810700
H	-1.23083300	4.55196200	-1.55238600
C	-2.52262300	2.94932100	-0.92146400
H	-3.04455700	2.90545500	-1.88070900
C	0.31502500	4.93727000	0.68515600
H	1.18436000	4.47665800	1.17825700
H	0.65507700	5.37939500	-0.26261500
H	-0.02932900	5.76141100	1.33062800

c) wB97XD/SVP method in THF for Trimer

C	-0.37757300	-0.60643100	1.18771500
H	-0.67525300	-1.06815400	2.13209400
C	0.90931600	-0.10476400	1.04423600
H	1.61936900	-0.18772500	1.86989200
C	1.31037900	0.49809700	-0.15100500
C	2.67348100	1.07859000	-0.30080100
C	2.72236900	2.42977400	-0.59239000
H	1.77919200	2.95755200	-0.74975700
C	3.93355200	3.15018400	-0.63222500
H	3.92331000	4.21905800	-0.85528700
C	5.10603200	2.50445600	-0.34281200
H	6.05382600	3.04771700	-0.32398800
C	5.11860100	1.11047600	-0.06172300
C	3.90124100	0.34918700	-0.08766300
C	4.00731300	-1.08030000	0.09950900
C	5.23676600	-1.63282600	0.41108600
H	5.30226200	-2.71481300	0.54607600
C	6.41323300	-0.86052400	0.50527500
H	7.36191200	-1.34388300	0.74697800
C	6.35660500	0.48249000	0.24538000
H	7.26148900	1.09460400	0.26856000
C	2.88717800	-2.04846100	-0.09275200
C	2.19360700	-2.12797000	-1.30628100
H	2.45102700	-1.44304900	-2.11739800
C	1.16759000	-3.04917900	-1.48240100
H	0.63169100	-3.07713200	-2.43503400

C	0.80632700	-3.93941000	-0.46250000
C	1.51738200	-3.87496900	0.73985100
H	1.25860500	-4.55565300	1.55559200
C	2.54319700	-2.94878300	0.92061300
H	3.06636000	-2.90331200	1.87912400
C	-0.29012700	-4.95056000	-0.67643500
H	-1.14086400	-4.50896900	-1.21645600
H	-0.66082700	-5.35318600	0.27730400
H	0.07158900	-5.80109100	-1.27667100
C	0.37746000	0.60647200	-1.18781600
H	0.67513700	1.06820500	-2.13219100
C	-0.90943000	0.10481100	-1.04433600
H	-1.61948200	0.18777300	-1.86999300
C	-1.31048700	-0.49807900	0.15089400
C	-2.67359300	-1.07856200	0.30068400
C	-2.72253200	-2.42976200	0.59220400
H	-1.77937900	-2.95758600	0.74955300
C	-3.93374300	-3.15013200	0.63196900
H	-3.92354200	-4.21902000	0.85496700
C	-5.10619600	-2.50434900	0.34255900
H	-6.05400800	-3.04757800	0.32367700
C	-5.11870900	-1.11035400	0.06154300
C	-3.90132200	-0.34911700	0.08755600
C	-4.00731500	1.08037800	-0.09953900
C	-5.23673400	1.63298400	-0.41111200
H	-5.30217900	2.71498200	-0.54602700
C	-6.41323500	0.86073900	-0.50537600
H	-7.36188500	1.34415800	-0.74707000
C	-6.35667600	-0.48229500	-0.24555800
H	-7.26158800	-1.09436600	-0.26879800
C	-2.88712000	2.04844700	0.09284300
C	-2.19362100	2.12783400	1.30641800
H	-2.45116400	1.44290500	2.11749000
C	-1.16753400	3.04894600	1.48265600
H	-0.63170400	3.07681100	2.43533000
C	-0.80611300	3.93918000	0.46281600
C	-1.51707200	3.87483800	-0.73960100
H	-1.25815500	4.55551500	-1.55530400
C	-2.54296400	2.94876200	-0.92047500
H	-3.06604700	2.90336300	-1.87903400
C	0.29037800	4.95026700	0.67686200

H	1.14071600	4.50885200	1.21765000
H	0.66171200	5.35232400	-0.27687200
H	-0.07158100	5.80119700	1.27639000

d) SOS-MP2/SV(P) method for the gas phase for the Pentamer

C	0.0333877	1.8772037	-2.3000322
C	-1.1125257	2.0072023	-3.0830551
C	-1.1068681	2.6779560	-4.3313338
C	0.0757688	3.2025934	-4.8086519
C	1.2787975	3.1058445	-4.0442618
C	1.2777208	2.4729392	-2.7477460
C	2.4825161	3.6534945	-4.5851953
C	3.6645222	3.5979484	-3.8764733
C	3.6686578	3.0232807	-2.5797845
C	2.5202385	2.4880763	-1.9986627
C	-1.6479076	-4.4545665	4.2220601
C	-1.9843876	-5.2403894	3.1395914
C	-1.9939866	-4.6661029	1.8426865
C	-1.6516605	-3.3341828	1.6154787
C	-1.2289366	-2.5026784	2.7268428
C	-1.2735435	-3.0870753	4.0452001
C	-0.7686397	-1.1312164	2.6230565
C	-0.4728663	-0.4153143	3.7820561
C	-0.5727202	-0.9879194	5.0743639
C	-0.9507499	-2.3083373	5.1985221
C	-0.1325568	1.0942831	-1.0396948
C	0.5682619	-0.1063910	-0.8058715
C	0.3803789	-0.8249535	0.3783261
C	-0.5571404	-0.3970037	1.3405114
C	-1.2627006	0.7967190	1.1029109
C	-1.0629607	1.5249112	-0.0761286
C	2.6701990	1.9730183	-0.6058442
C	3.5924143	0.9446744	-0.3382023
C	3.7417527	0.4360562	0.9582006
C	2.9795583	0.9473085	2.0249721
C	2.0693016	1.9905040	1.7579269
C	1.9401302	2.5170802	0.4700131
C	-1.9404767	-1.9808416	-2.4904625
C	-2.7029564	-1.3627203	-1.4782105
C	-2.6418991	-1.8152879	-0.1574754

C	-1.7674423	-2.8593397	0.2051962
C	-1.0197870	-3.4896350	-0.8064707
C	-1.1017426	-3.0533408	-2.1348889
C	2.9360200	-1.0847310	5.8361557
C	2.7958451	-1.7682199	4.6466088
C	2.8471332	-1.0508126	3.4261375
C	3.0782850	0.3237448	3.3763470
C	3.3206821	1.0504712	4.6070810
C	3.1876203	0.3212294	5.8442981
C	3.6951232	2.4492223	4.6931794
C	3.7934578	3.0574431	5.9430359
C	3.5843213	2.3440860	7.1508746
C	3.3117168	0.9930771	7.0991350
C	-4.4593357	-1.7086492	-4.3519325
C	-5.5337605	-1.3544273	-5.1655100
C	-5.3657829	-0.6055871	-6.3582088
C	-4.0955882	-0.2439688	-6.7557766
C	-2.9592803	-0.5701822	-5.9534418
C	-3.1234152	-1.2626496	-4.6990053
C	-1.6587685	-0.2046389	-6.4174021
C	-0.5375367	-0.4883537	-5.6662544
C	-0.6911586	-1.0979076	-4.3966719
C	-1.9375676	-1.4652738	-3.8900762
C	-4.7746171	-2.5873145	-3.1867533
C	-5.7290394	-2.1824353	-2.2350228
C	-6.0396495	-2.9966874	-1.1373947
C	-5.4214593	-4.2478170	-0.9658334
C	-4.4965445	-4.6663660	-1.9429378
C	-4.1852623	-3.8580530	-3.0423825
C	4.6972026	4.9583602	1.2964063
C	3.6804825	5.3222953	2.1966601
C	3.3641803	4.5109635	3.2948189
C	4.0440509	3.2988352	3.5161278
C	5.0922851	2.9520462	2.6419998
C	5.4024909	3.7640249	1.5451035
C	4.9949431	5.7971664	0.0712457
C	-5.7033935	-5.0991249	0.2544657
H	-2.0393210	1.5364660	-2.7315439
H	-2.0316337	2.7456921	-4.9186715
H	0.1127452	3.7018235	-5.7865192
H	2.4475654	4.1215044	-5.5782603

H	4.5897575	4.0162471	-4.2943280
H	4.5962636	3.0224406	-1.9925620
H	-1.6635791	-4.8681350	5.2395234
H	-2.2690387	-6.2920405	3.2761894
H	-2.3103734	-5.2743451	0.9851925
H	-0.1127042	0.6160646	3.6791010
H	-0.3185175	-0.3885751	5.9580360
H	-1.0073469	-2.7841174	6.1870912
H	1.2924224	-0.4648590	-1.5485567
H	0.9473572	-1.7491060	0.5477302
H	-1.9979953	1.1432829	1.8421850
H	-1.6167518	2.4590782	-0.2430347
H	4.1762274	0.5158262	-1.1644202
H	4.4687099	-0.3649481	1.1500222
H	1.4620291	2.4018193	2.5739773
H	1.2389369	3.3410226	0.2870125
H	-3.3608362	-0.5221949	-1.7323999
H	-3.2569164	-1.3319803	0.6121112
H	-0.3400703	-4.3113549	-0.5417902
H	-0.5130207	-3.5585410	-2.9127399
H	2.8521679	-1.6104385	6.7969876
H	2.5968902	-2.8477649	4.6388386
H	2.6475617	-1.5799985	2.4860401
H	4.0868637	4.1143605	5.9877159
H	3.6780455	2.8608705	8.1149571
H	3.1926362	0.4107679	8.0228891
H	-6.5357258	-1.7091538	-4.8909915
H	-6.2392384	-0.3534335	-6.9736753
H	-3.9371686	0.2949003	-7.6997539
H	-1.5696594	0.3108789	-7.3832713
H	0.4620830	-0.2029137	-6.0188714
H	0.1922515	-1.2417056	-3.7621282
H	-6.2066530	-1.1982412	-2.3360875
H	-6.7750339	-2.6490443	-0.3981114
H	-4.0165381	-5.6507154	-1.8468514
H	-3.4656638	-4.2099387	-3.7929658
H	3.1217084	6.2550653	2.0355519
H	2.5495289	4.8029823	3.9715790
H	5.6606163	2.0293388	2.8169651
H	6.2190041	3.4676123	0.8712351
H	4.6923127	6.8493789	0.2258742

H	6.0744519	5.7826237	-0.1703278
H	4.4448728	5.4111960	-0.8098594
H	-6.7044021	-4.8804556	0.6703160
H	-5.6622748	-6.1767382	0.0076318
H	-4.9553777	-4.9040671	1.0484218

e) wB97XD/SVP method for the gas phase Pentamer

C	0.79955200	2.79104900	-0.63512300
C	2.01659700	3.03354200	-1.24446500
C	2.58769200	4.31862600	-1.31373700
C	1.92812100	5.37296000	-0.74394700
C	0.66669900	5.18925300	-0.11567700
C	0.05183200	3.89156800	-0.07075200
C	0.02273700	6.31547800	0.46543700
C	-1.19742800	6.19135200	1.07216900
C	-1.83120700	4.93247200	1.09213500
C	-1.26035600	3.80506700	0.53129700
C	-0.02221900	-6.31582600	0.46532500
C	1.19799700	-6.19162500	1.07193400
C	1.83166200	-4.93268400	1.09191500
C	1.26064600	-3.80530200	0.53120900
C	-0.05159600	-3.89188800	-0.07072400
C	-0.66635100	-5.18962700	-0.11565600
C	-0.79947000	-2.79140300	-0.63495700
C	-2.01658000	-3.03396800	-1.24414700
C	-2.58756700	-4.31909500	-1.31342300
C	-1.92782600	-5.37341000	-0.74378800
C	0.37573200	1.36108300	-0.60493000
C	0.19693000	0.66488000	0.59491300
C	-0.19690500	-0.66511700	0.59497300
C	-0.37574700	-1.36140600	-0.60481100
C	-0.18882200	-0.66964100	-1.80421700
C	0.18872200	0.66925000	-1.80427900
C	-2.08253800	2.56214000	0.59424300
C	-2.44234700	2.02718200	1.83406200
C	-3.17170400	0.84545500	1.91555400
C	-3.57075700	0.16776900	0.76032400
C	-3.22910600	0.71850100	-0.47985200
C	-2.52066700	1.90827400	-0.56191200
C	3.57061400	-0.16774800	0.76037900

C	3.22902800	-0.71846500	-0.47981900
C	2.52075500	-1.90833300	-0.56193100
C	2.08269600	-2.56229100	0.59419500
C	2.44244200	-2.02734800	1.83403900
C	3.17164500	-0.84553200	1.91558200
C	-5.19154600	-3.80711500	1.00288700
C	-4.06379900	-3.44329000	1.68596800
C	-3.60336100	-2.11609800	1.59248200
C	-4.26783000	-1.14545800	0.86471500
C	-5.51240900	-1.47684600	0.21038400
C	-5.92757500	-2.85101300	0.25255400
C	-6.37859000	-0.54601400	-0.47769800
C	-7.48629000	-1.02051700	-1.15565400
C	-7.83860100	-2.38537400	-1.16753200
C	-7.08491700	-3.27703300	-0.45369800
C	6.37835500	0.54636500	-0.47768000
C	7.48596700	1.02101600	-1.15568200
C	7.83810300	2.38591700	-1.16756700
C	7.08433800	3.27747500	-0.45368900
C	5.92708400	2.85129800	0.25261000
C	5.51208000	1.47708200	0.21043500
C	5.19097600	3.80729700	1.00300100
C	4.06330700	3.44332800	1.68613200
C	3.60301300	2.11608600	1.59263600
C	4.26755600	1.14554500	0.86481300
C	6.18938100	-0.93288300	-0.47462200
C	6.08116900	-1.63396800	-1.68062100
C	5.85252300	-3.00638800	-1.69262100
C	5.73148400	-3.72786500	-0.50043700
C	5.90126400	-3.03333400	0.70213400
C	6.13345600	-1.66196200	0.71673800
C	-5.73123800	3.72814600	-0.50046500
C	-5.85234600	3.00667700	-1.69264300
C	-6.08111700	1.63427300	-1.68063600
C	-6.18940400	0.93320500	-0.47463600
C	-6.13337200	1.66228300	0.71672400
C	-5.90105400	3.03362900	0.70211300
C	-5.38737300	5.19400000	-0.51411000
C	5.38773600	-5.19374600	-0.51410400
H	2.57042100	2.18380400	-1.64860500
H	3.56101300	4.45403700	-1.78889100

H	2.36143900	6.37594100	-0.75884800
H	0.52433500	7.28488600	0.41439500
H	-1.68939100	7.05669900	1.52090400
H	-2.82265600	4.83798200	1.54130300
H	-0.52373800	-7.28527600	0.41428200
H	1.69008000	-7.05695100	1.52057600
H	2.82314600	-4.83812700	1.54099300
H	-2.57053000	-2.18425300	-1.64816000
H	-3.56093000	-4.45457000	-1.78847300
H	-2.36105400	-6.37643000	-0.75871700
H	0.34219100	1.18200900	1.54474900
H	-0.34209300	-1.18218300	1.54485600
H	-0.32251500	-1.19617000	-2.75223800
H	0.32238500	1.19570500	-2.75234500
H	-2.11553800	2.52811500	2.74865800
H	-3.44081100	0.43994600	2.89381900
H	-3.52679000	0.20675500	-1.39685900
H	-2.26676500	2.31939200	-1.54049500
H	3.52665400	-0.20664500	-1.39680400
H	2.26689800	-2.31944100	-1.54053000
H	2.11569700	-2.52837400	2.74860800
H	3.44072100	-0.44003500	2.89386100
H	-5.54405500	-4.84130900	1.01748400
H	-3.49168200	-4.17702200	2.25664800
H	-2.65560600	-1.85074100	2.06432400
H	-8.12947700	-0.29962200	-1.66538200
H	-8.72595100	-2.71367200	-1.71254400
H	-7.36150100	-4.33331300	-0.40937400
H	8.12923000	0.30020900	-1.66543900
H	8.72538800	2.71432700	-1.71261500
H	7.36078900	4.33379000	-0.40936500
H	5.54335400	4.84153600	1.01758500
H	3.49112800	4.17699100	2.25683700
H	2.65530300	1.85061100	2.06450300
H	6.13372800	-1.08586800	-2.62487400
H	5.74266900	-3.52410600	-2.64944600
H	5.83542100	-3.57368400	1.65031200
H	6.24177400	-1.14108100	1.67055300
H	-5.74246800	3.52438600	-2.64947000
H	-6.13374900	1.08617900	-2.62488800
H	-6.24174100	1.14141000	1.67053800

H	-5.83515300	3.57397800	1.65028800
H	-5.86213100	5.71279000	-1.35979800
H	-5.70242100	5.69184600	0.41424200
H	-4.29782400	5.33212900	-0.61158900
H	5.86311600	-5.71262900	-1.35938900
H	5.70218000	-5.69143400	0.41453700
H	4.29826700	-5.33197200	-0.61231200

f) wB97XD/SVP method in THF for the Pentamer

C	-0.79801300	-2.79152000	-0.62273300
C	-2.02192500	-3.03131300	-1.22004000
C	-2.59156000	-4.31734500	-1.29399800
C	-1.92223700	-5.37675200	-0.74393300
C	-0.65323200	-5.19540000	-0.12901400
C	-0.04167300	-3.89575000	-0.07669700
C	0.00099000	-6.32721300	0.43126300
C	1.22813500	-6.20552300	1.02558900
C	1.85728200	-4.94375800	1.05462700
C	1.27626300	-3.81121800	0.51392800
C	-0.00067200	6.32681800	0.43240800
C	-1.22778400	6.20507000	1.02678900
C	-1.85699100	4.94333000	1.05561900
C	-1.27605200	3.81086700	0.51467600
C	0.04185400	3.89545000	-0.07600600
C	0.65346400	5.19508400	-0.12813200
C	0.79809800	2.79128800	-0.62230000
C	2.02196700	3.03113900	-1.21967800
C	2.59164200	4.31716000	-1.29346100
C	1.92241800	5.37649500	-0.74313300
C	-0.37398200	-1.36126100	-0.59061700
C	-0.19564500	-0.66584100	0.60980200
C	0.19575100	0.66540800	0.60990700
C	0.37401100	1.36104100	-0.59040200
C	0.18827300	0.66957600	-1.79028100
C	-0.18834400	-0.66957800	-1.79038700
C	2.09334000	-2.56501200	0.58698200
C	2.45589300	-2.04283000	1.83171300
C	3.18374900	-0.86079300	1.92344600
C	3.57877600	-0.17064000	0.77393300
C	3.23535300	-0.70888700	-0.47124100

C	2.52704900	-1.89832700	-0.56359900
C	-3.57883700	0.17041400	0.77393800
C	-3.23533800	0.70886800	-0.47112600
C	-2.52693400	1.89826600	-0.56323600
C	-2.09319600	2.56469200	0.58748500
C	-2.45578500	2.04228000	1.83210900
C	-3.18374500	0.86028700	1.92359700
C	5.17663000	3.81280800	1.05066900
C	4.06083200	3.43084300	1.74441600
C	3.60777500	2.10155000	1.63870300
C	4.27006800	1.14460300	0.89040200
C	5.50521200	1.49321700	0.22675500
C	5.90968000	2.87077700	0.27896900
C	6.37130000	0.57689200	-0.48165500
C	7.46473300	1.06971500	-1.17095700
C	7.80445000	2.43850700	-1.17395800
C	7.05348200	3.31591700	-0.43875300
C	-6.37152600	-0.57639900	-0.48169400
C	-7.46510600	-1.06888400	-1.17100600
C	-7.80506100	-2.43761500	-1.17426900
C	-7.05418500	-3.31531400	-0.43931600
C	-5.91024900	-2.87052400	0.27841100
C	-5.50554700	-1.49302300	0.22646200
C	-5.17730100	-3.81284900	1.04985400
C	-4.06139000	-3.43122400	1.74360500
C	-3.60812400	-2.10197800	1.63816700
C	-4.27030800	-1.14475900	0.89012400
C	-6.19954700	0.90539700	-0.48686500
C	-6.09840400	1.60084100	-1.69736000
C	-5.89381100	2.97746000	-1.71746800
C	-5.78949400	3.70917300	-0.52904600
C	-5.94937700	3.01888400	0.67800200
C	-6.15783600	1.64308900	0.70041500
C	5.78994400	-3.70876300	-0.52975000
C	5.89386300	-2.97679100	-1.71804500
C	6.09825900	-1.60014300	-1.69770300
C	6.19958000	-0.90493300	-0.48709200
C	6.15826200	-1.64287800	0.70005000
C	5.95001200	-3.01869700	0.67740400
C	5.47335300	-5.18079400	-0.55166700
C	-5.47269900	5.18116300	-0.55076500

H	-2.58045200	-2.17964300	-1.61358500
H	-3.56928600	-4.45170900	-1.76049700
H	-2.35302000	-6.38066300	-0.76427900
H	-0.49815400	-7.29758300	0.37502000
H	1.72846700	-7.07390000	1.45886000
H	2.85194400	-4.85281100	1.49745700
H	0.49850900	7.29717800	0.37631300
H	-1.72805100	7.07338900	1.46025500
H	-2.85164000	4.85234700	1.49847100
H	2.58042100	2.17951600	-1.61342700
H	3.56933000	4.45157000	-1.76002800
H	2.35324400	6.38039100	-0.76333000
H	-0.34031400	-1.18340600	1.55969600
H	0.34050200	1.18280000	1.55988100
H	0.32186900	1.19489900	-2.73900500
H	-0.32199800	-1.19473700	-2.73919400
H	2.13376200	-2.55312800	2.74276500
H	3.45315600	-0.46601400	2.90602500
H	3.52933200	-0.18774600	-1.38427200
H	2.27008900	-2.29817100	-1.54623500
H	-3.52935700	0.18792700	-1.38425900
H	-2.26993000	2.29829200	-1.54578600
H	-2.13361400	2.55236900	2.74326400
H	-3.45319500	0.46532500	2.90608900
H	5.52117800	4.84947900	1.07289000
H	3.49294900	4.15214500	2.33506100
H	2.66806900	1.82405900	2.11967900
H	8.10772900	0.36273500	-1.70017500
H	8.67999500	2.78041200	-1.72953400
H	7.32058700	4.37424800	-0.38739300
H	-8.10801500	-0.36168000	-1.70003000
H	-8.68070800	-2.77924900	-1.72985200
H	-7.32147100	-4.37360900	-0.38816200
H	-5.52203600	-4.84946200	1.07188800
H	-3.49359900	-4.15275100	2.33406300
H	-2.66835500	-1.82473400	2.11916300
H	-6.13837900	1.04741400	-2.63909400
H	-5.78994100	3.49055900	-2.67748600
H	-5.89596700	3.56655800	1.62286700
H	-6.25932600	1.12777600	1.65822900
H	5.78983500	-3.48971100	-2.67814100

H	6.13790800	-1.04651700	-2.63933400
H	6.25990200	-1.12774200	1.65794200
H	5.89691900	-3.56657500	1.62216900
H	5.95187600	-5.68478500	-1.40397600
H	5.79918800	-5.67850400	0.37276300
H	4.38600800	-5.33681100	-0.64616200
H	-5.95184800	5.68548700	-1.40253000
H	-5.79770200	5.67864100	0.37408200
H	-4.38541000	5.33705400	-0.64611100

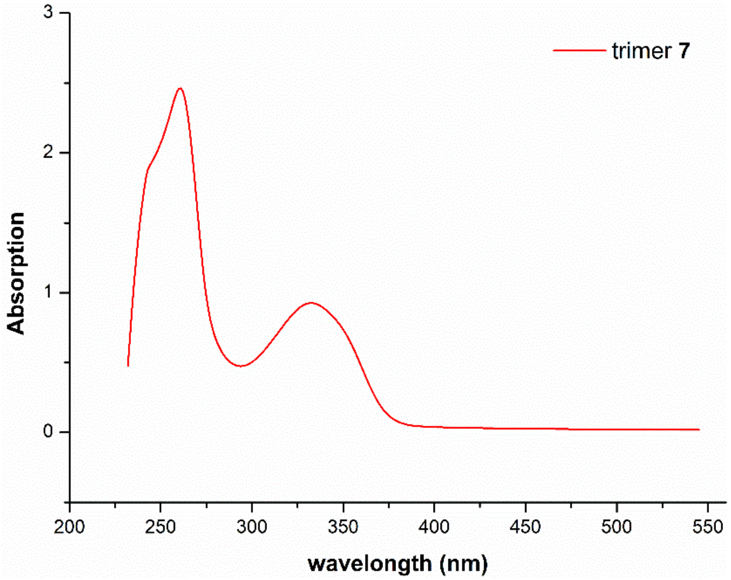

Figure S36. UV-vis spectrum of trimer **7**.

Table S26. Fluorescence lifetimes τ_f and adjusted R-Square of trimer **7** and polymer **1B**.

f_w / %	τ_f of 7/ns (Adj. R-Square)	τ_f of 1B /ns (Adj. R-Square)
**0**	45.66194 (0.98937)	48.07907 (0.9893)
**30**	47.69358 (0.98816)	47.97276 (0.98619)
**50**	47.6794 (0.98866)	50.00175 (0.98713)
**70**	49.0214 (0.98818)	47.2716 (0.98779)
**90**	57.81773 (0.98914)	47.67664 (0.98802)

Table S36. GPC data of polymer derived from Polymerization of bis(8-bromonaphthalen-1-yl)-derived monomer with BPin-thiadiazole

Chromatogram report

Header

Title		Data acquisition date and time	2020/03/14 02:42:27
Sample name	Li-3-13-mp9-1	Calculation date and time	2020/03/14 09:58:26
Database name	2020-03-13(2).chd	Acquisition time [min]	0.000 - 30.000
Data name	RSLT0017	Sampling interval [msec]	100
Method name	PS_calibration_120219	Cup number	9
Channel	RI UV	Calculation type	Molecular Weight Molecular Weight

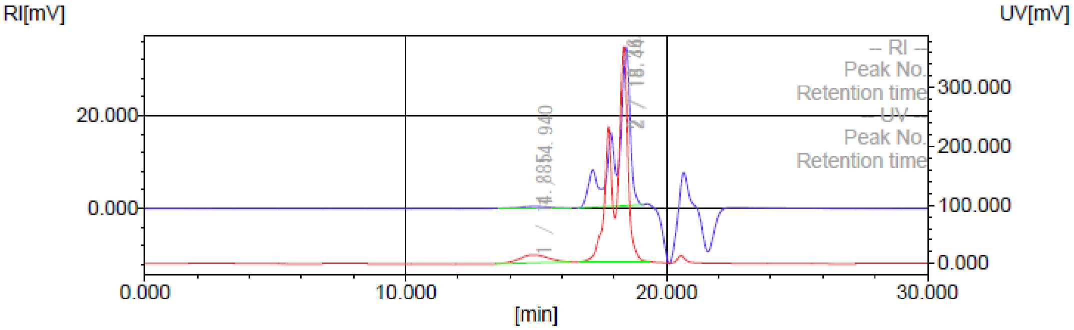

Result of molecular weight calculation (RI)

Peak 1 Base Peak

	[min]	[mV]	[mol]		
Peak start	13.558	0.014	62,093	Mn	10,149
Peak top	14.940	0.528	12,178	Mw	14,533
Peak end	16.327	0.084	2,374	Mz	20,517
				Mz+1	27,523
				Mv	14,533
Height [mV]			0.479	Mp	11,325
Area [mV*sec]			35.148	Mz/Mw	1.412
Height% [%]			1.388	Mw/Mn	1.432
[eta]			14532.93002	Mz+1/Mw	1.894

Peak 2 Base Peak

	[min]	[mV]	[mol]		
Peak start	16.603	0.133	1,713	Mn	258
Peak top	18.448	34.736	194	Mw	361
Peak end	19.047	0.868	96	Mz	546
				Mz+1	753
				Mv	361
Height [mV]			34.048	Mp	195
Area [mV*sec]			1276.647	Mz/Mw	1.513
Height% [%]			98.612	Mw/Mn	1.397
[eta]			360.80248	Mz+1/Mw	2.087

Result of molecular weight calculation (RI)

Total

	[min]	[mV]	[mol]		
Peak start	13.558	0.014	62,093	Mn	265
Peak top	18.448	34.736	194	Mw	741
Peak end	19.047	0.868	96	Mz	11,047
				Mz+1	26,896
				Mv	741
Height [mV]			34.527	Mp	195
Area [mV*sec]			1311.796	Mz/Mw	14.918
Height% [%]			100.000	Mw/Mn	2.792
[eta]			740.53161	Mz+1/Mw	36.320

Figure S36. Proton NMR (DMSO-D_6) of polymer derived from 2-(8-bromonaphthalen-1-yl)-4,4,5,5-tetramethyl-1,3,2-dioxaborolane

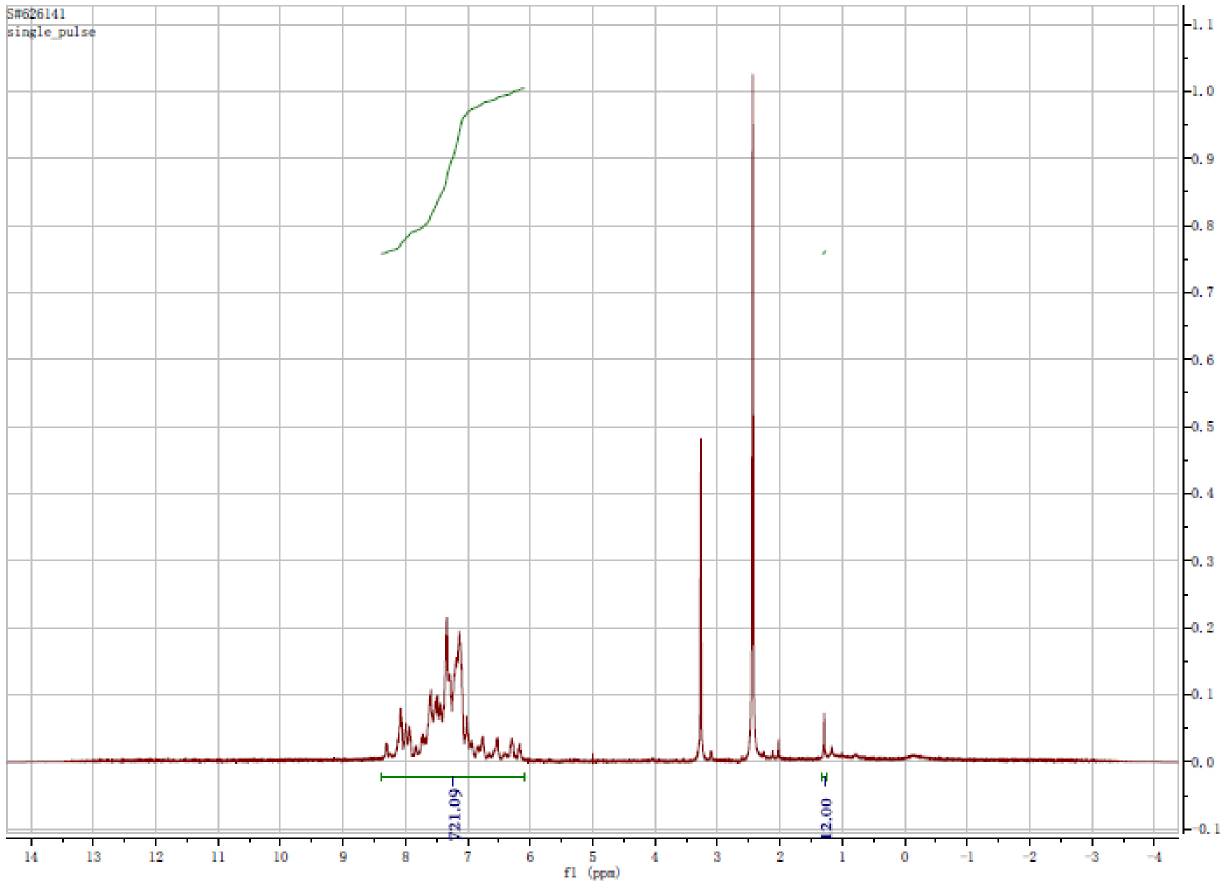

6. References

[1] (a)Vyskočil Š, Meca L, Tišlerová I, Císařová I, Polášek M, Harutyunyan SR, Belokon YN, Stead RM, Farrugia L, Lockhart SC (2002) 2, 8′-Disubstituted-1, 1′-Binaphthyls: A New Pattern in Chiral Ligands. *Chem Eur J* 8 (20):4633-4648. (b) Jurok R, Cibulka R, Dvořáková H, Hampl F, Hodačová J (2010) Planar chiral flavinium salts–Prospective catalysts for enantioselective sulfoxidation reactions. *Eur J Org Chem* 2010 (27):5217-5224.

[2] Ding X, et al. (2011) An n-channel two-dimensional covalent organic framework. *J Am Chem Soc* 133:14510-13.

[3] CrysAlis^{Pro} (2018) Oxford Diffraction Ltd.

[4] SCALE3 ABSPACK (2005) Oxford Diffraction Ltd.

[5] Sheldrick GM (2015) Crystal structure refinement with SHELXL. *Acta Crystallogr C: Struct Chem* 71:3-8.

[6] Møller C, Plesset MS (1934) Note on an approximation treatment for many-electron systems. *Phys Rev* 46:618.

[7] Jung Y, Lochan RC, Dutoi AD, Head-Gordon M (2004) Scaled opposite-spin second order Møller–Plesset correlation energy: an economical electronic structure method. *J Chem Phys* 121:9793-9802.
[8] Hellweg A, Grün SA, Hättig C (2008) Benchmarking the performance of spin-component scaled CC2 in ground and electronically excited states. *Phys Chem Chem Phys* 10:4119-4127.
[9] Schirmer J (1982) Beyond the random-phase approximation: A new approximation scheme for the polarization propagator. *Phys Rev A* 26:2395.
[10] Trofimov A, Schirmer J (1995) An efficient polarization propagator approach to valence electron excitation spectra. *J Phys B: At Mol Opt Phys* 28:2299.
[11] Weigend F, Ahlrichs R (2005) Balanced basis sets of split valence, triple zeta valence and quadruple zeta valence quality for H to Rn: Design and assessment of accuracy. *Phys Chem Chem Phys* 7:3297-3305.
[12] Hättig C (2005) Structure optimizations for excited states with correlated second-order methods: CC2 and ADC (2). *Adv Quantum Chem* 50:37-60.
[13] Klamt A (2011) The COSMO and COSMO-RS solvation models. *Wires Comput Mol Sci* 1: 699-709.
[14] Lunkenheimer B, Köhn A (2013) Solvent effects on electronically excited states using the conductor-like screening model and the second-order correlated method ADC (2). *J Chem Theory Comput* 9:977-994.
[15] Tomasi J, Mennucci B, Cammi R (2005) Quantum mechanical continuum solvation models. *Chem Rev* 105:2999-3094.
[16] Baird ZS, Uusi-Kyyny P, Pokki J-P, Pedegert E, Alopaeus V (2019) Vapor pressures, densities, and PC-SAFT parameters for 11 bio-compounds. *Int J Thermophys* 40:102.
[17] Chai J-D, Head-Gordon M (2008) Long-range corrected hybrid density functionals with damped atom–atom dispersion corrections. *Phys Chem Chem Phys* 10:6615-6620.
[18] Furche F, Ahlrichs R (2002) Adiabatic time-dependent density functional methods for excited state properties. *J Chem Phys* 117:7433-7447.
[19] Caricato M, et al. (2006) Formation and relaxation of excited states in solution: A new time dependent polarizable continuum model based on time dependent density functional theory. *J Chem Phys* 124:124520.
[20] Plasser F, Lischka H (2012) Analysis of excitonic and charge transfer interactions from quantum chemical calculations. *J Chem Theory Comput* 8:2777-2789
[21] Ahlrichs R, Bär M, Häser M, Horn H, Kölmel C (1989) Electronic structure calculations on workstation computers: The program system turbomole. *Chem Phys Lett* 162:165-169.
[22] Frisch MJ, et al. (2009) GAUSSIAN09. *Inc., Wallingford, CT, USA* 121:150-166.
[23] Martin RL (2003) Natural transition orbitals. *J Chem Phys* 118:4775-4777.
[24] Plasser F, Wormit M, Dreuw A (2014) New tools for the systematic analysis and visualization of electronic excitations. I. Formalism. *J Chem Phys* 141:024106.
[25] Plasser F, available from http://theodore-qc.sourceforge.net.
